# The retinoic acid family-like nuclear receptor SmRAR identified by single-cell transcriptomics of ovarian cells controls oocyte differentiation in *Schistosoma mansoni*

**DOI:** 10.1093/nar/gkae1228

**Published:** 2024-12-16

**Authors:** Max F Moescheid, Zhigang Lu, Carmen Diaz Soria, Thomas Quack, Oliver Puckelwaldt, Nancy Holroyd, Pauline Holzaepfel, Simone Haeberlein, Gabriel Rinaldi, Matthew Berriman, Christoph G Grevelding

**Affiliations:** Institute of Parasitology, Justus Liebig University, Schubertstrasse 81, 35392 Giessen, Germany; Institute of Parasitology, Justus Liebig University, Schubertstrasse 81, 35392 Giessen, Germany; Wellcome Sanger Institute, Wellcome Genome Campus, Hinxton CB10, 1SA, UK; Wellcome Sanger Institute, Wellcome Genome Campus, Hinxton CB10, 1SA, UK; Institute of Parasitology, Justus Liebig University, Schubertstrasse 81, 35392 Giessen, Germany; Institute of Parasitology, Justus Liebig University, Schubertstrasse 81, 35392 Giessen, Germany; Wellcome Sanger Institute, Wellcome Genome Campus, Hinxton CB10, 1SA, UK; Institute of Parasitology, Justus Liebig University, Schubertstrasse 81, 35392 Giessen, Germany; Institute of Parasitology, Justus Liebig University, Schubertstrasse 81, 35392 Giessen, Germany; Wellcome Sanger Institute, Wellcome Genome Campus, Hinxton CB10, 1SA, UK; Department of Life Sciences, Aberystwyth University, Penglais, Aberystwyth, Ceredigion, SY23 3DA, UK; Wellcome Sanger Institute, Wellcome Genome Campus, Hinxton CB10, 1SA, UK; School of Infection and Immunity, College of Medicine, Veterinary and Life Sciences, University of Glasgow, 120 University Place, Glasgow G12 8TA, UK; Institute of Parasitology, Justus Liebig University, Schubertstrasse 81, 35392 Giessen, Germany

## Abstract

Studies on transcription regulation in platyhelminth development are scarce, especially for parasitic flatworms. Here, we employed single-cell transcriptomics to identify genes involved in reproductive development in the trematode model *Schistosoma mansoni*. This parasite causes schistosomiasis, a major neglected infectious disease affecting >240 million people worldwide. The pathology of schistosomiasis is closely associated with schistosome eggs deposited in host organs including the liver. Unlike other trematodes, schistosomes exhibit distinct sexes, with egg production reliant on the pairing-dependent maturation of female reproductive organs. Despite this significance, the molecular mechanisms underlying ovary development and oocyte differentiation remain largely unexplored. Utilizing an organ isolation approach for *S. mansoni*, we extracted ovaries of paired females followed by single-cell RNA sequencing (RNA-seq) with disassociated oocytes. A total of 1967 oocytes expressing 7872 genes passed quality control (QC) filtering. Unsupervised clustering revealed four distinct cell clusters: somatic, germ cells and progeny, intermediate and late germ cells. Among distinct marker genes for each cluster, we identified a hitherto uncharacterized transcription factor of the retinoic acid receptor family, SmRAR. Functional analyses of SmRAR and associated genes like Sm*meiob* (meiosis-specific, oligonucleotide/oligosaccharide binding motif (OB) domain-containing) demonstrated their pairing-dependent and ovary-preferential expression and their decisive roles in oocyte differentiation of *S. mansoni*.

## Graphical Abstract

**Graphical Abstract:**
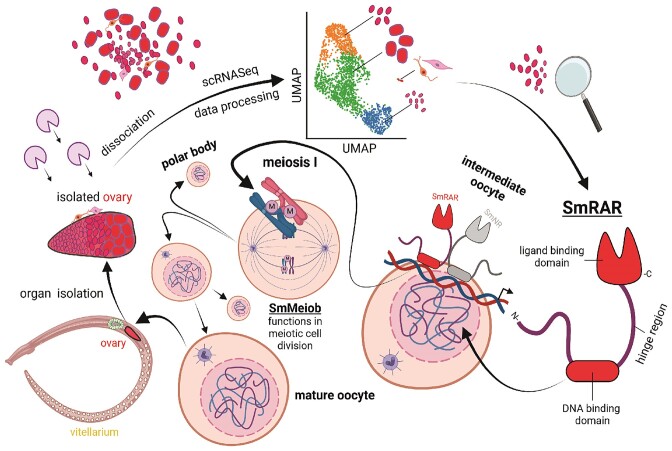

## Introduction

Members of the nuclear receptor (NR) superfamily are ligand-activated transcription factors (TF) that play key roles in cell differentiation, proliferation and metabolism (1–3). Little is known about NRs in flatworms (platyhelminths), a phylum comprising a broad spectrum of bilaterally symmetrical, soft-bodied invertebrates with free-living and parasitic life styles (3–9).

All parasitic members of this phylum are exposed to diverse environments and display complex life cycles, some of which involve several hosts. Understanding their biology has been a challenge, largely unmet for non-model organisms such as parasitic flatworms. Among these is *Schistosoma mansoni*, a blood fluke that infects humans and animals. Alongside other schistosome species, *S. mansoni* causes schistosomiasis (bilharzia), one of the major Neglected Tropical Diseases (10). This infectious, zoonotic disease affects millions of people worldwide, mostly in the global south (11–13). However, recent outbreaks in Southern Europe point to the possibility of schistosomiasis spreading towards moderate climate zones (14).

Schistosomes exhibit a complex biology with different larval and adult life stages including an intermediate snail host and a vertebrate definitive host. An unusual feature of schistosome biology is the requirement of the adult female for constant pairing with the male partner to complete gonad differentiation and reach sexual maturity (15,16). Another peculiarity of trematode reproduction, including *S. mansoni*, is the deposition of composite eggs consisting of a single fertilized oocyte (zygote) and 30–40 vitellocytes (yolk cells), which are all surrounded by a resistant eggshell (17–20). Failure in egg formation can result in the production of deformed eggs and the loss of egg viability respectively embryogenesis (18,20,21). Furthermore, schistosome reproductive biology has been the subject of numerous physiological and molecular studies for many years (15,22–27). Although a recent report demonstrated a decisive role of a male-derived peptide-based pheromone in the female maturation process in *S. mansoni* (28), mechanisms underlying male-female interaction and male-induced female sexual maturation have not yet been fully solved. Understanding schistosome sexual biology may lead to novel insights into the pathology of schistosomiasis, which is determined by the eggs (13). Not all schistosome eggs reach the environment via urine (in case of *Schistosoma haematobium*) or feces (e.g. *S. mansoni, Schistosoma japonicum* and further species); many remain in the vasculature and lodge in spleen and liver where they cause severe inflammation, liver fibrosis and granuloma formation (13). There is also evidence of an association of urogenital schistosomiasis (UGS) and squamous cell carcinoma of the bladder, consequence of trapped *S. haematobium* eggs in the urothelium of the bladder (29). UGS is classified as group 1 biological carcinogen by International Agency for Research on Cancer (30). In addition, recent reports suggest that *S. mansoni* egg-secreted molecules manipulate the tissue environment that might ultimately promote hepatic and/or colorectal cancer (31).

To better understand the sexual biology of schistosomes, comparative transcriptomics of adult schistosomes and their (isolated) gonads uncovered >3,000 genes significantly differentially transcribed between ovaries of immature (= unpaired) and mature (= paired) females (24). However, this bulk RNA-seq approach failed to distinguish gene expression differences among sub-populations of cells within gonad tissues. To this end, a first whole organism-based single-cell atlas identified gonad-associated cell populations of female and male *S. mansoni*, including developing gonadal cells (27,32). Since the authors used whole worms as starting material, they captured small cell numbers in certain clusters (e.g. 20–79 cells in late female germ cell clusters), and thus may have missed cells at intermediate stages.

Here, we used the organ isolation approach previously established for *S. mansoni* (33) to extract ovaries from sexually mature females. To explore the ovary transcriptome at single cell level, we used the 10× chromium single-cell RNA sequencing (scRNA-seq) technology (34). Since the ovary of a paired female contains both immature oogonia (iO) and mature oocytes (mO) that have entered meiosis, we expected to identify genes contributing to developmental processes from the oogonia (gonadal stem cell-like) stage to mature oocytes. Bioinformatics analyses uncovered distinct cell clusters and associated marker genes for different developmental stages of oocytes. Our analysis augmented previous RNA-seq data by identifying novel ovary-associated genes. Furthermore, we have identified and functionally characterized candidate genes potentially involved in oocyte differentiation, in particular, a potential member of the retinoic acid receptor (RAR) family of NRs, denoted as SmRAR. Although previous studies pointed to the existence of members of the NR superfamily in *S. mansoni*, such as retinoid-X receptor (RXR) orthologs binding to the promoter of an egg-shell precursor protein (4,8,35), an androstane receptor homologue (36), two thyroid hormone receptors (37), a vitellogenic factor 1 (VF1) involved in the development of the female vitellarium following pairing (26) and the fushi tarazu ortholog SmFTZ-F1 as one key regulator of esophageal tissue homeostasis (3), current knowledge on NRs in *S. mansoni* or other platyhelminths remains rather limited (8).

Based on this new subtranscriptome data set of the schistosome ovary, we identified Sm*rar* (Smp_144170) as a NR transcribed in intermediate-stage oocytes. Functional characterization by RNA interference (RNAi) of SmRAR and biologically associated molecules such as Sm*meiob* (38,39), the first reported MEIOB ortholog of a helminth species, strongly points toward essential roles of these genes in oocyte maturation and meiosis progression in *S. mansoni*.

## Materials and methods

### Maintenance of the *S. mansoni* life cycle and ethics statements

The complete life cycle of the *S. mansoni* Naval Medical Research Institute (NMRI); Puerto Rican strain was maintained at the Wellcome Sanger Institute (WSI) by breeding and infecting susceptible *Biomphalaria glabrata* snails and mice (outbred TO strain). The mouse experimental infections and other regulated procedures were conducted under the Home Office Project Licence No. P77E8A062 held by G.R. All protocols were revised and approved by the Animal Welfare and Ethical Review Body (AWERB) of the WSI. The AWERB is constituted as required by the UK Animals (Scientific Procedures) Act 1986 Amendment Regulations 2012.

Experiments involving hamsters at the Justus Liebig University Giessen were performed in agreement with the European Convention for the Protection of Vertebrate Animals used for Experimental and other Scientific Purposes (ETS No. 123; revised Appendix A) and have been approved by the Regional Council (Regierungspraesidium) Giessen (V54‐19c 20 15h 02 GI18/10).

To obtain adult parasites for ovary isolation, mice were infected with 250 (mixed-sex) cercariae intraperitoneally, and adult schistosome worms harvested by hepatoportal perfusion 43 days post-infection and transferred to a 50 ml-Falcon tube for each mouse. After the worms sank down, the supernatants were removed and perfusates were combined and filtered through a 40 μm cell strainer. Worms were washed twice with Dulbecco’s modified Eagle’s medium (DMEM) (Gibco, USA), and transferred into a Petri dish containing 10 ml DMEM medium supplemented with 10% Fetal Bovine Serum (FBS), Sigma–Aldrich, Germany, 10 mM 4-(2-hydroxyethyl)-1-piperazineethanesulfonic acid (HEPES), 100 U/ml penicillin and 100 μg/ml streptomycin (Thermo Fisher Scientific, UK). The worms were kept at 37°C and 5% CO_2_ for one hour before proceeding with ovary isolation.

### Preparation of single-cell suspensions of isolated ovaries

For the isolation of complete ovaries, *S. mansoni* couples were separated by pipetting, and females (100–110) were collected and processed as described by Hahnel *et al*. (33). Briefly, worms were washed twice with M199 medium (Gibco, USA) and centrifuged for 2 × 5 min in 400 μl TS-solution at 1,200 rpm. Worms were washed in 2 ml M199 and then incubated in 300 μl elastase solution (5 U/ml in non-supplemented M199) at 37°C with agitation at 650 rpm for about 20 min. Enzymatic digestion was monitored with an inverted microscope. As soon as tissue degradation led to the disintegration of the tegument and subtegumental muscle tissue, ovaries were released from the worm carcasses, immediately collected by pipetting, and transferred into 10 μl DMEM (Gibco, USA) medium containing 10% FBS (Sigma–Aldrich, Germany). The isolated ovaries were then transferred into an Eppendorf tube containing 40 μl 0.25% trypsin (without ethylenediaminetetraacetic acid) solution and incubated at 37°C for 15 min. Tissue suspensions were pipetted several times to facilitate cell release. Afterwards, 72 μl DMEM and 18 μl DMEM containing 10% FBS were added to make a 140 μl cell suspension (approximately 500 cells/μl, estimated from 69 ovaries x 10^3^ cells/ovary in 140 μl) containing 2% FBS for preparing the 10X library.

### Microscopy of isolated oocytes

Trypsin treatment of ovaries was monitored by bright-field microscopy (Hoffman modulation contrast microscopy, HCM; Olympus IX 81), and the obtained cells imaged by scanning electron microscopy (SEM), as described before (40). For SEM, we transferred oocytes onto 12 mm glass slides, which were precoated with poly-l-lysine (PLL), laminin and sterilized with 100% ethanol. For coating, 50 μl of PLL (0.05 mg/ml in dH_2_O; Biochrom AG, Germany) was pipetted in the center of a round glass slide, which was subsequently placed on a heating plate (37°C) for liquid evaporation. We then placed 50 μl laminin (16 μg/ml in M199; Sigma–Aldrich, Germany) onto the PLL layer and dried the slide at room temperature (RT°C) for 40 min. Superfluous liquid was discarded and the slide kept at 4°C until use. For adherence, the cell suspension (100 μl) was placed on top of the PLL-laminin double layer and incubated at RT°C for 20 min. Cells were then fixed in 2% glutaraldehyde in 0.1 M phosphate-buffered saline (PBS) (pH 7.4) at RT°C for 2 h with shaking. This was followed by washing (10 min) in 0.1 M PBS, osmium fixation in 2% OsO_4_ (Serva, Germany) at RT°C (30 min) and two washes (5 min each) in double distilled H_2_O. The probes were dehydrated in 30% and 50% ethanol on ice, with shaking and stored in 70% ethanol at 4°C over night. The slides were dehydrated by incubating in 80%, 90%, 96% and 100% ethanol (10 min each), on ice, with shaking. Afterwards, the probes were critical point-dried (cpd030, Bal-tec, Germany), mounted on SEM-holders and sputtered with gold (scd004, Balzers, Germany). Imaging was performed with a FEG scanning electron microscope (DSM982, Zeiss, Germany) at 3 kV. Images were recorded using a secondary electron-detector, with the voltage of the collector grid biased to +300 V to improve the signal-to-noise ratio and to achieve an optimal topographical contrast.

### 10X Genomics library preparation and sequencing

The 10X Genomics protocol was followed to create gel in emulsion beads (GEMs) containing single cells, hydrogel beads and reagents for reverse transcription (RT). We performed barcoded complementary DNA (cDNA) synthesis, clean-up and amplification, followed by library preparation as described in ‘Single Cell 3′ Reagent Kits v2 User Guide’ (10X Genomics, USA). The libraries were sequenced on an Illumina Hiseq4000 (paired-end reads 75 bp), using one sequencing lane per sample. All raw sequence data was deposited in the European Nucleotide Archive (ENA) under the project accession ERP137193, ERS11891010 (bisex ovaries; mO).

### Mapping and quality control of 10X scRNA-seq data

Single-cell RNA-seq data were mapped to version 7 of *S. mansoni* genome on WormBase Parasite version 14 (41) using the 10X Genomics analysis pipeline Cell Ranger (v2.2.0). We relied on the default cut-off provided by Cell Ranger to exclude empty droplets. Filtered matrix was used for downstream clustering analysis.

### Clustering analysis

The R package Seurat v3.2.2 (35) was used to process the single cell expression data. We applied extra filters to exclude low quality cells: for the oocytes, we used nFeature_RNA 550 – 3,000 & nCount_RNA 750 – 15,000 & percent.mt < 20. We kept cells expressing high mitochondrial (mt) transcripts (mostly somatic) because they expressed >1,000 genes and exhibited no mt genes as cluster markers. Subsequently, the data were log-normalized using default options, and 2,000 highly variable genes were identified using the *FindVariableGenes* function. We then ran the command *RunPCA* with default options for dimensionality reduction and FindNeighbors with dims = 1:20 to construct the neighbourhood graph. Finally, cell clusters were determined using *FindClusters* with res = 0.2. Cluster marker genes were identified using *FindAllMarkers* with the following parameters: only.pos = TRUE, min.pct = 0.25, logfc.threshold = 0.5 and p_val < 1e^−20^. Cluster markers were manually inspected and compared to the findings of Wendt *et al*., (27) as well as literature to determine their identities. The clustering data can be interactively explored at https://gonadsc.schisto.xyz/, and the processed Seurat object will be provided upon request.

### Gene ontology enrichment analysis

Gene Ontology (GO) annotation was obtained from WormBase Parasite version 14 (https://parasite.wormbase.org) (41). GO term enrichment was performed using the weight01 method provided in topGO v2.38.1 (42) for categories Biological Process (BP) and Molecular Function (MF). For each category, the analysis was restricted to terms with a node size of at least 5. Fisher’s exact test was applied to assess the significance of over-represented terms compared with all expressed genes. The threshold was set as False Discovery Rate (FDR) < 0.05.

### *In vitro* culture and RNAi of *S. mansoni*

Adult *S. mansoni* of the Liberian strain were obtained from hamsters (*Mesocricetus auratus*) as final hosts by perfusion (43). Directly following perfusion, we cultured worms in 5 ml M199(3+) (Gibco, Germany), supplemented with 1% (*v/v*) antibiotic-antimycotic solution (CCPro, Germany), 1% (*v/v*) HEPES buffer (Carl Roth, Germany; pH 7.4) and 10% (*v/v*) fetal calf serum (Sigma–Aldrich, Germany) per 3 cm Petri dish (Greiner Bio-One, Germany) in a CO_2_ incubator (Galaxy S+, RS Biotech; Germany) at 37°C and 5% CO_2_. Adult worms adapted for one day to the *in vitro* culture conditions before experiments started. For each biological replicate, 10 *S. mansoni* couples (obtained from one hamster) were transferred in one well of a 6-well plate (Greiner Bio-One, Germany), containing 3 ml of prewarmed (37°C) M199 (3+) medium.

For re-pairing experiments, a modified version of the recently described ABC169 medium (26) was used as described by Li *et al*. (44). In brief, ABC169/LDL is based on the Basch medium (45) with the addition of 1% (*v/v*) antibiotic-antimycotic solution, 10% (*v/v*) fetal calf serum, 200 μM ascorbic acid (Sigma–Aldrich, Germany), 0.2% (*v/v*) human red blood cells (10% suspension; Biochrome, Germany) and 0.25% (*v/v*) low-density lipoprotein (LDL, Trina Bioreactives; Switzerland). Worms were kept in 12-well plates (Greiner, Germany) in 3 ml prewarmed ABC169/LDL medium at 37°C and 5% CO_2_. For repairing experiments, 10 pairing-inexperienced females (single-sex females; sF) obtained from one hamster for each biological replicate were placed in a well of a 12-well plate and cultivated for 7 days. For this, worms from one hamster were considered as biological replicate. For repairing, 15 pairing-experienced males (bisex males; bM), which had been mechanically separated from their original partners, were added to the sF group to reach a female–male ratio of 1:1.5, which had been shown before to be optimal for re-pairing under these conditions (32). After 72 h, supernumerary males and unpaired worms were removed, and only females from stably paired couples were used for RNA extraction (five females each) and microscopic analysis (remaining females), as described below.

For RNAi experiments, double-stranded RNA (dsRNA) molecules against the target gene or controls, as indicated in the ‘Results’ section, were added in a concentration of 30 μg ml^−1^ per experimental group starting at day 1 after perfusion, and the parasites were cultured for 15 days (pairing-experienced couples) or 20 days (re-pairing experiments). Medium was replaced every 2–3 days, along with adding fresh dsRNA. Worm viability was monitored regularly, along with the medium exchange, as described before (46). Phenotypes were assessed blinded using an inverse laboratory microscope (DM IL LED, Leica Microsystems; Germany) and classified using the following scheme: 0, total absence of movement; 1, only gut movements or occasional movement of head and tail; 2, reduced motility; 3, normal activity; and 4, hyperactivity (46). Attachment of worms to the Petri dish, pairing status (either paired or separated) and oviposition were determined next to morphological changes. Eggs were defined as deformed by considering the alteration in shape (e.g. absence of the characteristic spike), egg size and composition as illustrated in Supplemental Figure S1.

### Retinoic acid-treatment and inhibition of retinoic acid-signaling

To determine the effects of 9-*cis-*retinoic acid (RA) on *S. mansoni* reproduction, couples were cultured for 72 h with RA. For this, 10 couples each were cultured in 3 ml M199(3+) supplemented with 5 μM RA (Sigma–Aldrich, Germany), which corresponds to the concentration of 9-*cis-*RA in human blood plasma (47,48). The RA stock solution was prepared at a concentration of 10 mM in Dimethyl Sulfoxide (DMSO). Couples in the control group received DMSO equivalent to RA-treated worms. RA was renewed daily by changing the medium. RA solutions as well as RA-containing media were kept safe from direct light exposure at all times. In addition, to antagonize RA-dependent signaling by HX531 (4-(5 H-2,3-(2,5-dimethyl-2,5-hexano)-5-methyl-8-nitrodibenzo[b,e] [1,4] diazepin-11-yl; Bio-Techne R&D Systems, USA), a potent RXR antagonist (49,50), 10 couples per biological replicate were treated with 1 μM and 10 μM of this inhibitor for 72 h in M199(3+). The HX531 stock solution was prepared at a concentration of 10 mM in DMSO, and HX531 was renewed daily by changing the medium. Pairing rate and egg production were recorded daily.

### EdU cell-proliferation assay and confocal laser scanning microscopy

For the determination of cell proliferation (51), EdU (5-ethinyl-2′-desoxyuridin) was added in a final concentration of 10 μM, 24 h before the end of the *in vitro*-culture period. Subsequently, couples were selected for EdU staining as follows: couples were first separated by adding 0.25% (*w/v*) tricaine (ethyl 3-aminobenzoate methanesulfonate; Sigma–Aldrich, Germany) as described before (52). Female and male worms were separately collected in 1.5 ml reaction tubes and washed with 1× PBS. The supernatant was discarded, and then the worms were fixed with 4% paraformaldehyde in 1× PBS, bleached for 1 h under bright light in a 1 ml solution of 1.2% H_2_O_2_ (*v/v*) (Carl Roth, Germany), 5% deionized formamide (*v/v*) (Carl Roth; Germany) in 0.5× Saline-Sodium Citrate (SSC), before staining using the Click-iT Plus EdU Alexa Fluor 488 imaging kit (Thermo Fisher Scientific, USA) and with Hoechst 33 342, as previously described (53–55). In order to quantify cell proliferation in the immature part of the ovary, the surface area of the maximum cross-sectional area of ovaries was determined by the ImageJ measurement function applying the selection brush tool (56–58), and the number of proliferating EdU-positive oogonia was counted. Finally, the ratio of EdU-positive cells per mm^2^ was calculated as a measure of cell proliferation.

RNAi-induced morphological alterations of the reproductive organs of either female or male worms were assessed by confocal laser scanning microscopy (CLSM), starting with the fixation of couples in Alcohol Formalin Glacial Acetic Fixative (AFA; 66.5% ethanol, 1.1% paraformaldehyde, 2% glacial acetic acid) and staining with CertistainH carmine red (Merck, Germany), as described (23,59). For destaining, the worms were incubated three times in acidic ethanol [70% (*v/v*), ethanol, 2.5% (*v/v*), hydrochloric acid (Carl Roth, Germany)] for a total of 5–10 min. Next, the worms were dehydrated in 80%, 90% and 100% ethanol for 5 min each, before fixing on a slide in Canada balsam (Sigma–Aldrich, Germany) (59). For microscopy, a TCS SP5 vis CLSM (Leica Microsystems, Germany) was used. AlexaFluor488 and carmine red were excited using an argon-ion laser at 488 nm, and Hoechst 33 342 at 405 nm. Background signals and optical section thickness were defined by setting the pinhole size to airy unit 1 (60).

To determine the ovarian volume, CLSM was performed to measure the volume of the part of the ovary containing only mature oocytes from carmine red-stained females (TCS SP5 vis confocal laser scanning microscope, Leica Microsystems, Germany). For this, Z-stacks were created, and pictures analyzed using the LasAF software (Leica Microsystems, Germany). The maximum extension of the ovary on the z-axis (3D image) was determined microscopically in order to define the lower and upper ends of the ovary as the Z-stack delimitation. A total of 15 single images each were taken along the z-axis, in which the distances between the individual planes remained constant for each sample. The surface of the area containing mature oocytes was determined using the ImageJ measurement function by applying the selection brush tool (56–58). Subsequently, the resulting surface area (μm^2^) of each image was multiplied by the defined Z-stack travel distance (μm) of each individual image. The sum of all volumes obtained for each individual plane resulted in the approximate total volume (μm^3^) of each ovary.

Microscopic images were processed with LasX software (Leica Microsystems, Germany), and stitching was performed using the ImageJ Mosaic plugin (61,62). Graphics were created using GraphPad Prism V.8 (GraphPad Software; San Diego, USA).

### Lipid staining

Oil-Red O (Sigma–Aldrich, Germany) was used to stain the lipid-rich vitelline droplets in the vitellarium of mature *S. mansoni* females. For this, a modified Oil-Red O staining protocol (26,63) was used. Couples were separated using 0.25% tricaine (Sigma–Aldrich, Germany) in 1× PBS and washed twice in 1× PBS. Fixation with 2% paraformaldehyde (Roth, Germany) in PBSTx (1× PBS + 0.3% Triton-X-100; Sigma–Aldrich, Germany) was performed overnight at 4°C on a shaker at 135 rpm. Afterwards, the worms were washed twice in 1× PBSTx and incubated in 99% propane 1,2-diol (Sigma–Aldrich, Germany) for 5 min at RT°C (10 worms/ml). The worms were allowed to settle, and the supernatant was replaced by an equal volume of 0.5% (*w/v*) Oil-Red O (powder; Sigma–Aldrich, Germany) dissolved in propane 1,2-diol. Staining was performed on a shaker at 135 rpm for 45 min at RT°C. The Oil-Red O solution was then replaced with an equal volume of 85% propane 1,2-diol, and the solution agitated for 5 min. This step was repeated twice. Finally, the worms were washed with 1× PBS and embedded in ROTI Mount FluorCare (Carl Roth, Germany) for immediate microscopic analysis. Phase contrast microscopy was performed with an inverted laboratory microscope (DM IL LED; Leica Microsystems, Germany).

### Cloning of constructs for dsRNA synthesis

For dsRNA synthesis, a T7 promoter-driven construct was cloned into the pJC53.2 plasmid vector (64). For this, 1 μg of pJC53.2 plasmid DNA was digested by *Ahd*I (New England Biolabs; NEB, UK) in a total volume of 50 μl containing 1× CutSmart buffer (NEB, UK) at 37°C for 2 h. The resulting DNA fragments were separated by agarose gel electrophoresis and extracted using the Monarch DNA Cleanup and Gel Extraction Kit (NEB, UK).

Amplicons of 500 bp were obtained by polymerase chain reaction (PCR), using a final reaction volume of 20 μl, including 100 ng *S. mansoni* cDNA generated by reverse transcription of extracted total RNA of couples (QuantiTect Reverse Transcription Kit, Qiagen, Hilden, Germany). For PCR, 1 μM of each primer targeting respective transcripts (Supplemental Table S1), and the recommended concentration for the Q5 High-Fidelity Polymerase kit (NEB, UK) were used. Template cDNA was initially denatured at 98°C for 3 min, followed by 35 cycles consisting of denaturation at 95°C for 30 s, primer annealing at 60°C for 20 s, and elongation at 72°C for 45 s (S1000 Thermal Cycler; Bio-Rad, USA). Aliquots of generated PCR products were analyzed by agarose gel electrophoresis. Afterwards, amplicons were extracted from the gel using the Monarch DNA Cleanup and Gel Extraction Kit and eluted in a finale volume of 20 μl elution buffer. An additional PCR step generated 3′ A-overhangs (AccuPrime Taq DNA Polymerase High Fidelity kit; Invitrogen, USA), with the 20 μl of fragment-containing eluate as a template, with its corresponding primers. Template DNA was initially denatured at 98°C for 3 min, followed by five 3′ A-overhang-generating amplification cycles, which consisted of denaturation at 95°C for 30 s, primer annealing at 58°C for 30 s and elongation at 67°C for 5 min (S1000 Thermal Cycler; Bio-Rad, USA). The resulting fragments were cleaned-up as described before.

Afterwards, the fragments were ligated into pJC53.2 using T4 ligase (NEB, UK) as described in the manufacturer’s manual (Ligation Protocol with T4 DNA Ligase, NEB, UK). Recombinant plasmids were transformed into *Escherichia coli* DH5α (NEB, UK) by heat shock and selected by kanamycin- and ampicillin-containing Lysogeny Broth (LB) plates at 37°C (overnight). The sequence integrity of plasmid inserts of selected clones were verified by Sanger sequencing (Microsynth SeqLab, Germany). Resulting plasmids were then used as basis for dsRNA and riboprobe synthesis (Supplemental Table S2).

### Synthesis of dsRNAs and WISH-probes

For dsRNA and riboprobe [whole-mount *in-situ* hybridization (WISH)-probe] syntheses, previously amplified PCR products derived from each pJC53.2 construct, carrying gene-specific 500 bp fragments (Supplemental Table S2), were used as templates. In addition, for the synthesis of an irrelevant non-schistosomal dsRNA control, we used a 500 bp fragment encoding the *E. coli* ampicillin resistance gene (*ampR*, Supplemental Table S2), which has been described to be a suitable dsRNA control for the experimental conditions of this study (65,66). Synthesis of *ampR* dsRNA was carried out as described by Moescheid and Puckelwaldt *et al*. (65). The inserts were amplified using a primer specific for the pJC53.2 T7-promoter sequence (Q5 High-Fidelity Polymerase, NEB, UK; T7_extended 5′-CCT AAT ACG ACT CAC TAT AGG GAG-3′). Approximately 0.5 μg PCR product was used for *in vitro* transcription modified after Collins *et al*. (64). The reaction mixture contained 10 μl transcription buffer (10×), 20 μl rNTP mix (NEB, UK), 10 μg T7 RNA polymerase (expression vector kindly provided by James Collins, UT Southwestern) and 1 μl inorganic pyrophosphatase (NEB, UK), supplemented with Diethyl Pyrocarbonate (DEPC)-treated water to 100 μl total volume. The reactions were carried out for approximately 16 h at 37°C, followed by a DNase I treatment (10 U; NEB, UK, 37°C) for 30 min.

For WISH-probe synthesis, 0.5 μg PCR product was used for the *in-vitro* transcription of single-stranded RNA probes (ssRNA). First, a DIG-rNTP-mixture was prepared, which contained 5 μl of each ATP, GTP, CTP (100 mM, NEB), 3.5 μl UTP (100 mM, NEB) and 17.5 μl DIG [Digoxigenin-X-(5-aminoallyl)]-UTP (10 mM; Jena Bioscience, Germany). Next, the ssRNA *in vitro*-transcription reaction mixture was prepared containing 1 μl of either T3 or SP6 RNA-polymerase (Roche, Switzerland), 2 μl 10× transcription buffer (Roche, Switzerland), 2 μl of DIG-rNTP mixture and 0.6 μl murine RNase inhibitor (NEB, UK) in a final volume of 20 μl. The reactions were carried out for approximately 16 h at 28°C, followed by a DNase I treatment (10 U; NEB, 37°C) for 30 min. RNA was precipitated using 7.5 M LiCl_2_ and cleaned from the reaction mixture. Afterwards, the RNA was resuspended in DEPC-treated water and incubated for 3 min at 72°C. Subsequently, the concentration was determined by photometric measurement and the synthesis of dsRNA confirmed by agarose gel electrophoresis.

### Whole-mount *in-situ* hybridization

WISH was performed as previously described (44,52,67,68) with the following modifications. For permeabilization, the parasite tissue of male schistosomes was treated with 15 μg ml^−1^ and that of females with 7.5 μg ml^−1^ proteinase K (Ambion, UK). In total, up to 40 parasites were treated with protease K containing RNase-free PBStx [1× PBS in RNase free DEPC-water, 0,1% TritonX-100 (*v/v*); Sigma–Aldrich, Germany] in a final volume of 10 ml for 50 min and shaking at 135 rpm at RT°C. For hybridization, the worm samples were incubated with the appropriate WISH-probes at a concentration of 200 ng ml^−1^ each, in a final volume of 300 μl and shaken at 135 rpm for at least 16 h at 55°C (GFL, Germany). For detection, an anti-DIG-AP antibody (1:2,000, Merck, Germany, 11 093 274 910) was incubated in colorimetric blocking solution [7.5% heat-inactivated horse serum (#H1138, Merck; Germany) in Tris-NaCl-Tween-20 buffer solution, pH 8.0] overnight at 4°C and developed with nitroblue tetrazolium (No. 14 799 526, Roche; Switzerland) and 5-bromo-4-chloro-3′-indolyphosphate (13 513 022; Roche, Switzerland). Finally, the samples were embedded in 80% glycerol. Stained worms were imaged using an inverse laboratory microscope (DM IL LED; Leica Microsystems, Germany), with image acquisition using LasAF software (Leica Microsystems, Germany).

### RNA isolation, cDNA synthesis and quantitative reverse transcriptase quantitative (real time) PCR analyses

For RNA isolation of worms of experimental and control groups, we first separated couples by incubation in 0.25% (*w/v*) ethyl 3-aminobenzoate methanesulfonate (Sigma–Aldrich, Germany), as described elsewhere (52). Male and female worms were separately collected in 1.5 ml reaction tubes and washed with PBS. The supernatants were discarded, the worms were transferred in 50 μl RNAzol (RNAzol RT Kit; Sigma–Aldrich, Germany), and immediately frozen in liquid nitrogen. For RNA isolation, deep-frozen samples were thawed on ice and processed as suggested by the manufacturer. Elution of RNA was carried out in 20 μl DEPC-treated water. Concentration and integrity of the total RNA were analyzed by electropherogram analysis in combination with the RNA 6000 Nano Kit (2100 Bioanalyzer instrument, Agilent Technologies; USA). The cDNA synthesis of each RNA sample was performed with 100 ng (bF) or 10 ng (sF) total RNA in one reaction using the QuantiTect Reverse Transcription Kit (Qiagen, Hilden, Germany). The final reaction mixture was incubated at 42°C for 60 min, and the reaction stopped at 95°C for 3 min.

Transcript levels were determined by reverse transcriptase quantitative (real time) PCR (RT-qPCR) with primers specific for genes of interest (Supplemental Table S3). In all cases, an exon-spanning primer design was implemented to avoid amplification of remaining genomic DNA (gDNA). Primer efficiencies were determined according to the specificity of (single) PCR products and the absence of primer dimers, as described before (69). Reaction mixtures consisted of 10 μl 2× Quanta mix (Qiagen, Hilden, Germany), 0.8 μl specific primer mix (forward and reverse primer, each 10 μM), 5 μl template cDNA and 4.2 μl PCR-grade water (Carl Roth, Germany). The cycling conditions were as follows, initial denaturation 95°C for 3 min, followed by 55 cycles of DNA denaturation at 95°C for 10 s, primer annealing at 60°C for 15 s and elongation at 72°C for 20 s. The amplification process was followed by a melt curve analysis at 60 to 95°C with stepwise increase of 1°C for 20 s each cycle (Qiagen Rotor-Gene Q, Q-Rex, Qiagen; Germany). Transcript levels of genes of interest were determined by application of the Pfaffl method (70). Sm*letm-1*, a proven RT-qPCR reference gene for *S. mansoni in vitro* studies, was used as control (65,69,71). RT-qPCR was carried out as described applying selected primers (Supplemental Table S4).

### Bioinformatic characterization and phylogenetic classification

Annotations for Smp_144170 (SmRAR) were gathered by collecting data of the transcriptome and genome databases schisto.xyz (24,25,71), parasite.wormbase.org (41), and alphafold.ebi.ac.uk (72,73), respectively. The domain structure of Smp_144170 was predicted by analyzing its amino acid (aa) sequence by SMART (smart.embl-heidelberg.de) (74). The protein structure of SmRAR was predicted by a comparative analysis to already known structures of other NRs using Phyre2 (Protein Homology/analogY Recognition Engine V 2.0) (75), applying the intensive modelling mode.

For the generation of a phylogenetic tree, Smp_144170 orthologs were identified by NCBI protein BLAST (76) and the ortholog finder function of parasite.wormbase.org (41). Orthologs with highest aa similarities were obtained from *Schmidtea mediterranea, S. japonicum, S. haematobium, S. rodhaini, Fasciola hepatica, Opisthorchis viverrini, Dicrocoelium dendriticum, Caenorhabditis elegans, Echinococcus granulosus, Drosophila melanogaster, Xenopus laevis, Homo sapiens* and *Mus musculus* (Supplemental Table S5). Paralogs of *S. mansoni* Smp_144170 were identified in WormBase ParaSite (41). Orthologs from *Stylophora pistillata* and *Acropora millepora* were included as outgroup controls of evolutionary basal organisms (phylum: *Cnidaria*). Alignment was performed by MUSCLE [MUltiple Sequence Comparison by Log-Expectation (77)]. Phylogenetic classification was carried out by MEGA11 (molecular evolutionary genetics analysis) (78) applying the following parameters: the maximum likelihood setting was applied as the statistical method of choice. As test of phylogeny, the Dayhoff model with 1,000 Bootstrap replicates was conducted analyzing the amino acid sequences of certain receptors. As tree inference, the nearest neighbor interchange heuristic method was used (79).

The amino acid sequences of the ligand-binding domains (LBD) and the DNA-binding domains (DBD) of Smp_144170, and its orthologs from *S. rodhaini, S. haematobium* and *S. japonicum*, were determined by SMART (smart.embl-heidelberg.de) (74), and compared by the Clustal Omega – Multiple Sequence Alignment tool (www.ebi.ac.uk/jdispatcher/msa/clustalo) (80,81), respectively.

Potential interaction partners of Smp_144170 were predicted using the STRING Protein–Protein Interaction Network tool (string-db.org) (82,83) on the *S. mansoni* database by using the full STRING network as network type, a medium confidence (0.400) score and a cut-off size of 10 interactions.

Orthologs and known *S. mansoni* genes involved in RA signaling and metabolism (28,84–86), meiosis (17,27,52,87–99) and/or double-stranded DNA (dsDNA) repair (100–103) were compared with already characterized orthologs from vertebrate (92,94,96,97,99,104) and invertebrate (27,28,85,95,98,101–103,105–110) model organisms, and their given annotations checked by uniprot.org and alphafold (72,73) using the implemented BLASTP function of WormBase Parasite (genome version V10) (41,111,112). To verify the presence of essential domains, the protein structures were analyzed using SMART domain analysis (smart.embl-heidelberg.de) (74). The annotations of the identified orthologs were compared with the annotations of the available *S. mansoni* databases (24,25,27,32,111) as well as Kyoto Encyclopedia of Genes and Genomes (KEGG) pathway analysis (113,114), and were compiled in Supplemental Table S6. Finally, cluster-specific transcription of these genes was analyzed using the scRNA-seq atlas of isolated ovaries.

### Statistics

Data received from RT-qPCR analysis were statistically analyzed by comparative testing of the normalized transcription-levels as previously described in Moescheid and Puckelwaldt *et al*. (65). Statistical analyses and tests were carried out using the GraphPad Prism V.8 software (GraphPad Software, San Diego; USA) applying the grouped, two-tailed *t*-test for parametric distributed data or the two-tailed Mann–Whitney test, for non-parametrically distributed data. *P*-values < 0.05 were considered as statistically significant.

## Results

### The mature female ovary contains cells at different developmental stages

**Figure 1.**
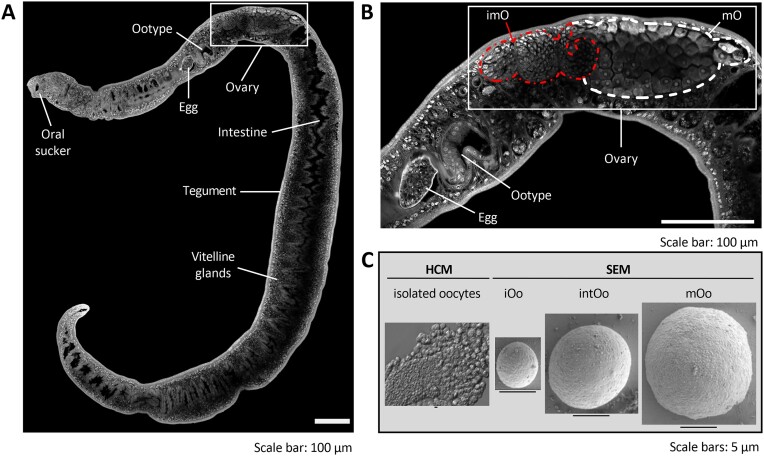
Microscopic analyses displayed different stages of oocyte differentiation. (**A**) Representative picture of a sexually mature *S. mansoni* female, previously separated from a male partner for CLSM analysis. Different organs and tissues are indicated. The position of the ovary is framed. (**B**) Close-up of the framed section in panel (A), showing the detailed structure of the ovary. This organ has a pear-like structure with two separate areas, a smaller anterior part (imO, here left side of the ovary, outlined in red) containing immature oocytes and a larger posterior part (mO, here right side of the ovary, outlined in white) containing mature oocytes. The ootype [marked; here, egg formation occurs (14,45)], as well as an egg within the uterus are indicated. (**C**) Left side: image taken by HCM of oocytes obtained immediately after trypsinization of an ovary extracted from a paired female. Right side: pictures of oocytes subjected to structural analysis by SEM. Oocytes at different developmental stages are shown: immature oocytes (iOo, oogonia, about 5 μm in size), differentiating intermediate-stage oocytes (intOo, 8–12 μm in size) and mature oocytes (mOo, primary oocytes, about 15 μm).

The starting point of this study was the organ isolation approach for adult schistosomes (33,40), which among other tissues allowed us to obtain complete and intact ovaries. Schistosome ovaries are pear-shaped, with a smaller anterior part containing undifferentiated, stem-cell like immature oocytes (iO) and a bigger posterior part containing differentiated mature, primary oocytes that have entered meiosis I (mO). This structure is a common features of trematode ovaries (23,115–117). Isolated ovaries were trypsinized to obtain oocytes in suspension for further analyses. Microscopy analysis confirmed that the approach used to extract ovaries and isolate oocytes delivered cells of good quality for further analyses (Figure 1), as also shown before (26).

### Four distinct cell clusters characterize the ovary of paired *S. mansoni*

**Figure 2.**
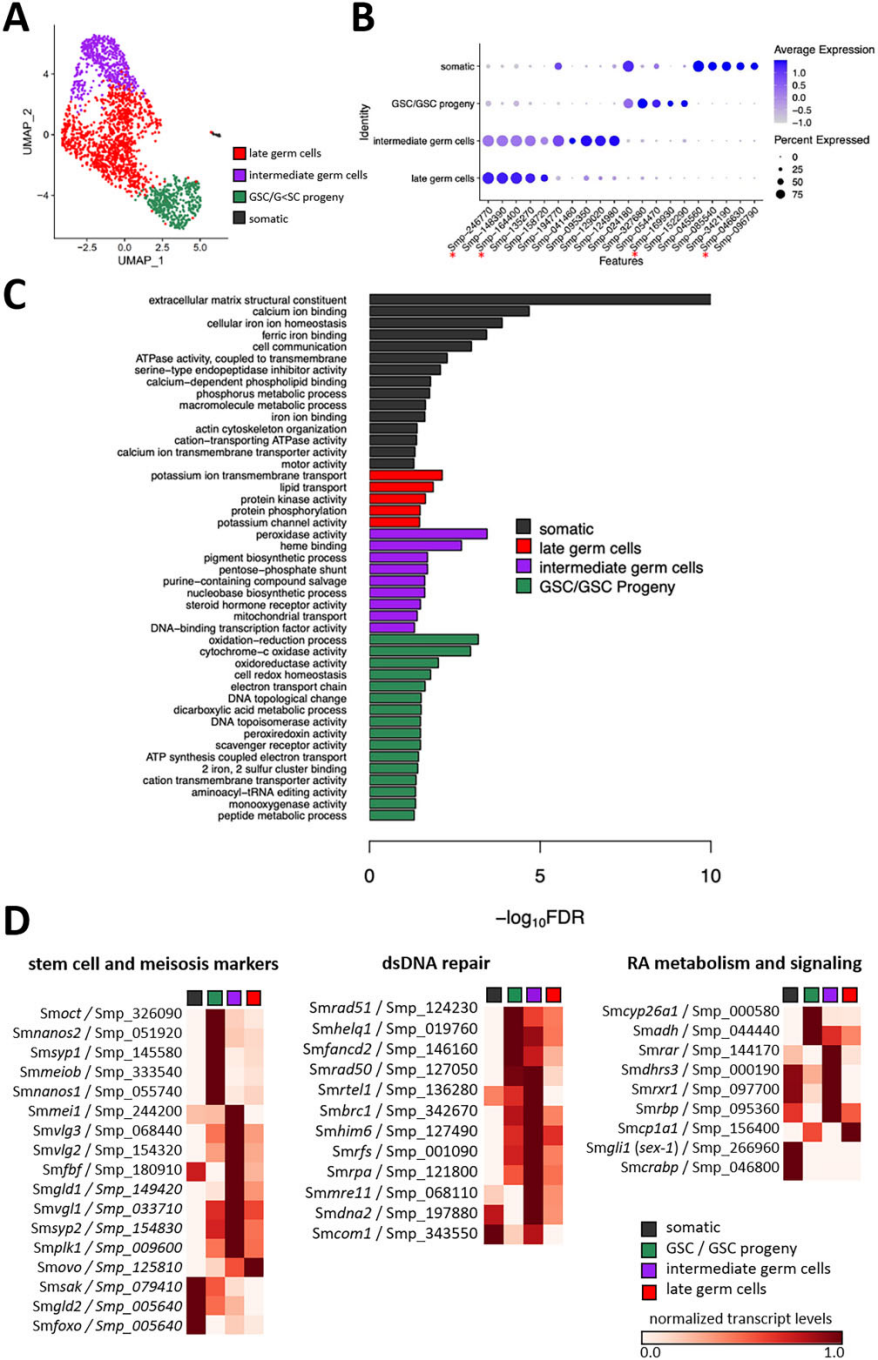
ScRNA-seq identified four distinct oocyte clusters in the ovary of paired females. (**A**) The Uniform Manifold Approximation and Projection (UMAP) representation of 1967 cells in four clusters. (**B**) Dot-plot summarizing the cluster-specific expression of each of the top five marker genes. Asterisks indicate the gene markers previously validated by Wendt *et al*. (27). (**C**) GO terms associated with marker genes of each indicated cluster were annotated with GO terms in the Biological Process and Molecular Function categories with FDR < 0.05. Significant terms are grouped and color-coded by their assigned cluster. (**D**) Heatmap of the oocyte scRNA-seq cluster-associated transcript amounts of genes coding for orthologs of meiosis (17,27,52,87–99), dsDNA repair (100–103) and RA-metabolism/signaling (28,84–86) associated genes. The heatmap illustrates the feature-standardized transcript-levels for each cluster of selected genes.

For scRNA-seq, we isolated 69 intact ovaries from paired females (bO, ovary of a pairing-experienced adult female), which were separated from their male partners after portal perfusion from the final host. Following ovary trypsinization, we obtained cell suspensions with estimated 500 cells/μl for 10X library preparation and sequencing. After mapping and filtering using the Cell Ranger tool (10X Genomics, USA), 2,029 cells were captured from bO with a total of 400 M reads (Supplemental Table S7). The total mapping rate to the v7 *S. mansoni* genome was 91.3%, and the median number of transcriptionally represented genes per cell was 974. Similar to our previous RNA-seq analysis of the complete ovary (24), a total of 8,337 genes were detected across all cells. After further filtering, 1,967 cells expressing 7,872 genes remained for downstream analysis. By unsupervised clustering with Seurat, we categorized four distinct ovarian cell clusters named: ‘late germ cells’ (1,053 cells), ‘intermediate germ cells’ (479 cells), ‘Germ-line Stem Cell (GSC)/GSC progeny’ (418 cells) and ‘somatic cells’ (17 cells) (Figure 2A and Supplemental Table S8). We identified marker genes for each of the cell clusters using the Wilcoxon Rank Sum test (Figure 2B). The identities of the clusters were consistent with previous findings (27), with a few genes already validated by earlier WISH experiments, and markers of ‘somatic’ cells expressed in multiple clusters, including muscles, parenchyma and neurons (Supplemental Table S9). Enriched GO terms also supported the cluster assignations (Figure 2C). In somatic cells, we found marker genes associated among others with calcium- and free iron-binding, cell communication and muscle activity (e.g. motor activity and actin cytoskeleton organization; Supplemental Table S10). In the GSC/GSC progeny cluster, GO terms of oxidation/reduction processes and electron transport activity dominated (118–121), whereas ‘intermediate stage’ characteristics were GO terms enriched for peroxidase activity and heme binding, as well as steroid hormone receptor activity. In ‘late germ cells’ potassium ion and lipid transport as well as kinase activities were significantly enriched (FDR < 0.05) (122). Moreover, orthologs of important stem cell and meiosis marker genes, such as Sm*nanos1* (Smp_055740) (27,52,89) and Sm*nanos2* (Smp_051920) (27,52,123), the VASA-like RNA-helicases Sm*vlg1-3* (Smp_033710, Smp_154320, Smp_068440) (88,108) and orthologs of the SYP family such as SYP-2 (Sm*syp2*, Smp_154830) (99), a key regulator of meiotic prophase in *C. elegans* (124,125), were found to be transcribed in the GSC/GSC progeny and the intermediate germ cell cluster, respectively (Figure 2D and Supplemental Table S6). In addition, orthologs of *C. elegans* factors essential for dsDNA break repair such as RAD51 (Sm*rad51*, Smp_124230) (101,102), and RA-signaling such as the cellular retinol-binding protein Rbp (Sm*rbp*, Smp_095360) (85,126) were found to be transcribed in cells of the GSC/GSC progeny cluster (Figure 2D), emphasizing an influence of RA-signaling on schistosome reproduction.

### A novel, pairing-regulated RAR-like NR occurred among top marker genes in intermediate germ cells

To identify genes mediating pairing-dependent oocyte maturation, we focused on genes within the ‘intermediate germ cells’ cluster. Here, we found 14 genes that were not yet assigned as cell markers in previous single cell studies in schistosomes (27) (Supplemental Table S9). By exploring gene expression data across different developmental stages (25), these genes exhibited almost exclusive expression in the ovary (Supplemental Figure S2). Based on their annotations and associated KEGG pathways (113,114,127), we next selected those genes showing potential involvement in cell cycle, metabolic regulation, or receptor signaling and combined this information with bO-preferential expression (24), resulting in eight candidate genes with putative roles in ovary maturation: Smp_332250 (G protein-coupled receptor), Smp_018010 (Cytospin-A), Smp_146810 (diaphanous), Smp_314200 (Dyp-type peroxidase), Smp_321620 (threonine tRNA ligase), Smp_129520 (GRIP domain-containing protein), Smp_145400 (dynein heavy chain) and Smp_144170 (RAR-like NR). The latter gene was further characterized, given its potential function as TF regulating cell development (113,114,128), and already described roles of other RARs in schistosome reproduction (21,35,129–132).

**Figure 3.**
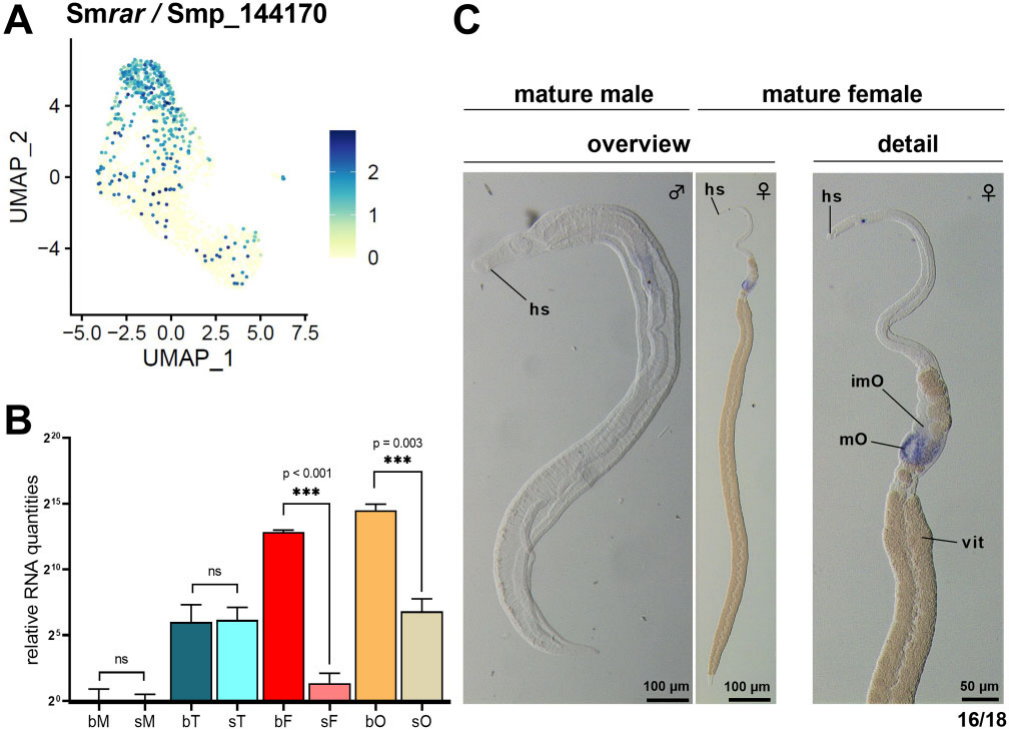
Sm*rar* expression in ovaries is pairing-dependent and localized in the posterior part of the ovary. (**A**) UMAP-visualization of clustered scRNA-seq data from oocytes, colored by Sm*rar* (Smp_144170) transcript level, showed expression primarily in oocytes of an intermediate developmental stage, along with some additional expression in mature oocytes. (**B**) RT-qPCR analysis of Sm*rar* transcripts in pairing-experienced and -unexperienced adult schistosomes and their gonads demonstrated its ovary-preferential and pairing-dependent transcript pattern. *P < 0.05, **P < 0.01, ***P < 0.001 by *t*-test; bM, bisex male (pairing-experienced male); sM, single-sex male (pairing-unexperienced male); bT, testes of bM; sT, testes of sM; bF, bisex female (pairing-experienced female); sF, single-sex female (pairing-unexperienced female); bO, ovary of bF; sO, ovary of sF. (**C**) Whole-mount *in situ* hybridization revealed the specific localization of Sm*rar* transcripts in the posterior part of the ovary of paired female *S. mansoni* (right), which was found in the majority of the worms examined (16 out of 18). No specific signals occurred in male schistosomes (left). hs, head sucker; imO, part of the ovary containing immature oocytes; mO, part of the ovary containing mature oocytes; vit, vitellarium.

Across our clustered scRNA-seq data, Smp_144170 showed expression in oocytes representing the intermediate developmental stage, and in mature oocytes (Figure 3A). Smp_144170 was also among the marker genes of the intermediate-stage oocyte cluster (Supplemental Table S9) with expression in >60% cells. Previous bulk transcriptome data indicated an ovary-preferential as well as pairing-dependent transcript pattern of Smp_144170 (24,25). For confirmation, we performed RT-qPCR analyses with cDNA of pairing-experienced (bM, bF) and pairing-unexperienced (sM, sF) males and females, respectively, and their isolated gonads (bT, bO, sT, sO). The results confirmed the ovary-preferential and pairing-dependent expression of Smp_144170 (Figure 3B), consistent with the available transcriptional profile data similar to that of the previous transcriptome data (24,25). Next, we performed WISH to localize Smp_144170 transcripts in mature females and males using a 500 bp long, single-stranded riboprobe. We detected specific signals in the posterior part of the ovary, which contains differentiated oocytes (Figure 3C). No signals were evident in the anterior part of the ovary, where oogonia, the immature, stem cell-like oocytes are located (Figure 1). Similarly, no signals occurred in males. These findings aligned with our expectation of a gene primarily expressed in the ovary. Furthermore, the higher signal intensity at the edges of the posterior part of the ovary (and less signal intensity in the middle of the anterior part of the ovary) correlated with the clustering results from scRNA-seq, indicating a preferential expression in intermediate-stage oocytes. Fully matured oocytes fill the center of the posterior part of an ovary of paired females (23,59), where we found lower-intensity signals.

**Figure 4.**
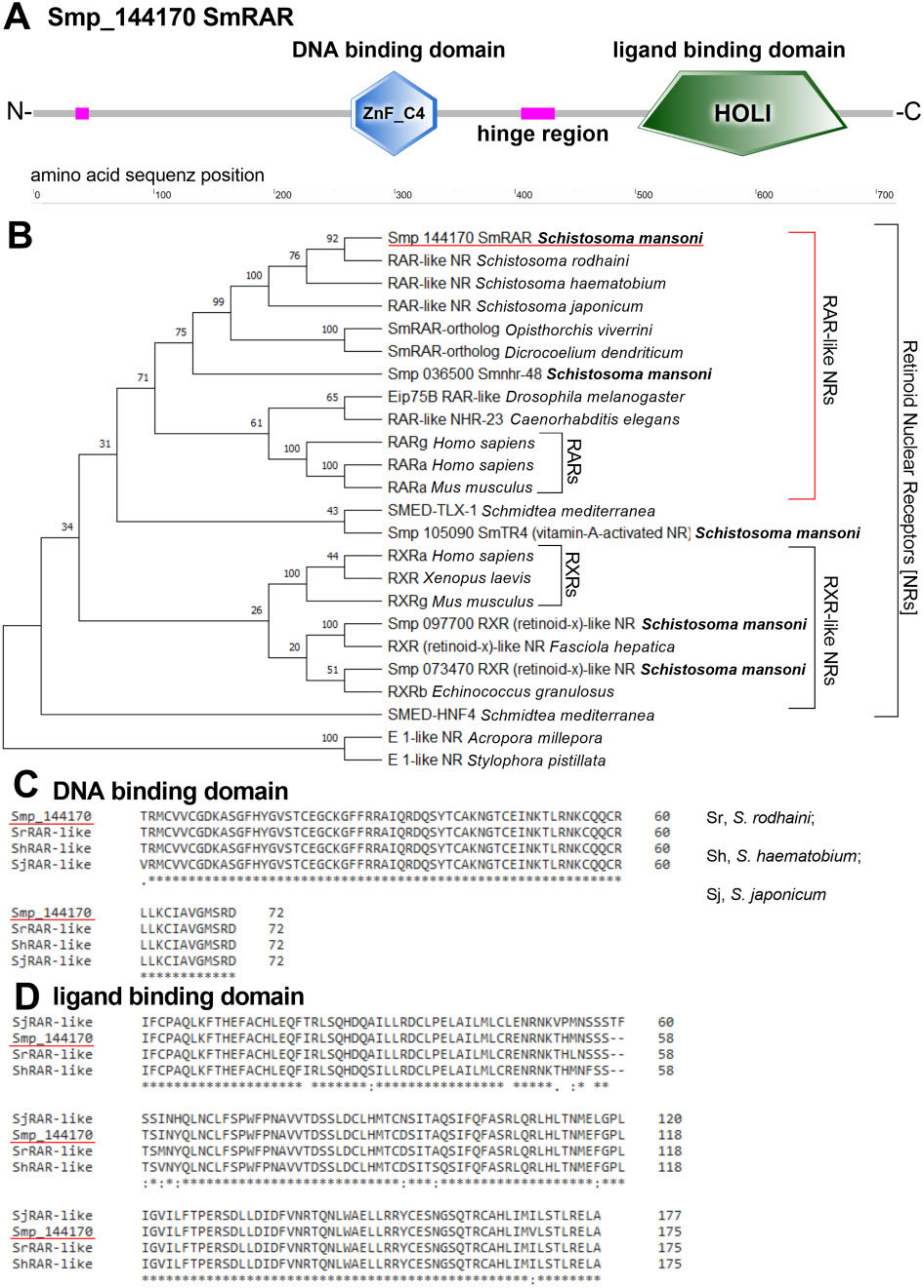
Bioinformatic and phylogenetic analyses revealed SmRAR as a NR of the retinoid receptor family. (**A**) The domain structure of Smp_144170 (SmRAR). SMART domain analysis (74) of SmRAR revealed the typical NR structure consisting of unstructured N- and C-terminal domains, as well as a zinc finger domain (ZnF_C4), which is linked by a hinge region to the LBD (HOLI). (**B**) Phylogenetic analysis, using MEGA11 (78), positioned SmRAR within the RAR-like clade of NRs. The aa sequence of SmRAR was compared with paralogs and orthologs of other species. Orthologs of *A. millepora* and *S. pistillata*, representing the evolutionary basal stem of *Cnidaria*, served as outgroup references. (**C, D**) Shown are multiple sequence alignments of the (**C**) DBDs and (**D**) LBDs of SmRAR and other RAR-like proteins, obtained by Clustal Omega (80,81). RAR-like orthologs of different schistosome species were used (*S. rodhaini*, Sr; *S. haematobium*, Sh; and *S. japonicum*, Sj). ZnF, zinc finger domain.

To unravel the identity of Smp_144170, we performed a reanalysis of its predicted annotation as a member of the NR subfamily 1 (24,25), or RAR (retinoic acid receptor)-like NRs (41,72,73). SMART domain analysis (74) of the aa sequence of SmRAR indicated a NR-typical domain structure consisting of variable N- and C-terminal regions and a zinc-finger domain connected to a LBD by a hinge region (Figure 4A). Comparative structural analysis of the predicted protein structure by Phyre2 (75) showed highest similarities to the RAR beta subfamily (RARb, Supplemental Figure S3). Furthermore, phylogenetic analyses (78) using the aa sequences of SmRAR paralogs and orthologs of other organisms with different evolutionary distances revealed the evolutionary relationship of SmRAR to other known RARs (Figure 4B). The results showed a clustering of RAR-like NRs of trematodes separate from other invertebrates as well as vertebrates. This suggests the existence of a trematode-specific subgroup within the RAR-like NRs. Moreover, gene duplication within the family of the RAR-like receptors within the trematodes was evident.

Comparison of LBD and DBD showed high conservation of both domains among different members of the Schistosomatidae (*S. mansoni*, S*. rodhaini, S. haematobium* and *S. japonicum*), with only a low number of single aa deviations (Figure 4C and D). These findings perfectly correlate with a previous phylogenetic analysis of schistosome species, which exhibited an African clade with *S. rodhaini* as closest relative of *S. mansoni*, and *S. japonicum* as one of the most distantly related members representing the Asian clade (133). Furthermore, SmRAR showed a different evolutionary classification compared to other *S. mansoni* internal RA receptors: Smp_097700 and Smp_073470, which were shown to be potential dimerization or interaction partners of SmRAR (21,129,131). SMART domain analyses of both paralogs verified their identities as NRs; however, they were classified as members of the retinoid-x-NR (RXR) family (41,74). As an ortholog of SmRAR we found a RXR (THD22775) of *F. hepatica*. Phylogenetic analysis of this RXR revealed one closely related RXR of *S. mansoni* (Smp_097700) and a more unrelated second RXR (Smp_073470; Figure 4A). In contrast, SmRAR appeared more closely related to RAR-like orthologs from other trematodes like *O. viverrini* and *D. dendriticum* than to orthologs of the nematode *C. elegans* (RAR-like NHR-23) or *D. melanogaster*, mice and human.

### Functional characterization indicates a major role for SmRAR in the maintenance of mature oocytes

To get first insights into the biological function of SmRAR, we performed gene-specific knock-down (KD) experiments using RNAi. To avoid RNAi-dependent off-target effects on other NRs, the dsRNA was designed to be Sm*rar*-specific without covering the highly conserved DBD and LBD. To minimize dsRNA-dependent off-target effects on the transcription of genes analyzed by RT-qPCR, small interfering RNA (siRNA)-finder (si-fi) software for dsRNA off-target prediction (134) was applied as previously described (65). Additionally, the whole length dsRNA sequence was compared to the mRNA sequences of selected genes using the BLASTn algorithm (112). For these genes, which we selected for further downstream analysis, no significant sequence similarities of sufficient length provoking RNAi off-target effects (134) were found. Furthermore, primers for downstream analysis by RT-qPCR were designed exon-spanning by avoiding any overlap with the dsRNA sequence.

**Figure 5.**
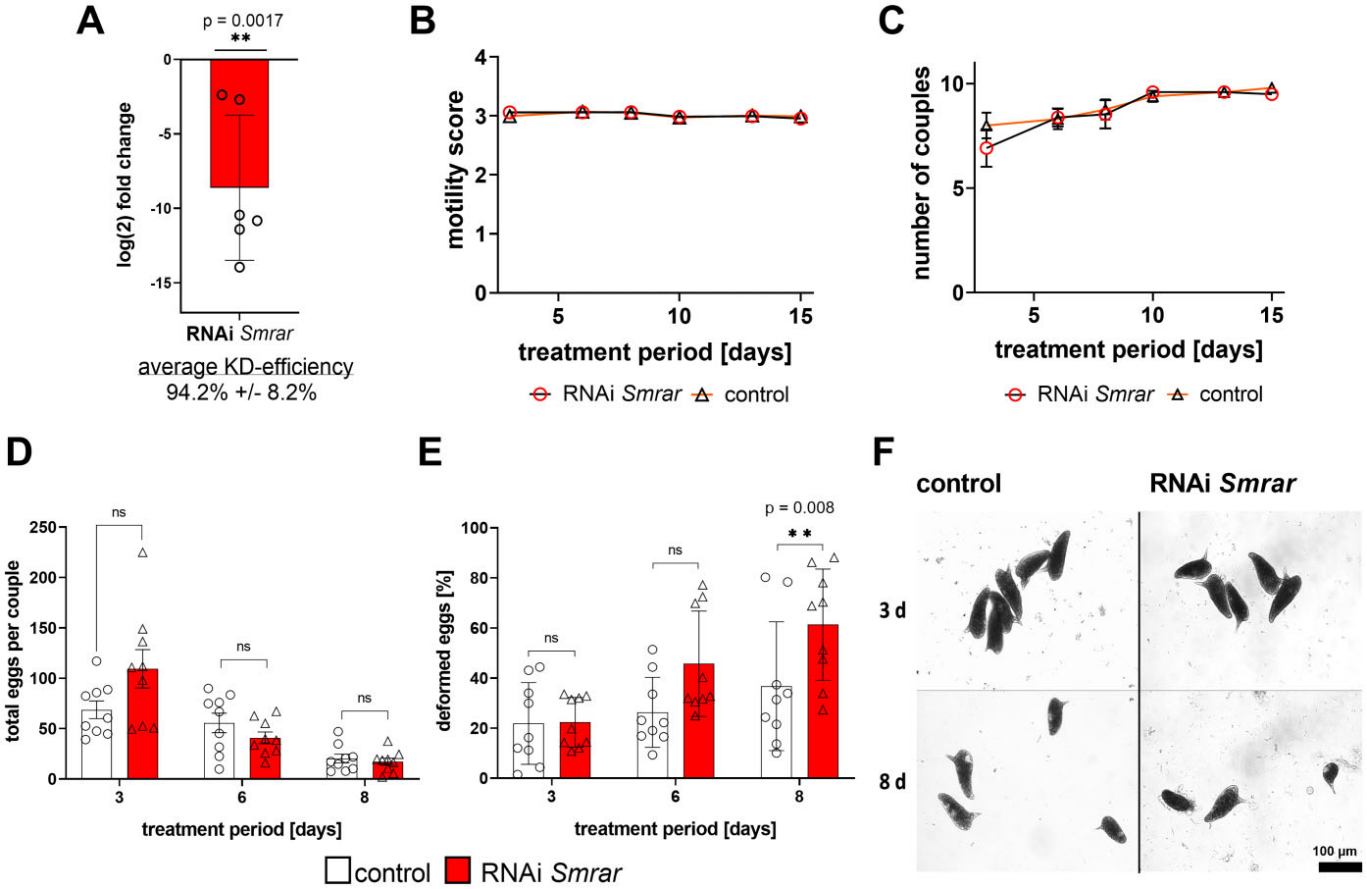
Sm*rar* RNAi affected egg morphology and embryo development. Schistosome couples were treated with 30 μg ml^−1^ Sm*rar* dsRNA, every 2 to 3 days for a period of 15 days (red bars), or without dsRNA (control; white bars) under the same conditions. (**A**) KD efficiency was evaluated using RT-qPCR to measure transcript levels of Sm*rar* in female worms separated from couples. Phenotypes were measured as follows: (**B**) Worm motility, scored from 0 (no motility) to 4 (hyperactive motility), showed no change between experimental groups; (**C**) Pairing stability showed no change between experimental groups (triangles and circles represent the biological replicates, *n* = 9); (**D**) The number of eggs produced *in vitro*, monitored over an 8-day period, showed no significant differences after RNAi treatment; (**E**) 8 days of RNAi induced a significant increase in malformed, smaller and non-embryonated eggs, often without spines or deformed spines; (**F**) The morphology of eggs after 3 and 8 days was analyzed by bright-field microscopy. After 8 days, egg morphology altered, and significantly, more eggs were reduced in size. **P < 0.01, determined by *t*-test. A, *n* = 6; B-F, *n* = 9.

The RNAi experiment was performed with 10 couples and 9 biological replicates in total. Couples treated the same way but without dsRNA served as control. Additionally, previous studies with non schistosome-related control dsRNA showed no obvious phenotypes at concentrations of 30−60 μg/ml (65,66). The Sm*rar* KD efficiency was determined by RT-qPCR after 15 days. For this, couples were first separated, and then RNA extracted from the females and used for RT-qPCR, which confirmed a significant KD of Sm*rar* at the mRNA level (94.2 ± 8.2% reduction compared to the control) (Figure 5A).

**Figure 6.**
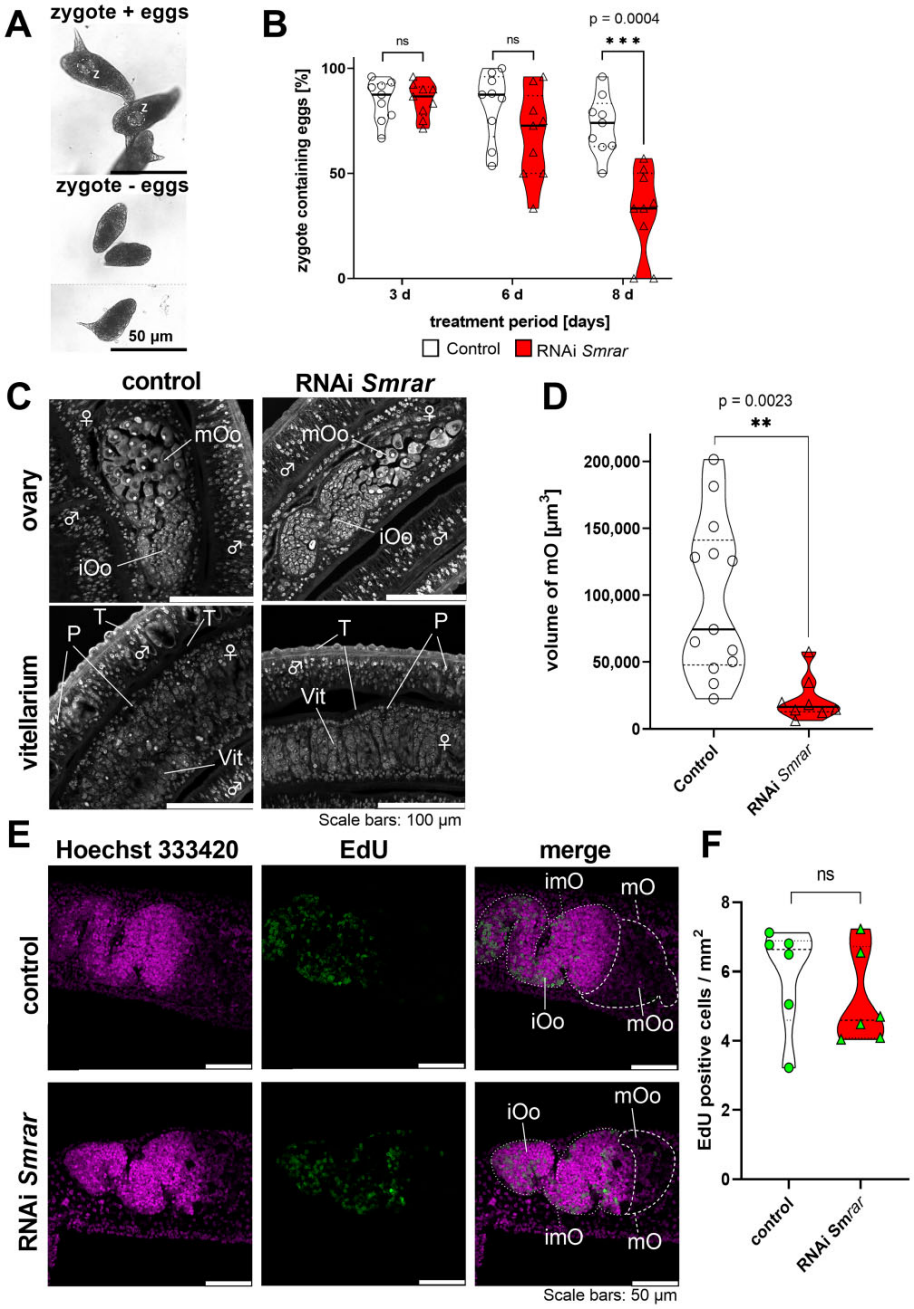
Sm*rar* KD resulted in a drastic reduction of the number of zygote-containing eggs and oocyte deficiency. Schistosome couples were treated with 30 μg ml^−1^ Sm*rar* dsRNA for 15 days. (**A**) The morphology of eggs produced *in vitro*, using bright-field microscopy. (**B**) Weighted distribution (violin plot) of zygote-containing eggs upon dsRNA treatment. After 8 days, a significant reduction in zygote-containing eggs was observed for dsRNA-treated couples (red) compared to controls (white). (**C**) CLSM analysis of carmine red-stained, paired worms showed a reduction of the size of the mature ovary of dsRNA-treated couples. No morphological changes were observed in the vitellarium of treated females or in the 0parenchyma and tegument of either sex. (**D**) Violin plot of the distribution in volumes of the posterior part of the ovaries based on comparative Z-stack analysis of carmine-red stained females. The ovaries from females of six biological replicates were examined; each point represents the volume of a single ovary. A significant reduction in the volume was seen in the RNAi group. (**E**) CLSM analysis of EdU-treated female schistosomes showed a similar abundance of stained cells in the control and RNAi groups, and signals (green) occurred in the anterior part of the ovary, which contains immature oocytes (oogonia). The dimensions of these ovary sections were similar in both experimental groups, whereas the dimensions of the posterior section of the ovaries, containing mature oocytes, were clearly reduced in females of the RNAi group. Cell nuclei were counter-stained with Hoechst 33 342 (purple). (**F**) Violin plot of the distribution of proliferating cells in the ovary in the plane of maximum extension. No RNAi-dependent effects on cell proliferation were observed. In the violin plots, individual values of biological replicates are shown as well as their median values (solid lines). Abbreviations: iOo, oogonia; imO, immature part of the ovary; mOo, mature oocyte; mO, mature part of the ovary; P, parenchyma; T, tegument; Vit, vitelline lobe; z, zygote. *P < 0.05, **P < 0.01, ***P < 0.001 by *t*-test. A, *n* = 9; B-D, *n* = 6.

For all experimental groups, the worms were monitored daily to determine physiological and morphological effects using a standardized scoring system (65). During the whole experimental period, worms of all groups were viable, and there was no decrease in motility or pairing stability (Figure 5B and C). As physiological read-out, the percentages of normal and malformed eggs per couple were determined during the first 8 days of treatment. There was no significant difference in the total number of laid eggs between the RNAi and the control groups (Figure 5D). After 8 days, however, a significant increase of malformed eggs was observed only in the RNAi group (Figure 5E). In contrast to eggs of the control group, eggs of the treatment group were reduced in size, spines were occasionally absent or deformed, and embryos were absent in these eggs (Figure 5F). Furthermore, after 8 days, the number of zygote-containing eggs was significantly reduced in the RNAi group (68.4 ± 19.5%; *P* < 0.001) compared to the control (Figure 6A and B).

Given the prior observation suggesting a potential effect stemming from the female gonad, especially the ovary, we investigated the intricate structures of ovaries in females from both the RNAi and control groups after 15 days using CLSM (Figure 6C and D). In addition, EdU staining was performed to investigate potential effects on germinal stem cell proliferation (Figure 6E and F). By CLSM, a significant reduction in the number of mature oocytes was observed in the RNAi group only as well as a significant reduction of the ovary volume. The latter was determined by Z-stack analyses (Figure 6C and D). The reduced ovary volume coincided with the smaller size of the posterior part of the ovary that we repeatedly detected in females of the RNAi group (Figure 6C and D). In addition to an untreated control (no dsRNA), we used an irrelevant dsRNA, *ampR* (ampicillin resistance gene of *E. coli*), previously identified as a well-suited reference for *in vitro* RNAi experiments with *S. mansoni* (65). No effects on the morphology and the volume of the mature ovary were observed (Supplemental Figure S4). Next, results of the EdU assay showed no difference in the number of EdU-positive, proliferating oogonia between the control and the RNAi groups (Figure 6E and F). Furthermore, we observed no RNAi effects on the vitellarium (Figure 6C). In addition, investigation of the testes of males derived from dsRNA-treated couples showed no alteration in their morphology, the size of the testicular lobes, or the seminal vesicles (Supplemental Figure S5A). In addition, the number of EdU-positive spermatogonia was similar between the two experimental groups (Supplemental Figure S5B).

### Sm*rar* RNAi associated with downregulation of meiosis-associated genes

**Figure 7.**
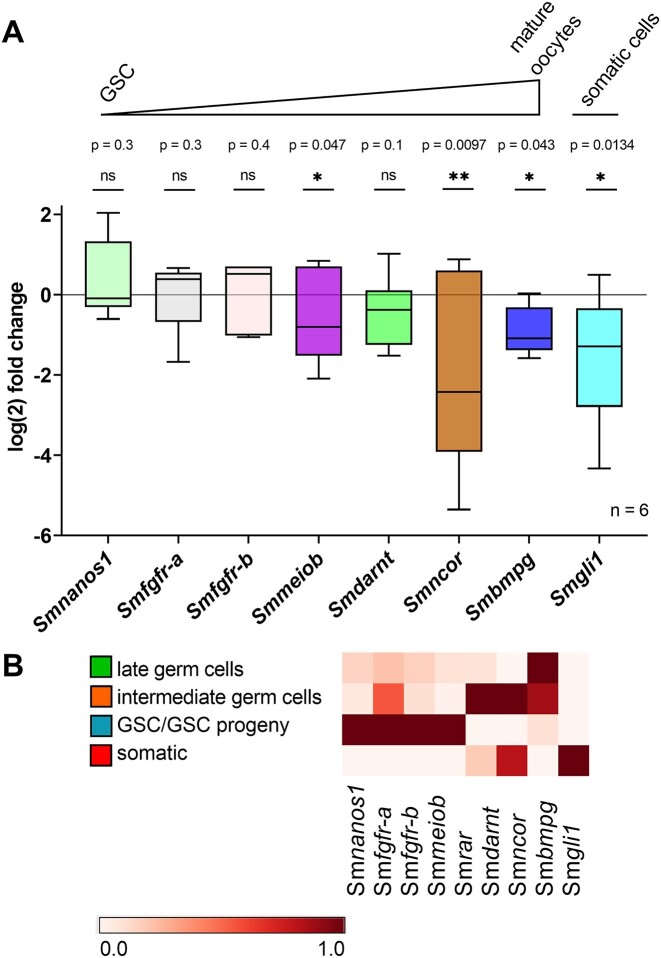
The meiosis-associated genes Sm*ncor*, Sm*meiob* and Sm*gli1* were significantly downregulated after Sm*rar* RNAi. (**A**) Schistosome couples were treated with 30 μg ml^−1^ Sm*rar* dsRNA for 15 days, then females separated and RNA isolated. Subsequently, transcript levels of candidate genes were quantified by RT-qPCR (*n* = 6). Candidate genes, as indicated, were interacting partners of Sm*rar* predicted by STRING network analysis (82,83), or they had been shown previously to be associated with different stages of ovary development (27,53,135). Analyses by RT-qPCR showed significant reductions only of the transcript levels of Smp_163290 (Sm*ncor*, brown), Smp_333540 (Sm*meiob*, purple), Smp_ 078720 (Sm*bmpg*, blue) and Smp_266960 (Sm*gli1*, cyan). In contrast, the transcript levels of Sm*nanos1* (Smp_055740), Smfgfr-a*/b*, (a, Smp_175590; b, Smp_157300) and Sm*darnt* (Smp_123420) appeared unaffected. The transcriptional changes of the analyzed genes were plotted following the oocyte developmental trajectory; from immature, undifferentiated GSCs to mature oocytes. They were arranged according to their transcription peak of the respective cluster of the scRNA-seq atlas of mature ovaries. The boxplot indicates the range between minimum and maximum values, with the 10th and 90th percentiles and the median shown in the box. *P < 0.05, **P < 0.01, ***P < 0.001 by *t*-test. (**B**) Heatmap of the ovary scRNA-seq cluster-associated transcript levels of Sm*rar* and representative cluster genes, which were analyzed by RT-qPCR. The heatmap illustrates the feature-standardized transcript levels for each cluster of selected genes.

To investigate Sm*rar* RNAi-dependent effects on gene expression, we next analyzed the transcript profiles of various genes: (i) those representing potential SmRAR interaction partners, (ii) genes preferentially transcribed in the ovary, and (iii) genes already known to play roles in schistosome reproduction. These genes were selected based on their transcription patterns shown in previous RNA-seq studies, similar to Sm*rar* (24,25). Furthermore, we selected already described orthologs of ovary marker genes in *S. mediterranea* and *S. mansoni* (27,135) (Supplemental Tables S4, S10). Potential interaction partners of SmRAR were predicted by STRING analysis (82,83) (Supplemental Table S3). The transcriptional changes of selected genes were determined by RT-qPCRs and in Figure 7A arranged according to the oocyte developmental trajectory. Beyond that, the relative transcript abundance of these genes within oocyte development was determined using the ovarian scRNA-seq atlas. In particular, genes abundantly transcribed in the GSC/GSC progeny cluster were deciphered (Figure 7B and Supplemental Figure S6).

Among the genes investigated by RT-qPCR were Smp_123420 [putative aryl hydrocarbon receptor nuclear translocator homolog, Sm*darnt*; (82,83)], Sm*nanos1* (Smp_055740) (27,87,123,136), Sm*fgfr*-A (Smp_175590) and Sm*fgfr*-B (Smp_157300) (53). For these four genes, no significant changes in the transcription levels between RNAi against Sm*rar* and control groups were observed. Furthermore, for Sm*nanos1*, Sm*fgfr-a* and *–b* regulatory roles in the early oocytes (oogonia), upstream of Sm*rar* were described in previous studies (53,123,136). These findings are supported by our RT-qPCR analysis (Figure 7A and B) and the localization of Sm*nanos1* transcripts in the anterior part of the ovary (containing oogonia) by WISH (Supplemental Figure S7A) (27). Furthermore, the successful KD of Sm*nanos1* by RNAi (Supplemental Figure S7B) did not affect worm vitality (Supplemental Figure S7C and D), but provoked egg phenotypes similar to those observed in the course of the Sm*rar* RNAi (Supplemental Figure S7E–G), characterized by a significant reduction of zygote-containing eggs upon RNAi (Supplemental Figure S8A). Moreover, the number of both proliferating oogonia and primary oocytes was significantly decreased by Sm*nanos1* RNAi (Supplemental Figure S8B–F), whereby the part of the ovary containing oogonia was considerably more reduced than the part containing mature oocytes (Supplemental Figure S8C and D). These effects were expected since for NANOS and its orthologs a fundamental role in the regulation of stem cell proliferation, such as early oogonia, has been described (94,123,136). In contrast, Smp_333540 [meiosis-specific OB domain-containing protein, SmMEIOB (25)], a known GSC progeny marker identified in EdU-negative germ cells before (27) (Supplemental Figure S9), showed a significant transcript level reduction upon RNAi against Sm*rar* (Supplemental Figure S10).

Furthermore, a significant decrease in the transcript level of Smp_078720 [bone marrow proteoglycan homolog, SmBmpg (25,27)] was observed. Sm*bmpg* has been identified as a marker gene for mature oocytes in the whole worm scRNA-seq atlas of adult schistosomes (27) (Supplemental Figure S9). Another potential SmRAR interaction partner predicted by STRING was SmGli1 [Smp_266960, transcriptional activator glioma-associated oncogene (28)], which was shown before to play a key role in the initiation of female maturation following pairing (28). Upon RNAi against Sm*rar*, a reduction of Sm*gli1* transcripts was observed. The strongest reduction of the transcript level was discovered for Sm*ncor* [Smp_163290, a NR co-repressor related protein, SmNCoR (82)]. This gene is also known as thyroid-hormone- and retinoic acid receptor-associated co-repressor (TRAC) and involved in complexes with histone acetyl transferases (HATs) and chromatin remodeling (137,138). To additionally investigate a stem-cell marker effect, we performed Sm*nanos1* RNAi, which led to a significant reduction in the transcript levels of Sm*meiob* and Sm*bmpg* and a strong trend toward a reduction in the transcript levels of Sm*ncor* (Supplemental Figure S10). This result substantiates the proposed female germline lineage (27) and the transcription patterns obtained in this study (Supplemental Figure S9). Transcript-levels of Sm*gli1* were unaffected by RNAi, which was expected since Sm*nanos1* KD did not significantly affect Sm*rar* transcription, and STRING analysis predicted no SmNanos1 - SmGli1 interaction (82,83).

### Gene KD of Sm*rar* impairs oocyte maturation

Next, we combined RNAi and pairing experiments using pairing-unexperienced, sexually undifferentiated female schistosomes (sF) to investigate the role of SmRAR in ovary development and oocyte maturation upon first-time pairing. In three biological replicates, we treated 15 sF each with Sm*rar* dsRNA for a 7-days period. Subsequently, sFs were (first time) paired with 10 pairing-experienced males (bM) as described before (Li *et al*. 2023). Pairing occurred within 48–72 h, and unpaired males were discarded after 72 h. Couples were cultivated and monitored for additional 13 days, while medium replacement occurred every 2–3 days, along with the addition of fresh dsRNA.

**Figure 8.**
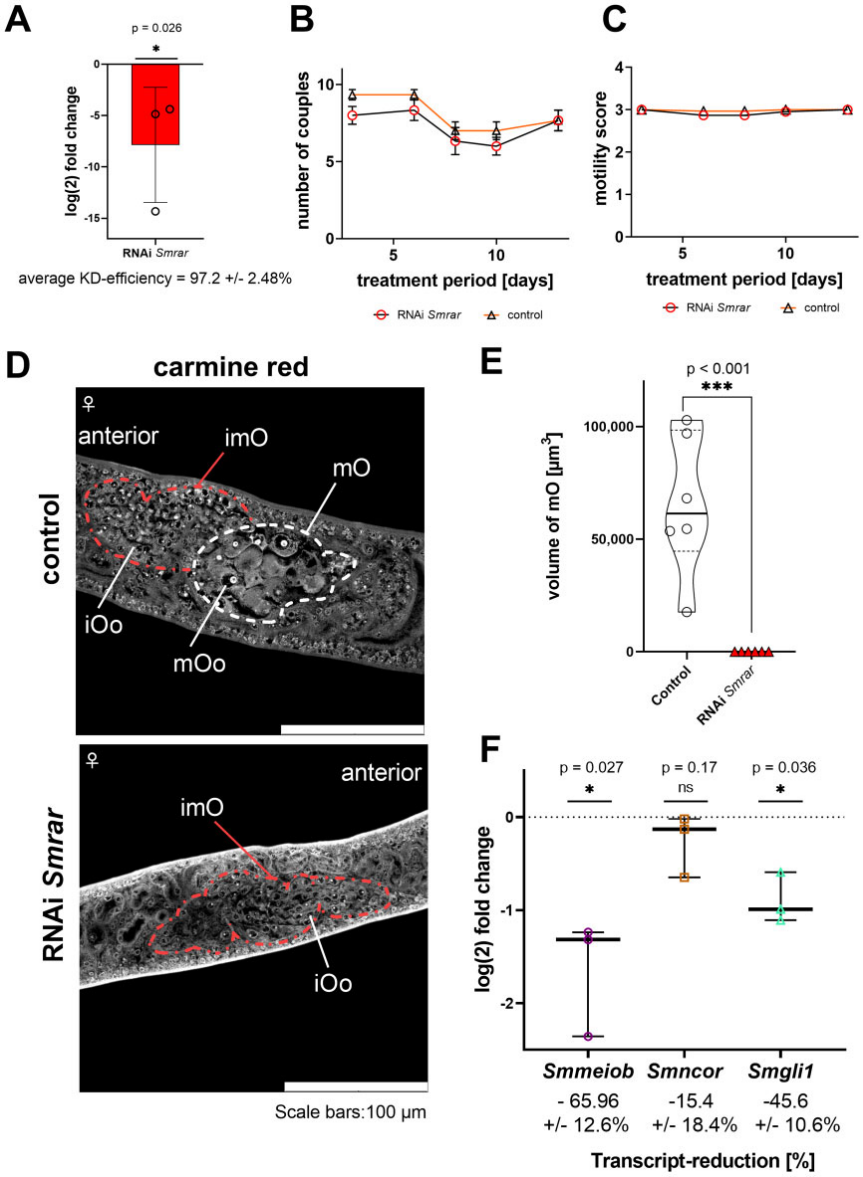
In first-time paired females, Sm*rar* RNAi prevented oocyte maturation and significantly reduced the transcript levels of Sm*meiob* and Sm*gli1*. Sm*rar* RNAi experiments were conducted with pairing-unexperienced female schistosomes (sF) treated with 30 μg dsRNA ml^−1^ for 20 days, split into a pre-pairing and a post-pairing treatment period (*n* = 3). As control, we used untreated worms, and worms treated with an irrelevant dsRNA, *ampR* (ampicillin resistance gene of *E. coli*) (65) (Supplemental Figure S11). After 7 days in culture with dsRNA (added every second day, thus three times in the pre-pairing period), sFs were paired (first-time pairing) with pairing-experienced males (bM) according to a previously published *in vitro*-pairing protocol (44). Couples formed between 48–72 h and were maintained *in vitro* for another 20 days (continued dsRNA addition every second day). (**A**) log_2_fold change in Sm*rar* transcript levels, determined by RT-qPCR, showed a significant reduction in females after 20 days. Individual biological replicates, mean and standard deviation are shown. As physiological parameters, pairing stability (**B**) and motility (**C**) [motility scores: 0 (no motility) to 4 (hyperactive motility)] were monitored but no differences observed between the experimental groups. (**D**) CLSM analysis of females focusing on the ovary showed immature oocytes, oogonia, (iOo; within the red-framed part of the ovary) and mature oocytes (mOo; within the white framed section) but the absence of mature oocytes in ovaries of the RNAi group. (**E**) Corresponding to the oocyte phenotype presented in (**D**) comparative Z-stack CLSM analyses revealed a significant reduction of the volume of ovaries of females of the RNAi group. The violin plot indicates the range between the minimum and maximum values and the median visualized by the solid line. Here, the volume of the posterior part of the ovary, which contains mature oocytes, was determined. The ovaries of females of three biological replicates were examined, whereby each point represents the volume of a single ovary. (**F**) RT-qPCR analysis demonstrated the significant reduction of Sm*meiob* (purple) and Sm*gli1* (cyan) transcript levels in the RNAi group, while a trend of reduction was observed for Sm*ncor*. The plot indicates the range between the maximum and minimum as well as the median. Each individual dot represents the log_2_fold change in transcription compared to the control for each biological replicate. Abbreviations: iOo, oogonia; imO, immature part of the ovary; mOo, mature oocyte; mO, mature part of the ovary. *P < 0.05, **P < 0.01, ***P < 0.001 by Mann–Whitney test.

At the end of the experiment, couples were separated, and RNAi efficiency determined for females by RT-qPCR. KD was successful with a significant reduction of the Sm*rar* mRNA level (97.2 ± 2.48%, Figure 8A). Physiological parameters such as pairing stability and motility were unaffected in the RNAi group and the control (Figure 8B and C). In addition to an untreated control (no dsRNA), we used the irrelevant *ampR* dsRNA (65), which also had no effect on worm viability and morphology (Supplemental Figure S11). We again investigated the RNAi effect at the CLSM level and observed clear morphological differences between the RNAi and control groups (Figure 8D and Supplemental Figure S11). The ovaries of control worms of both control groups showed the typical developmental status with oogonia in the anterior part of the ovary and mature oocytes in its posterior part, which was expected as a consequence of pairing-induced sexual maturation (26,44). In first-time paired females of the RNAi group, however, the situation was different. Although the ovary as such developed, there was a complete absence of differentiated oocytes, with only oogonia present, suggesting a meiotic arrest at the transition from oogonia to the oocyte stage. In these females, the ovary volume was also significantly reduced, an expected consequence of missing mature oocytes (Figure 8E). Additionally, Sm*rar* RNAi also affected egg-morphology and zygote occurrence (Supplemental Figure S12).

Next, we investigated the RNAi effect on the transcript levels of those genes that were shown before to be significantly reduced upon Sm*rar* RNAi, and for which we assumed that they are involved in oocyte development (Figure 8). RT-qPCR analyses showed again a clear reduction of the transcript levels of Sm*meiob* (65.96 ± 12.6%) and Sm*gli1* (45.6 ± 10.6%). This time, we only found a trend of transcript level reduction for Sm*ncor* (Figure 8F).

### Functional characterization of Sm*meiob* by RNAi revealed a Sm*rar* RNAi phenocopy

**Figure 9.**
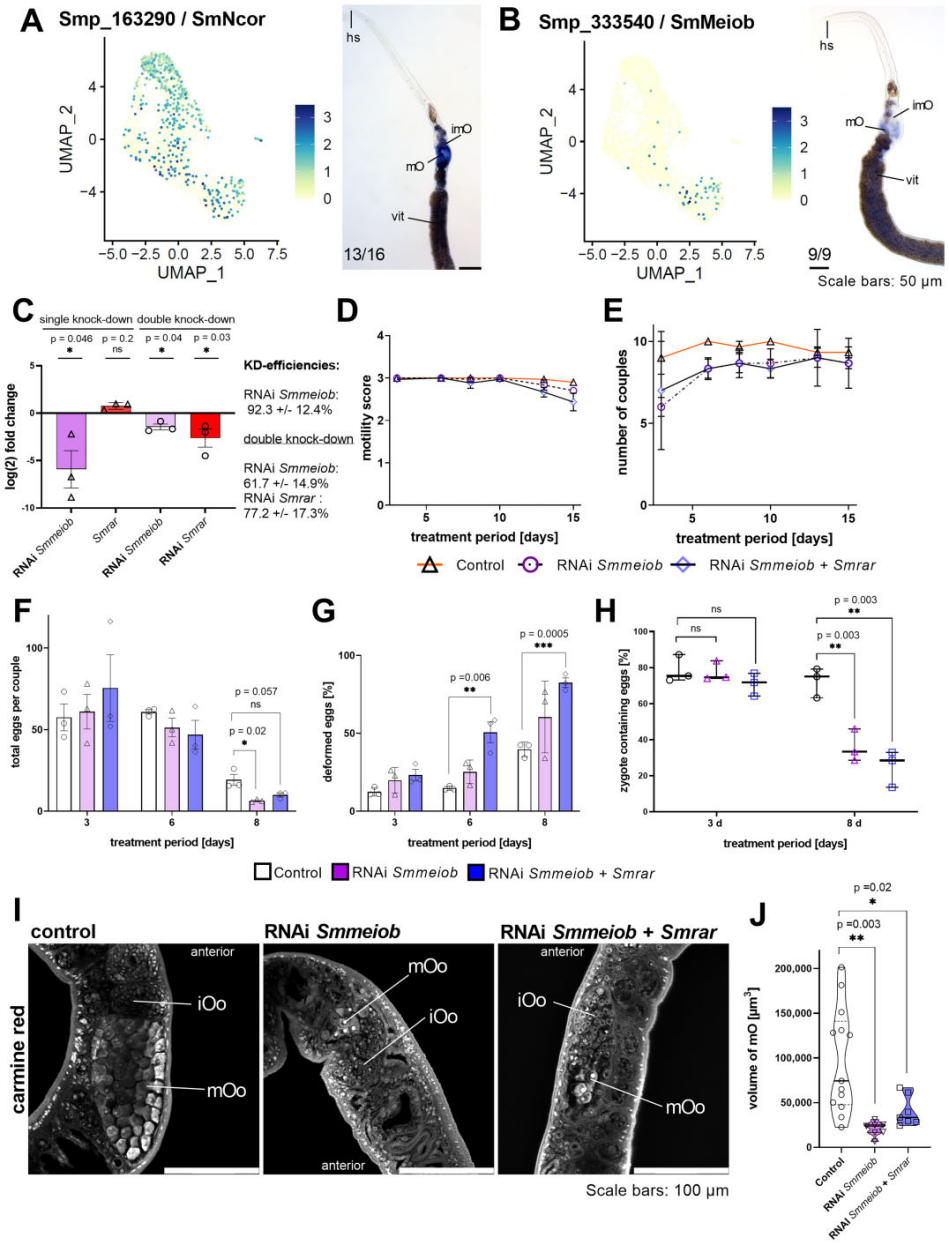
Sm*meiob* RNAi caused a loss of mature oocytes in paired females and showed synergetic effects to Sm*rar* RNAi. (**A, B**), Oocyte scRNA-seq data for Sm*ncor* (**A**) and Sm*meiob* (**B**) demonstrating the occurrence of Sm*ncor* in all clusters, with a preference towards the late germ cells and Sm*meiob* in the GSC/GSC progeny cluster. WISH confirmed the preferential expression of both genes in ovaries. (**C**–**E**) For RNAi, couples were treated with Sm*meiob* (purple, circles) or Sm*meiob*/Sm*rar* (blue, diamonds) dsRNAs at a concentration of 30 μg ml^−1^ each for 15 days (*n* = 3). Control couples (orange, triangles) were treated with DEPC-water. RNAi efficiencies (**C**), motility (**D**), and pairing stability (**E**) were monitored as before. (**C**) Transcript levels of genes following RNAi were determined by RT-qPCR showing significant reduction of Sm*meiob* transcripts (92.3% ± 12.4%). Sm*meiob* single KD caused no significant reduction in Sm*rar* transcript-levels. In the Sm*meiob*/Sm*rar* double KD experiment, transcripts of Sm*meiob* and Sm*rar* were reduced by 61.7% ± 14.9% and 77.2 ± 17.3%, respectively. The graphs show average log_2_fold changes in transcript levels compared to the control and the standard deviation. Data from each biological replicate are indicated. (**F**–**H**) The average number of eggs produced *in vitro*, and the average percentage of deformed eggs were determined during an 8-day RNAi period. We observed a reduction of the number of produced eggs (**F**) and an increase in the number of deformed eggs (**G**). Both RNAi approaches were associated with a significant increase in the number of eggs without zygotes (**H**). The range between maximum and minimum values and the median are represented. (**I**) CLSM analysis of the ovary revealed a reduction in the size of the mature ovary of worms from both RNAi groups and a lower number of mature oocytes. (**J**) Comparative Z-stack analysis of the posterior part of the ovary, containing mature oocytes, revealed a significant reduction in volume induced by Sm*meiob* and Sm*meiob*/Sm*rar* RNAi. The ovaries from females of each biological replicate were examined, whereby each point represents the volume of a single ovary. The violin plot indicates the range between the minimum and maximum values, with the dashed lines representing the quartiles and the solid line representing the median. Abbreviations: hs, head sucker; iOo, oogonia; imO, immature part of the ovary; mOo, mature oocyte; mO, mature part of the ovary; vit, vitellarium. *P < 0.05, **P < 0.01, ***P < 0.001 by *t*-test. Unless otherwise stated, there was no statistically significant difference.

Sm*rar* RNAi resulted in transcriptional downregulation of Sm*meiob*, Sm*ncor*, and Sm*gli1*. To gain insight into the transcriptional patterns of the latter three genes in the ovary, we utilized our scRNA-Seq oocyte data to explore the transcript distribution of these genes within the different oocyte populations represented by the four clusters. We additionally compared these data with the sex- and pairing-dependent transcription profiles of whole worms and their gonads (24,25) and cell-atlas data of adult schistosomes (Supplemental Figure S13 and Supplemental Table S11) (27,32). Furthermore, we used WISH to validate the transcriptional patterns of Sm*ncor*, Sm*meiob* (Figure 9A and B) and Sm*gli1* (Supplemental Figure S13C). For Sm*ncor*, its ovary-preferential transcript pattern predicted by previous whole worm and gonad RNA-seq data (24,25) was confirmed here by WISH; Sm*ncor* transcripts occurred in all ovarian clusters with a preference for intermediate-stage oocytes (Figure 9A and Supplemental Figure S9). According to the scRNA-seq data of bOs obtained in this study, Sm*meiob* transcripts dominated in late oogonia (GSC/GSC progeny), and with low abundance in intermediate-stage oocytes. WISH confirmed the ovary-specific transcript profile of Sm*meiob* (Figure 9B) in paired females, which also agrees with previous bulk RNA-seq analysis (24,25). Moreover, in a previous study (27), cluster validation of the whole worm scRNA-seq atlas by FISH (fluorescence *in-situ* hybridization) confirmed Sm*meiob* transcripts in oocytes of an intermediate (Supplemental Figure S9), and assumed mitotic developmental status. As shown in an independent study, Sm*gli1* transcripts were found in the ventral part of males, independent of pairing, and in females more abundantly in bF than sF (27,28). In females, WISH signals were identified bilaterally along the edges of the male body surrounding the female and its vitellarium (27,28). Our WISH analysis confirmed expression along the vitellarium of bF. In addition, we found weak signals also in the ovary, and here in its posterior part, containing intermediate and mature oocytes (Supplemental Figures S9 and S13). These findings agree with the cell-atlas data of adult schistosomes, in which Sm*gli1* transcripts were found in different tissues including the ovary, although at a low level (27). In our oocyte data set, Sm*gli1* transcripts exclusively occurred in the somatic cluster (Figure 2D and Supplemental Figure 13B), which is characterized by a high abundance of transcripts associated with neuronal, and muscle cells, and the ligament (139,140).

To find supportive evidence for the role of Sm*rar* in oocyte maturation and thus meiosis, we performed further RNAi experiments focusing on Sm*meiob* (Figure 9C–J), Sm*gli1*, and Sm*ncor* (Supplemental Figures S13–S16) as hypothesized partners for functional association. Upon dsRNA treatment, transcript levels of these genes were significantly downregulated in both sexually mature and immature females (re-pairing experiments). Due to partial co-expression in the same oocyte cell cluster and the potential functional overlap between Sm*rar* and Sm*meiob*, double KD experiments were additionally performed to investigate synergetic and enhanced phenotypic effects (Figure 9C–J). To this end, we performed RNAi using 10 couples and 30 μg dsRNA ml^−1^ of each dsRNA per experiment and incubated the worms for 15 days *in vitro*, refreshing medium and dsRNA every 2–3 days. RT-qPCR confirmed significant silencing of both genes in single and double KD approaches (Figure 9C). Sm*rar* transcript levels were not affected by the single KD of Sm*meiob*. This indicates that Sm*meiob* regulation is hierarchically downstream of SmRAR. During the experimental period, we observed a weak influence on motility, and a slightly stronger influence on pairing stability in all treatment groups, but only at the beginning of the experiment (Figure 9D and E). The latter effect normalized over time, and the number of couples was near equal among all groups (including the control) after 15 days.

Egg production and morphology were determined by bright-field microscopy. Egg production was significantly reduced after 8 days in the Sm*meiob* RNAi group (Figure 9F). A similar trend was found for couples of the Sm*meiob*/Sm*rar* double KD group, whereas the number of deformed eggs increased by both RNAi treatments after 6 days, with a significant increase in the number of deformed eggs after double KD (Figure 9G and Supplemental Figure S17). In addition, in both RNAi experiments, we detected a significant reduction in the number of eggs containing a zygote with a significant bias towards the double KD group after 8 days (Figure 9H). CLSM analysis demonstrated an obvious decrease in the volume of the posterior part of the ovary, containing mature oocytes after single and double KD, which was accompanied by a significantly reduced volume of ovaries in both RNAi groups, compared to the control (Figure 9I and J). Since Sm*rar*- and Sm*meiob* are not expressed in the vitellarium, we expected no effect in this organ. Indeed, neither by CLSM (Figure 6C) nor by Oil-Red lipid staining of whole worms (Supplemental Figure S18), we observed any differences to the control. This indirectly supports the proposed ovary-preferential functions of both Sm*meiob* and Sm*rar*.

Since Sm*gli1* was among the transcripts that had been downregulated following Sm*rar* RNAi, we next performed RNAi experiments to find evidence for a potential function of this gene for the ovary. RNAi succeeded, and neither motility nor pairing stability were affected, as expected (Supplemental Figure S13D–F). We detected no effect on ovary structure or volume (Supplemental Figure S13G and H), including general, morphological and sequence-independent dsRNA effects, as recently described for the non-schistosomal *ampR* dsRNA control (65). After 8 days of the RNAi approach, a tendency to reduce egg production was seen *in vitro* but the reduction was not significant. However, a significant increase in deformed eggs by Sm*gli1* RNAi after 8 days was observed, but no difference in the numbers of zygote-containing eggs (Supplemental Figure S14A–D). In addition, lipid staining of Sm*gli1* RNAi-treated schistosomes revealed a comparatively lower staining intensity in the vitellarium compared to females in the control and other RNAi groups (Supplemental Figure S18), as expected (28).

Finally, RNAi against Sm*ncor* showed no RNAi-dependent effects on worm vitality, egg number or morphology (Supplemental Figure S15). However, as shown by KD against Sm*rar*, Sm*nanos1*, and Sm*meiob*, the number of zygote-containing eggs deposited by Sm*ncor* dsRNA-treated worms decreased significantly after 8 days (Supplemental Figure S16A). In addition, the number of proliferating oogonia was significantly reduced, with a trend towards a reduction in the volume of the mature ovary following RNAi at 14 days (Supplemental Figure S16B–F). This is consistent with the scRNA-seq data from mature ovaries and the WISH results showing Sm*ncor* transcription throughout the ovary. In addition, this result supports the importance of NRs and NR-interacting factors in germline progression (21,53,123,141).

## Discussion

With respect to the essential role of female germ cells in embryonic development, understanding the mechanisms controlling their differentiation has enormous significance. Oocytes are of specific interest due to their capacity for totipotency and their irreplaceable role in propagating throughout generations and generating genetic diversity *via* meiotic recombination (142,143). Here, we present the first organ-specific single-cell atlas of ovary cells of a parasitic platyhelminth. This new data set complements previous bulk RNA-seq approaches (18,21) to distinguish gene expression differences in sub-populations of tissues, and it also adds to ovary data of the previous single-cell atlas for adult schistosomes (Supplemental Table S12). Bioinformatics analysis of scRNA-seq data of fully differentiated ovaries from paired *S. mansoni* females resulted in four ovarian cell clusters: somatic cells, germ cells and progeny, intermediate-stage cells and late germ cells. In addition to top marker genes for each cluster, we aimed to identify genes with regulatory function in *S. mansoni* oogenesis. To this end, we focused on the intermediate-stage cluster, assuming its coverage of cells in transition between stem cell-like oogonia and mature primary oocytes, to uncover candidate genes for oocyte differentiation. By combining the previous bulk RNA-seq data on pairing-dependently expressed genes in *S. mansoni* and their gonads (24,25) with the new data set, we discovered genes not identified previously in the single-cell atlas. Furthermore, we assumed critical roles in ovary development and platyhelminth reproduction for these genes, such as Smp_018010, a potential actin-binding ortholog of cytospin-A (CYSTA) (25,41,74). Critical roles for CYSTA in cell division, embryogenesis and interactions with actin and microtubules have been found in human colorectal cancer and embryonic stem cells (144,145). Another example is an uncharacterized ortholog of the Dyp-type peroxidase in schistosomes (Smp_312400) (25,41,74,132). Dyp peroxidases function as cargo proteins, and play a role in iron transport (146). Furthermore, the transcripts of multiple nematode orthologs and known *S. mansoni* key meiosis factors like Sm*vlg2*, Sm*vlg3* (88) and RA signaling factors like Sm*rxr1* (21,130,131) were identified within this cluster. This underpins the overarching convergence of these factors for gonadogenesis, also in trematodes. Next, these examples demonstrate the potential of using scRNA-seq from single organs to gain new insights into the reproductive biology of schistosomes. Moreover, similar approaches could be applied to other platyhelminth species to unravel the function of genes involved in organ differentiation (147).

We predicted a potential role for Sm*rar* in oocyte maturation using KEGG pathway analyses (113,114). This gene exhibited predominant expression in intermediate-stage oocytes and demonstrated low-level transcription in the GSC/GSC progeny cluster. After validating its pairing-dependent and ovary-localized expression in females by RT-qPCR and WISH, respectively, we confirmed its predicted annotation by analyzing its domain structure and phylogeny. NRs share characteristic domain structures with a variable amino-terminal domain comprising several distinct transactivation regions, conserved DBD, a nuclear localization signature and a highly conserved carboxy-terminal LBD (148,149). Because Smp_144170 revealed all typical features of this TF class, which grouped within a subclade of trematode-specific RAR-like receptors, we conclude that it is a member of the family of RAR-like retinoic acid NRs (SmRAR). Our results agree with previous studies of schistosome NRs from the RXR family, where a putatively accelerated evolutionary rate was reported based on the accumulation of more substitutions than their orthologs (4,8).

In vertebrates, retinoid acid NRs, which cover RARs and RXRs, are functionally associated with fertility (150), cell differentiation, embryogenesis, and post-embryonic development (55,151,152). For RARs, critical roles in the initiation of oocyte meiosis, structural gonad organization, and spermatogenesis have been postulated (150,153–156). This may also apply to schistosomes considering the gonad-specific/preferential expression of various retinoid acid NRs (3,24,27), and orthologs of the nematode RA signaling or metabolism pathways (28,85) within the *S. mansoni* ovary. There is growing evidence for RARs in molluscs, hemichordates, sea urchins, and other invertebrates, but little is known about their function (157–159) except in planarians, where a negative effect of RA on the regeneration of the head has been described (160,161). With respect to putative RAR ligands, in vertebrates, RAs play essential roles in the development of the central nervous system (2,55,162,163), the regulation of hormone metabolism (164), tissue homeostasis (165), and cell fate determination (165,166). Among the different RA forms are 9-*cis-*RA (163), 13-*cis-*RA (154,167), and all-*trans*-RA (168,169). In mammals, it was shown that 9-*cis* RA can be the ligand of both RARs and RXRs (84,170). Orthologs of both receptor classes have been found in *S. mansoni* and other schistosome species (35,129–131). For *S. japonicum*, 9-*cis* RA was identified as potential ligand of a SjRXR ortholog (130). Indeed, a positive correlation between 9-*cis* RA and *S. mansoni* reproduction was observed (Supplemental Figure S19A). Using 9-*cis* RA at physiological concentrations, as found in the blood of the portal vein of the final human host (170), led to a significant increase in egg production *in vitro* (Supplemental Figure S19A). In contrast, inhibition of RA signaling led to a significant reduction in egg production without affecting worm vitality (Supplemental Figure S19B). In addition, transcripts of the nematode ortholog of the retinol-binding protein (SmRBP) (85,171) were identified in the intermediate germ cell cluster. Since nematodes such as *C. elegans* and parasitic nematodes are unable to synthesize fatty acids and their derivatives [e.g. retinyl ester (172)] (86,171,173), it has been assumed that fatty acids and retinol are taken up from the diet or the host via RBPs (86), which may also apply to *S. mansoni*. However, the ligands of *S. mansoni* RAR/RXRs remain unknown warranting further studies in the future.

RNAi-based functional analyses of SmRAR with pairing-experienced but also pairing-unexperienced, first-time paired females exhibited reproducible ovary phenotypes that included a smaller organ volume, failure of oocyte differentiation, disturbance of egg formation and a reduced number of zygotes in eggs. These findings pioneer the characterization of SmRAR, extent previous work that addressed *S. mansoni* RA-NRs by characterizing Smp_097700 (SmRXR1) as a constitutively expressed member of the RXRa subfamily (4,21,24,25,27,35,129,131), and finally expanded our current knowledge about the role of trematode NRs in parasite reproduction. Coimmunoprecipitation experiments confirmed heterodimerization of SmRAR with SmRXR1, and this heterodimer was found to bind to the promotor region of the eggshell precursor protein Smp14 *in vitro*, which is tissue-specifically expressed in the vitellarium (21,35). However, the obtained results also emphasized a regulatory role of identified HATs in ovary maturation. In particular, the HATs SmCBP1 (Smp_105910) and SmGCN5 (Smp_070190), for which our oocyte scRNA-seq data indicated co-transcription with SmRAR and SmRXR1 in intermediate oocytes (Supplemental Figure S20), were shown to interact with the SmRXR1/SmRAR heterodimer (21). Subsequently, for RXR/RAR heterodimers, a remodeling of chromatin structure by binding the specific RAR response element (RARE) of a certain promoter was described (174). Chromatin structure is a target of histone modifications, such as HAT-mediated acetylation (175). The SmRXR1/SmRAR-dependent recruitment of specific HATs has been described for *S. mansoni* (21,174). By addressing RAR/RXR-associated HATs in *S. mansoni* with a chemical inhibitor, effects on female reproduction organs were observed (21). Inhibitor treatment caused malformed, smaller eggs, a proliferous vitellarium, and a reduced number of mature oocytes, most of which corresponds to the phenotype observed in our study, except the vitellarium phenotype. This may be explained by the fact that SmRAR is primarily expressed in the ovary of paired females (24), whereas SmRXR1 shows a pairing-independent expression (24) in all tissues (27). Therefore, it seems likely that SmRXR1 may form heterodimers also with other NR partners (176), which could lead to a phenotype mosaic following inhibitor treatment.

In vertebrates, RAR/RXR heterodimers are involved in differentiation processes of gonadal cells regulating mitotic and meiotic processes during spermatogenesis (84,153–156) and oogenesis (177–179). This agrees with our findings, and points to a conserved role of SmRAR in schistosomes with one remarkable exception; SmRAR expression is pairing-dependently regulated with fundamental biological consequences for the female. If essential, pairing-dependently regulated factors, such as SmRAR and SmMEIOB, are missing, the completion of oocyte differentiation fails. In the *S. mansoni* genome, three further retinoic acid receptors are described that belong to the class of all-trans retinoid acid/opsin-NRs (Smp_073470, Smp_097700, and Smp_105090, Figure 4). These paralogs were found to be co-transcribed with Sm*rar* (Supplemental Figure S20) with highest transcript levels in intermediate-stage oocytes, and lowest levels in mature oocytes. This, and the phenotypes on reproduction caused by inhibition of these potential heterodimerization partners (Supplemental Figure S19B), support the hypothesis that SmRAR, and probably also its paralogs, are involved in oocyte differentiation and post oogonia cell division. Furthermore, the existence of RA isoforms opens the possibility of varying dimerization of SmRAR with different RXRs and perhaps other NRs (176,180). In vertebrates, essential roles of RA and RARs in meiosis initiation (150,176,179,181) suggest a similar role of SmRAR and its potential dimerization partner(s) in schistosomes (21).

The hypothesized role of SmRAR in oocyte differentiation and meiosis was reinforced by the analysis of ovarian marker gene expression after SmRAR RNAi. Among these were the tyrosine kinases receptor genes SmFGFR-A and SmFGFR-B, for which previous studies confirmed essential roles in gonad differentiation, stem-cell biology, morphology and embryogenesis (53,182,183). Our former transcriptomics analysis of adult schistosomes and their gonads revealed transcript patterns of both genes in the ovaries with a clear bias towards ovaries of immature females (24). Furthermore, we found no influence of Sm*rar* RNAi on the Sm*nanos1* transcript level (Figure 7A). This was expected since Sm*nanos1*, according to our oocyte scRNA-seq atlas data, is mainly transcribed in GSC/GSC progeny cells and thus may be involved in stem cell-associated processes in oogonia. Our functional analyses supported this conclusion (reduced number of both proliferating oogonia and primary oocytes upon Sm*nanos1* RNAi; Supplemental Figure S8) and thus correspond to previous studies demonstrating a key role in neoblast proliferation (136) and early germline progression (27,87,94,123). Moreover, Sm*nanos1* RNAi caused the depletion of proliferating spermatogonia and azoospermia in testes of treated *S. mansoni* males (123). In the oocyte scRNA-seq dataset, also Sm*fgfr*-*a* / –*b* transcripts occurred mainly in the GSC cluster (Supplemental Figures S6 and S7A). Since Sm*rar* transcripts dominated in intermediate-stage oocytes, we expected no Sm*rar*-induced regulation of Sm*fgfr-a/*-b, which was confirmed by RT-qPCR. This result supports previous conclusion of the roles of Sm*fgfr-a*/ –*b* and Smn*anos1* in controlling stem-cell (oogonia-associated) processes (53,182,184).

Sm*darnt* (Smp_123420) was among the genes predicted by STRING as interaction partner, but according to RT-qPCR analysis it was not regulated following Sm*rar* RNAi. Our previous RNA-seq study revealed a pairing-dependent transcript profile for Sm*darnt* in the ovary (24), and the new scRNA-seq data showed transcript occurrence in intermediate and mature oocytes. ARNTSs and AHRs (aryl hydrocarbon receptors) regulate genes that organize cell differentiation and proliferation (185,186). For instance, in mammalian ovaries, they control fertility and embryogenesis (186,187). Thus, we speculate that although Sm*darnt* may be involved in oocyte maturation in *S. mansoni*, it might be controlled by a Sm*rar*-independent process. However, we cannot rule out a reduction of the Sm*darnt* expression level due to the RNAi-induced decrease of the number of mOo.

In contrast, Sm*gli1* was among the genes significantly downregulated by Sm*rar* RNAi. Sm*gli1* was shown before to control sexual maturation of the female reproductive organs, but not in the maintenance of their differentiation status (28). In vertebrates, Gli orthologs are regulated by the Wnt pathway (188,189), which among others plays an essential role in regulating meiosis-associated genes (179,190). According to BLAST analysis, we obtained evidence that Sm*gli1* may be an ortholog of *C. elegans* SEX-1 (Supplemental Table S6). This gene was found to be a key factor in the RA-regulated organization of the gonads during larval development (85,191). Furthermore, RA isoforms can influence the regulation of Wnt signaling in mammalian (192–194) and nematode (85) cells. Sm*gli1* was found to be expressed in neurons (27,28) but also in and around the ovary of *S. mansoni* females (Supplemental Figure S13B and C) (24,27). We found Sm*gli1* transcripts not in germline oocytes, but in a somatic cluster, characterized by the expression of genes associated with neuronal and muscular functions. These cells could belong to the thin ligamentous, muscular cell layer surrounding the ovary (139,140). Upon Sm*gli1* RNAi in paired females, we observed no morphological changes of the ovary and no significant effects on the number of mOo. However, a key role of SmGli1 in vitellarium differentiation was shown before (28). This might explain the observed changes in egg morphology because vitelline cells provide precursor proteins needed for egg-shell formation (16,195). Since our scRNA-based oocyte clustering showed the occurrence of Sm*gli1* transcripts in a specific subset of (probably) ovary-connected neuronal/muscular cells, we conclude that the observed phenotype is mainly a result of processes in the vitellarium affecting egg-shell synthesis and thus egg formation. However, we cannot exclude additional effects by ovary-‘external processes’ influencing egg formation. These processes are likely independent from zygote formation, which was unaffected by Sm*gli1* RNAi. In vertebrates, RA influences Gli expression in neurons (196–198). Of note, Sm*gli1* and Sm*rar* are co-transcribed in neuronal cells of adults, especially in the neuronal cluster 6 (27). For these and further genes expressed in this specific cluster, pairing-dependent differences of gene regulation were detected (24,27). This paves the way to future studies of neuron 6 cluster in adult worms and their gene repertoires to investigate their contribution to the male-induced maturation of the *S. mansoni* female (199). Since the RT-qPCR approach in our study cannot differentiate between ovary-associated and -intrinsic effects, at this point, it remains an open question whether external or internal role(s) of SmGli1 may influence oogonia division and/or oocyte differentiation in paired females.

Sm*ncor* was also significantly downregulated following Sm*rar* RNAi in paired females. In vertebrates, NCoR, also known as TRAC, is part of chromatin remodeling complexes and regulates different processes (25,200). Its most prominent function is the repression of RAR heterodimers (201). This way, NCoR influences cell-fate determination, cell differentiation, linage progression, and the regulation of epigenetic modifications (201–205). GEI-8, a NCoR/SMRT ortholog in *C. elegans*, is involved in muscle physiology (205) and arrests gonadogenesis upon site-specific mutation (141). Sm*ncor* is expressed in a pairing-independent manner in both male and female worms, with highest levels in oocytes (24), and it localized in all four oocyte clusters with a preference for intermediate and mature oocytes. This correlates with our WISH results and the RNA-seq data for adult worms previously published (27). Sm*ncor* RNAi showed a reduction in the overall size of the ovary, as well as a significant reduction of zygote-containing eggs. However, in contrast to paired females Sm*ncor* was not significantly downregulated in first time-paired females after Sm*rar* RNAi – an observation that we cannot yet explain.

Sm*bmpg*, encoding a putative bone marrow proteoglycan homolog, was also downregulated following Sm*rar* RNAi. Sm*bmpg* and its planarian ortholog (SMED30019646) were previously described as marker genes for mature oocytes during the data analysis of the adult *S. mansoni* cell atlas (27) and the analysis of female germ cells of *S. mediterranea* (135), respectively. These findings are consistent with our scRNA-seq data, as we found the highest levels of Sm*bmpg* transcripts in mature oocytes. Additionally, it fits to the Sm*rar* RNAi data, as a reduction of the number of mature oocytes would lead to an overall reduction of Sm*bmpg* transcripts.

Finally, the Sm*meiob* transcript level was significantly reduced by Sm*rar* RNAi. In contrast to Sm*ncor* and Sm*bmpg*, Sm*meiob* was found to be exclusively transcribed in the male testis and female ovary (25,199), and it is pairing-dependently regulated with a bias to immature oocytes (24,27). From its predicted structure, SmMEIOB showed highest similarity to the human OB-containing, meiosis-specific homolog RPA1 (MEIOB; Supplemental Figure S21) (206). Key roles of RARs in the regulation of meiosis-associated genes such as *meiosin, stra8*, and *meiob* were reported for mammalian germ-cell differentiation (207–209). Our clustering analysis indicated Sm*meiob* expression in cells representing the GSC progeny cluster and differentiating oocytes (intermediate-stage oocytes) (27,210). Vertebrate orthologs of SmMEIOB play essential roles in meiotic recombination during oogenesis and spermatogenesis in human and other organisms (38,39,211–213). As ssDNA-binding protein, MEIOB is an essential regulator of the recombination between paired chromosomes during meiosis I (38,39,206). In this context, a correlation between MEIOB loss-of-function and premature ovarian insufficiency has been demonstrated (214). Our RNAi experiments revealed a comparable role of Sm*meiob* in oocyte maturation for *S. mansoni*, which represents the first functional characterization of a helminth ortholog of MEIOB. The similarities of the RNAi phenotypes of Sm*rar* and Sm*meiob* and the additive effects of the double KD, which affected the number of mature oocytes and zygote-containing eggs (Figure 9), support the conclusion that both genes contribute to meiosis regulation in *S. mansoni* oocytes.

A weakness of our study is that due to the absence of validated antibodies and established protocols for studying meiotic processes at the cell and chromosomal levels in adult schistosomes, we could not determine the exact stage of the meiotic arrest following Sm*rar* and Sm*meiob* RNAi.

In summary, we created the first organ-specific scRNA-seq atlas of a platyhelminth using the model parasite *S. mansoni*. Our findings point out the value of subtranscriptomics of organs and tissues complementing existing scRNA-seq data sets for *S. mansoni* and other platyhelminths (135,147,215). Our analysis revealed four distinct cell clusters within the ovary of mature, paired females: GSC/GSC progeny, intermediate-stage oocytes, mature oocytes (representing primary oocytes), and somatic cells that exhibited features of both muscle cells and neurons. It is tempting to speculate that these somatic cells may fulfil comparable roles as somatic ovarian cells in planarians that form a functionally distinct compartment in the ovary of *S. mediterranea* providing factors important for oocyte differentiation (135). This will be subject of future studies. The function of SmRAR as NR of the retinoic acid receptor family and its role in regulating Sm*meiob* and Sm*ncor* imply an additive influence of RA, and thus, the host environment, on one of the most crucial steps of schistosome female sexual reproduction, oocyte differentiation. Pilot experiments with 9-*cis* RA led to increased egg production of *S. mansoni* couples *in vitro*, and RA signaling inhibition caused the opposite (Supplemental Figure S19), which reinforces this assumption. Therefore, our study further increases our knowledge about the fascinating sexual biology of schistosomes, and we hope that our contribution will be stimulating for researchers to explore the largely unexplored field of TF/NR studies in platyhelminth development.

## Supplementary Material

gkae1228_Supplemental_Files

## Data Availability

Raw sequence data were deposited in the European Nucleotide Archive (ENA) under the project accession ERP137193, ERS11891010 (bisex ovaries; mO). The clustering data can be interactively explored at http://gonadsc.schisto.xyz/.
