# Supplementary material for: The retinoic acid family-like nuclear receptor SmRAR identified by single-cell transcriptomics of ovarian cells controls oocyte differentiation in Schistosoma mansoni
Source: Nucleic Acids Res. 2024 Dec 16;53(4):gkae1228. doi: 10.1093/nar/gkae1228 (PMC11879061; doi:10.1093/nar/gkae1228)
Supplement: gkae1228_Supplemental_Files [file gkae1228_supplemental_files.zip › Supplemental data.pdf]

Supplemental data

Supplemental Figures (S1-S21; pages 1-26) and Supplemental Tables (S1-S12; pages 27-37)

Supplemental Figure S1. Structure of *S. mansoni* composite eggs and egg deformity variants

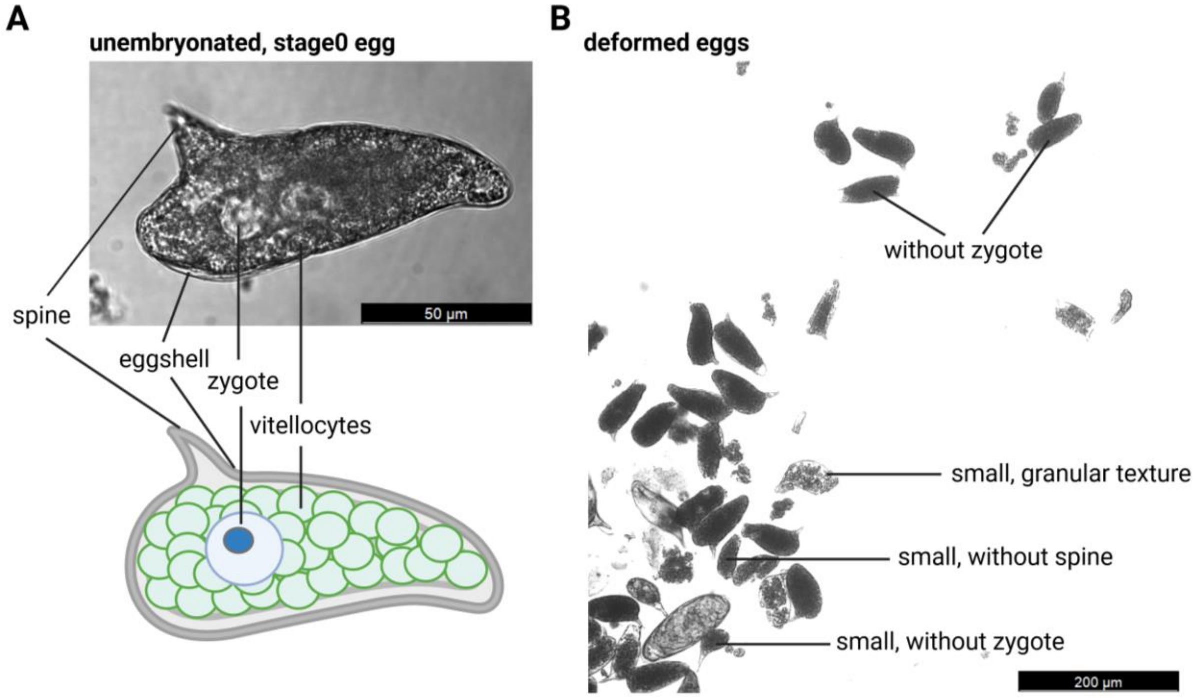

Suppl. Fig. S1: A, Representative images of a normal shaped, zygote containing stage0 egg according to Jurberg´s classification (17, 216, 217). *S. mansoni* eggs show all the typical characteristics of composite trematode eggs, i.e. a single zygote surrounded by 30-40 vitellocytes (yolk cells), protected by a resistant eggshell (17). **B**, Examples of different types of deformed eggs.

216. Prata,A. (1957) Biópsia retal na esquistossomose mansoni: bases e aplicações no diagnóstico e tratamento. In *Biópsia retal na Esquistossomose mansoni: bases e aplicações no diagnóstico e tratamento*. p. 198-198.

217. Vogel,H. and Vogel,H. (1942) Über Entwicklung, Lebensdauer und Tod der Eier von *Bilharzia japonica* im Wirtsgewebe. *Dtsch. Tropenmedizinische Z.*, 46, 57–91.

Supplemental Figure S2. Gene expression of novel germ cell marker genes

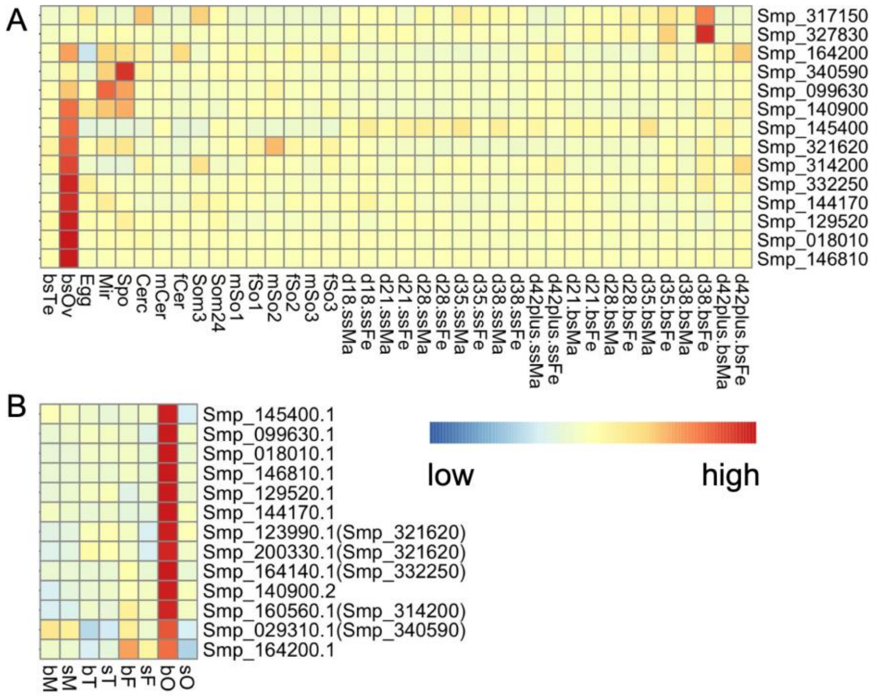

Suppl. Fig. S2: A, RNA-seq gene expression in v7 (data obtained from https://v7test.schisto.xyz)(25). **B**, RNA-seq expression data in v5 (71). Expression values were scaled by row to highlight preferential expression in certain life stages/samples.

Supplemental Figure S3. Protein structure of SmRAR and its orthologs

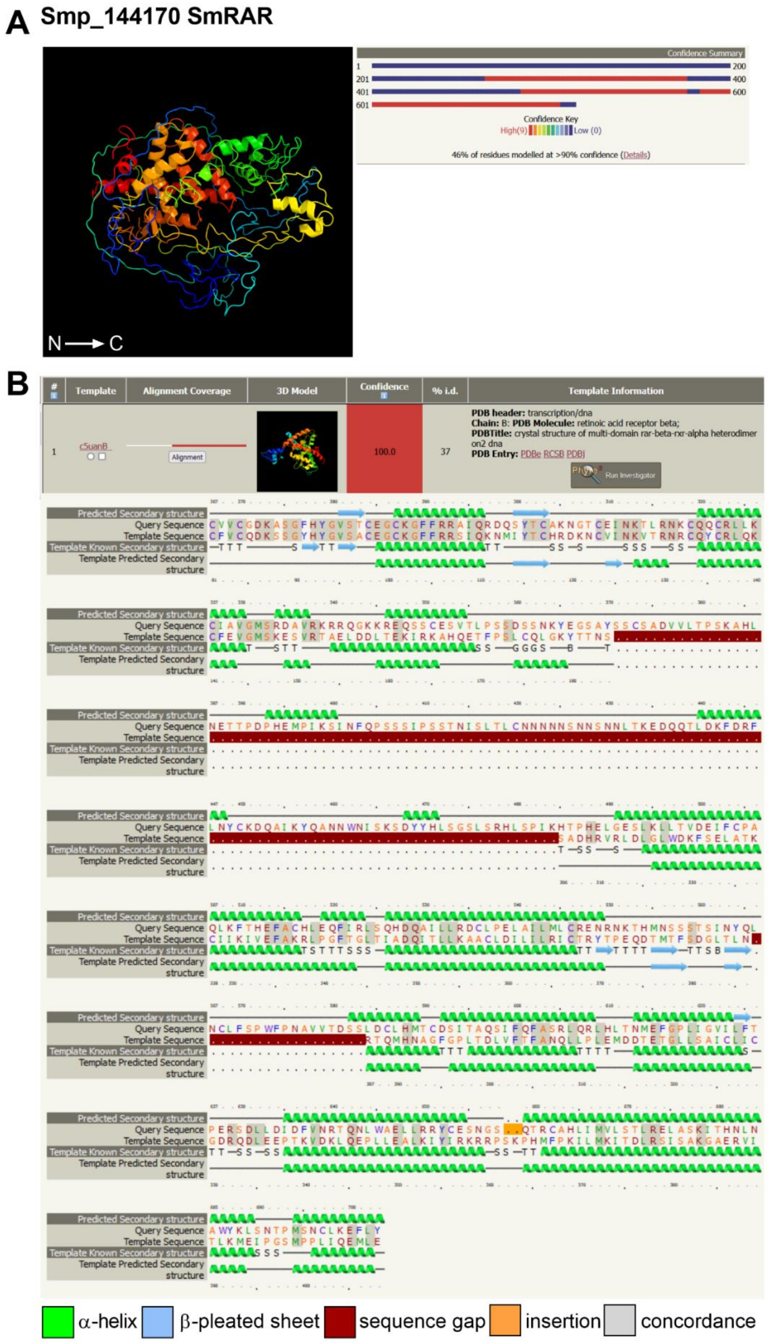

Suppl. Fig. S3: **A,** The protein structure of SmRAR was modelled by using Phyre2 (75) based on known structures of orthologous nuclear receptors of the protein data bank (PDBe). **B**, The amino acid sequence and protein structure of SmRAR were compared to characterized nuclear receptors from model organisms using Phyre2. Using alignment coverage, SmRAR was verified as nuclear receptor and was assigned with highest similarity to the retinoic acid receptor beta subfamily.

Supplemental Figure S4. Treatment of schistosome couples with non-schistosomal control dsRNA showed no alteration of the ovary structure

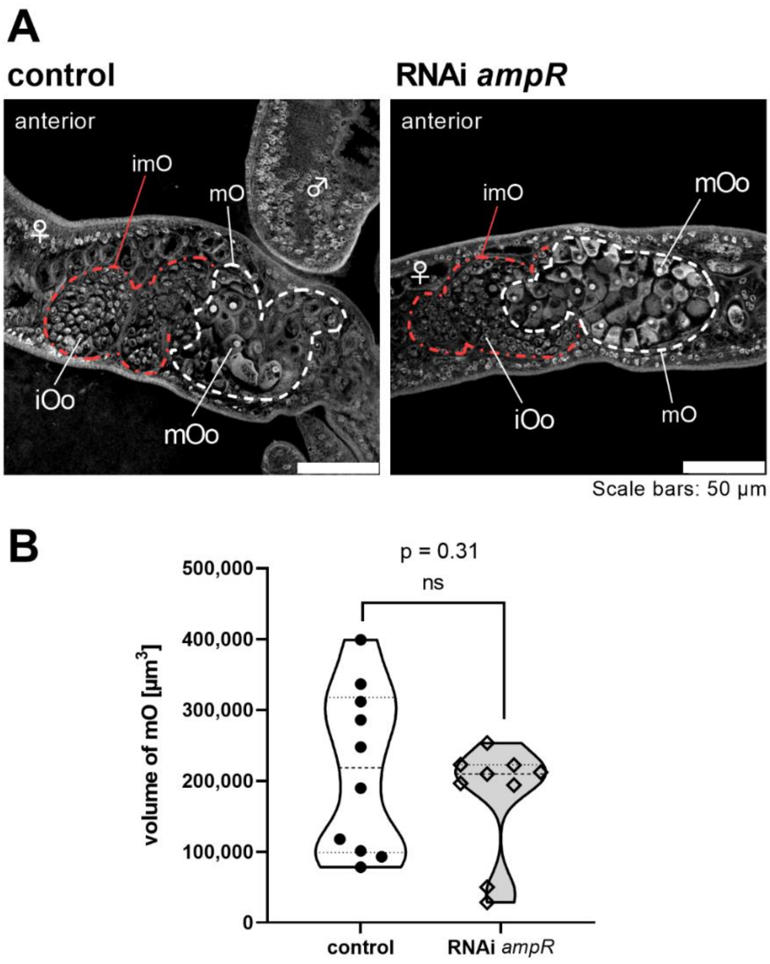

Suppl. Fig. S4. Results of RNAi experiments using dsRNAs against Sm*rar* and *ampR* (ampicillin resistance gene of *E. coli*) as irrelevant dsRNA control (65), respectively. In each case, worms were treated with 30 µg/mL dsRNA for 22 d. **A**, CLSM showed no obvious differences in ovary morphology between DEPC-treated control worms and *ampR* dsRNA-treated worms. **B**, The volume of the ovary was determined by comparative Z-stack analyses post RNAi at d 22. No effect on the volume of the posterior, mature part of the ovary was observed in the *ampR* RNAi group. The violin plot indicates the range between the minimum and maximum values, with the dashed lines representing the quartiles and the solid line representing the median. The ovaries of worms from three biological replicates were analyzed. The graph shows the pooled results of all analyzed ovaries. Each individual point represents the volume of a single ovary. Abbreviations; iOo, oogonia; imO, immature part of the ovary; mOo, mature oocyte; mO, mature part of the ovary. *P<0.05, **P<0.01, ***P<0.001 by t-test. *n* = 3

Supplemental Figure S5. Sm*rar* dsRNA caused no morphological changes in the testes of paired male schistosomes

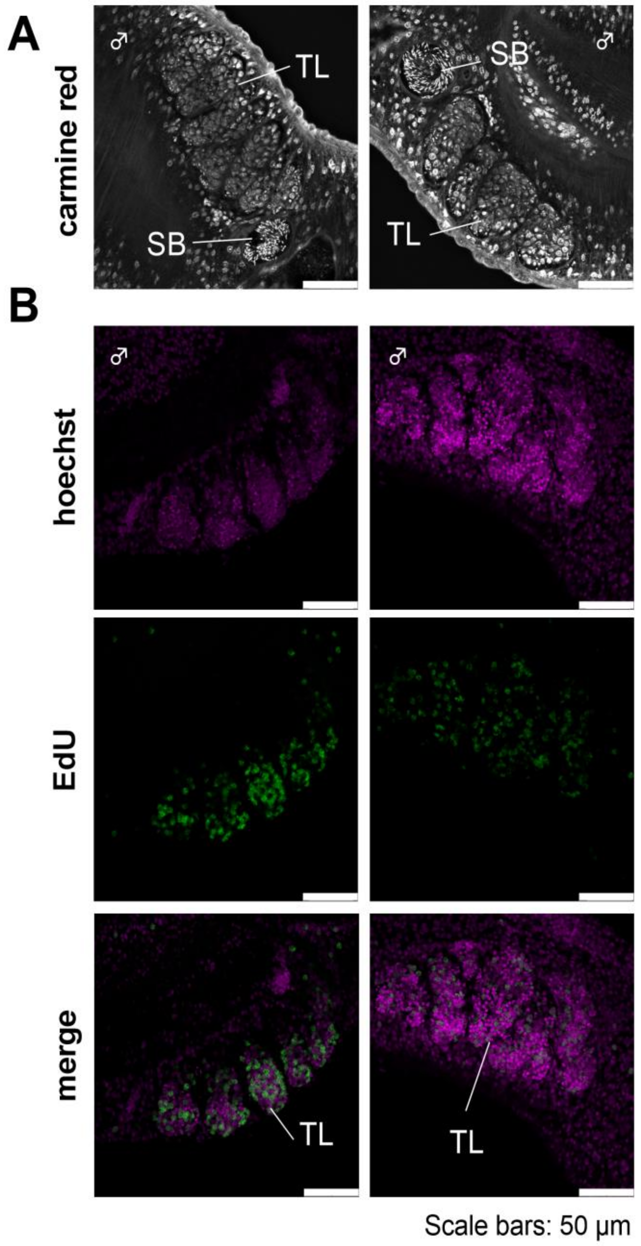

Suppl. Fig. S5: Couples were treated with 30 µg ml^{-1} Sm*rar* dsRNA every 2 to 3 days over a period of 15 days. **A**, After treatment, couples were examined by CLSM. Representative pictures of the male reproduction organs are shown for control and dsRNA-treated worms, respectively. We observed no morphological differences between the different groups. **B**, EdU was added 24 h, before the treatment was stopped. In both experimental groups, no differences in the number of EdU-positive cells occurred in male reproductive organs. Abbreviations: SB, Seminal bladder; TL, testicular lobes.

Supplemental Figure S6. Developmental trajectory of immature oocytes within the GSC/GSC progeny cluster

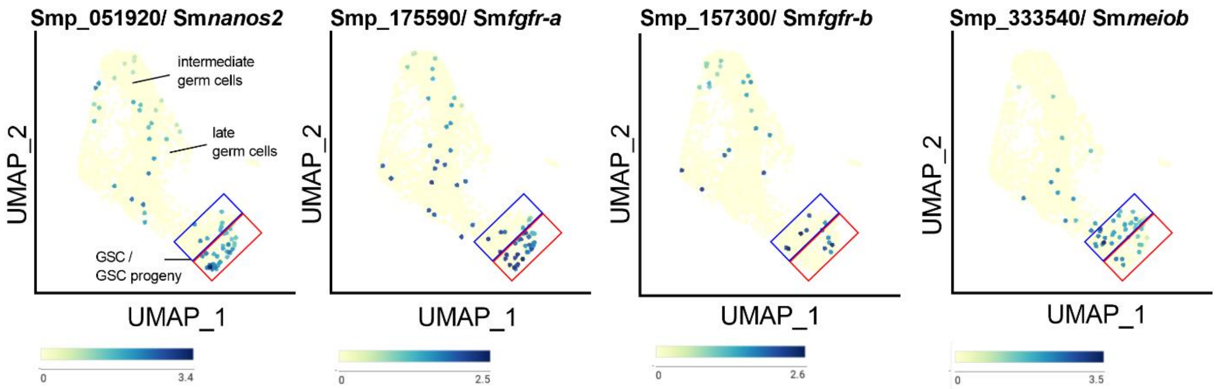

Suppl. Fig. S6: Analysis of the GSC/GSC progeny cluster showed differences in the expression pattern visualized by UMAP projection. These patterns were assigned to specific stages of development, as indicated by colored boxes. The red box indicates genes with transcriptional peaks, such as Sm*nanos2*, whereas the blue box indicates genes with transcriptional peaks, such as Sm*meiob*. Sm*nanos2* was found to correlate with S-phase EdU-positive cells, while Sm*meiob* correlates with EdU-negative cells (27). The analysis revealed that Sm*fgfr-a* shares a greater similarity to the transcription pattern of Sm*nanos2* and is therefore transcribed in early oocyte development. Sm*fgfr-b* showed a transcription pattern intermediate between that of Sm*nanos2* and Sm*meiob*.

Supplemental Figure S7. Localization and functional analysis of Sm*nanos1* by RNAi showed oogonia expression and a trend of morphological alteration of *in vitro* laid eggs

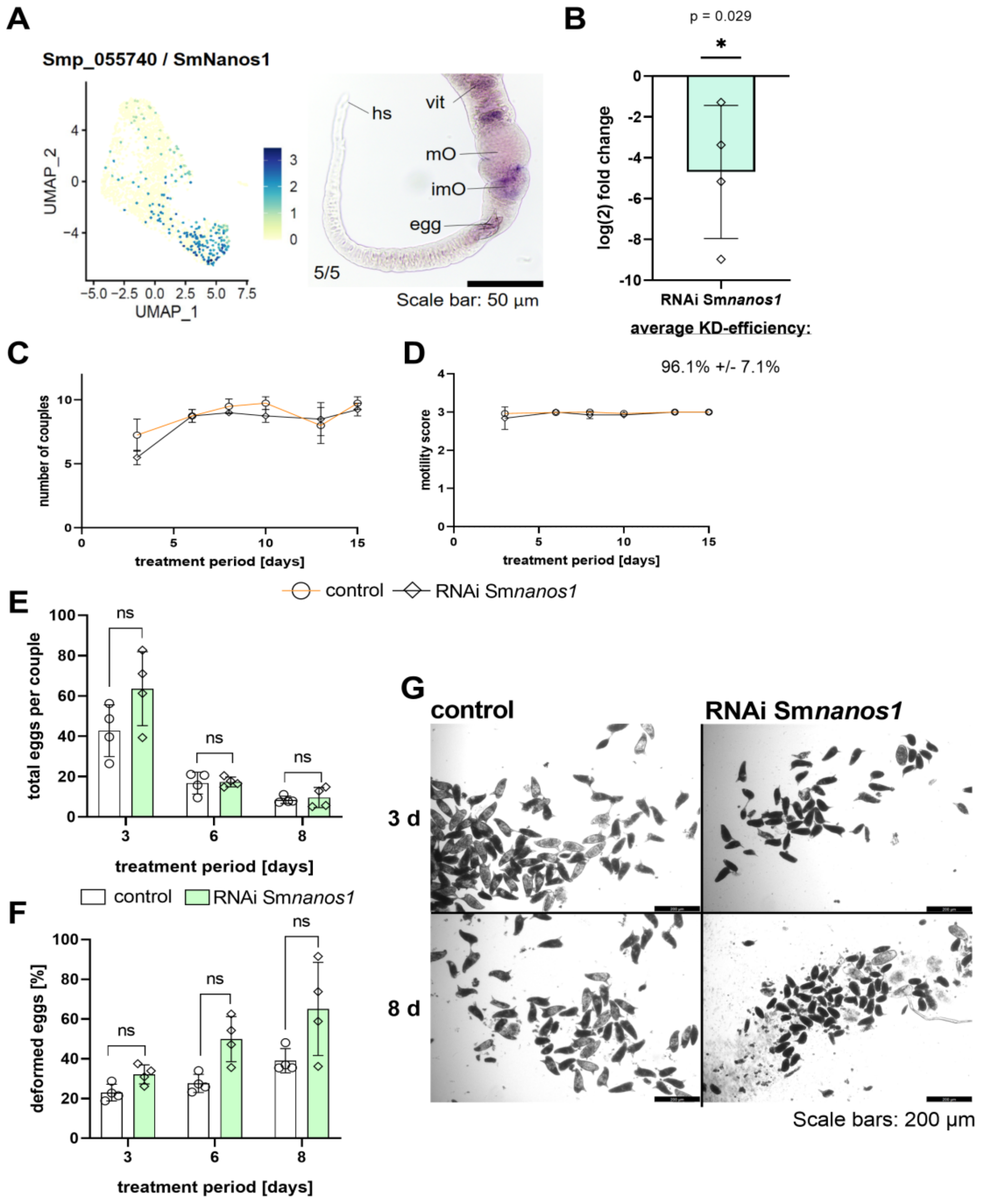

Suppl. Fig. S7: A, Oocyte scRNA-seq data demonstrating the dominant occurrence of Sm*nanos1* transcripts in the GSC/ GSC progeny cluster. WISH confirmed the preferential expression of Sm*nanos1* in the anterior part of the ovary. **B-G**, For RNAi, couples were treated with Sm*nanos1* (green, circles) dsRNA at a concentration of 30 µg ml^{-1} each for 15 days (n = 4). Control couples (orange, circles) were treated with DEPC-water. RNAi efficiencies (**B**), motility (**C**), and pairing stability (**D**) were monitored as before. **C**, Transcript levels of genes following RNAi were determined by RT-qPCR showing significant

reduction of Sm*nanos1* transcripts (98.1% ± 7.1%). The graphs show average log(2) fold changes in transcript levels compared to the control and the standard deviation. Data from each biological replicate are indicated. **E-G**, The average number of eggs produced *in vitro*, and the average percentage of deformed eggs were determined during an 8-day RNAi period. We observed no reduction of the number of produced eggs (**E**), but a tendency increase in the number of deformed eggs (**F**, **G**). $^{*}P < 0.05$, $^{**}P < 0.01$, $^{***}P < 0.001$ by t-test. $n = 4$.

Supplemental Figure S8. Sm*nanos1* RNAi resulted in a significant reduction of the number of zygote-containing eggs, the number of proliferating oogonia, and oocyte deficiency

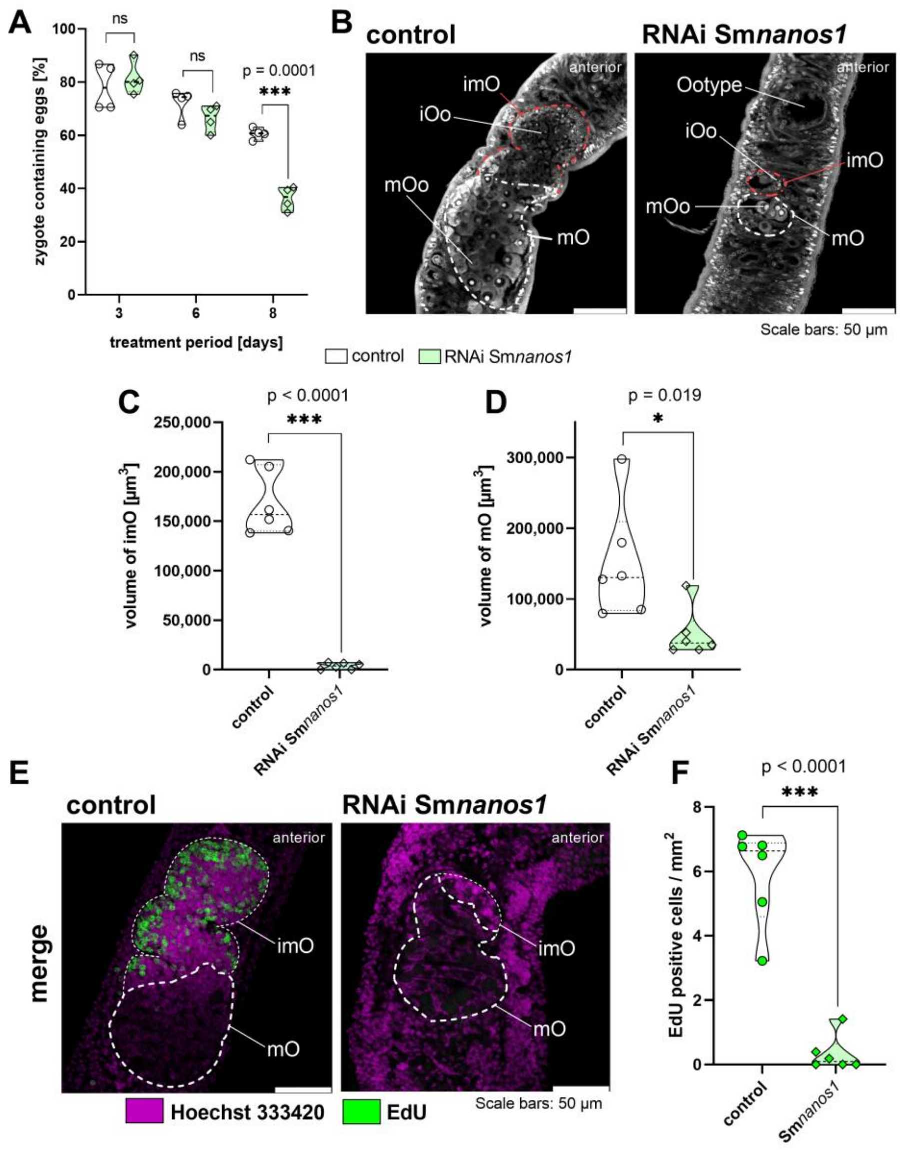

Suppl. Fig. S8: Schistosome couples were treated with 30 µg ml^{-1} Sm*nanos1* dsRNA for 15 days. Subsequently, the number of zygote-containing eggs were estimated (**A**), and the ovarian structure analyzed (**B-E**). **A**, Weighted distribution (violin plot) of zygote-containing eggs upon dsRNA treatment. After 8 days, a significant reduction in zygote-containing eggs was observed for dsRNA-treated couples (green) compared to controls (white). **B**, CLSM analysis of paired worms showed a reduction of the overall size of the ovary of dsRNA-treated couples. Violin plot of the distribution in volumes of the anterior (**C**) and posterior (**D**) part of the ovaries based on comparative Z-stack analysis of carmine-red stained females. The ovaries from females of four biological replicates were examined; each point represents the volume of a single ovary. A significant reduction in the volume of both, the anterior and posterior part of the ovary was observed in the RNAi group. **E**, CLSM analysis of EdU-treated female

schistosomes showed a strong reduction in the abundance of stained cells in the RNAi groups. In the control group, signals (green) occurred in the anterior part of the ovary, which contains immature oocytes (oogonia). The dimensions of these ovary sections were clearly reduced in females of the RNAi group. Cell nuclei were counter-stained with Hoechst 33342 (purple). **F**, Violin plot of the distribution of proliferating cells in the ovary in the plane of maximum extension. A significant RNAi-dependent reduction in the number of proliferating cells was observed. In the violin plots, individual values of biological replicates are shown as well as their median values (solid lines). Abbreviations: iOo, oogonia; imO, immature part of the ovary; mOo, mature oocyte; mO, mature part of the ovary; P, parenchyma; T, tegument; Vit, vitelline lobe; z, zygote. *$P < 0.05$, **$P < 0.01$, ***$P < 0.001$ by t-test. $n = 4$.

Supplemental Figure S9. Germline linage in the *S. mansoni* ovary

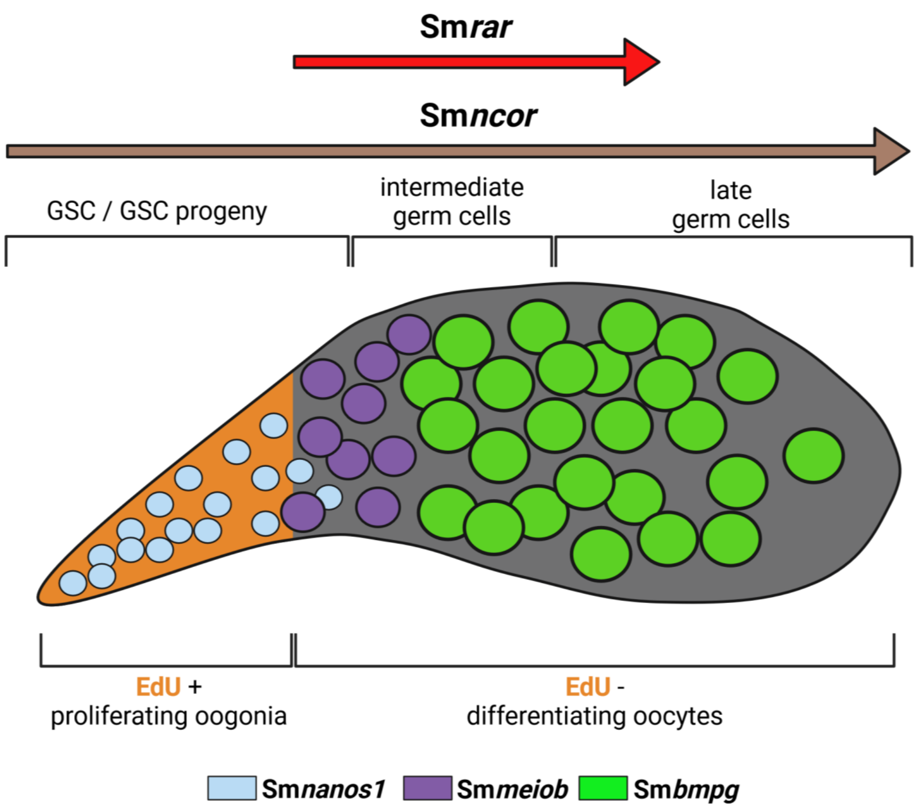

Suppl. Fig. S9: Illustration of the germline linage of the mature schistosome ovary according to Wendt *et al.*(27). Additionally indicated is the differential transcription of Sm*rar* (red arrow) and Sm*ncor* (brown arrow) among the ovarian clusters according to the oocyte scRNA-seq atlas of isolated mature ovaries. By applying FISH and WISH, Sm*nanos1* (cyan) was found to be transcribed mainly in cells of the proliferating, EdU-positive GSC/GSC progeny (oogonia, orange) (27). Sm*meiob* was detected to be transcribed in EdU-negative (grey), differentiating cells belonging to the GSC progeny and oocytes of intermediate development, whereas Sm*bmpg* (green) transcripts were enriched in late female germ cells, which represent mature oocytes (primary oocytes) (27).

Supplemental Figure S10. The meiosis-associated genes Sm*meiob* and Sm*bmpg* were significantly down-regulated following Sm*nanos1* RNAi

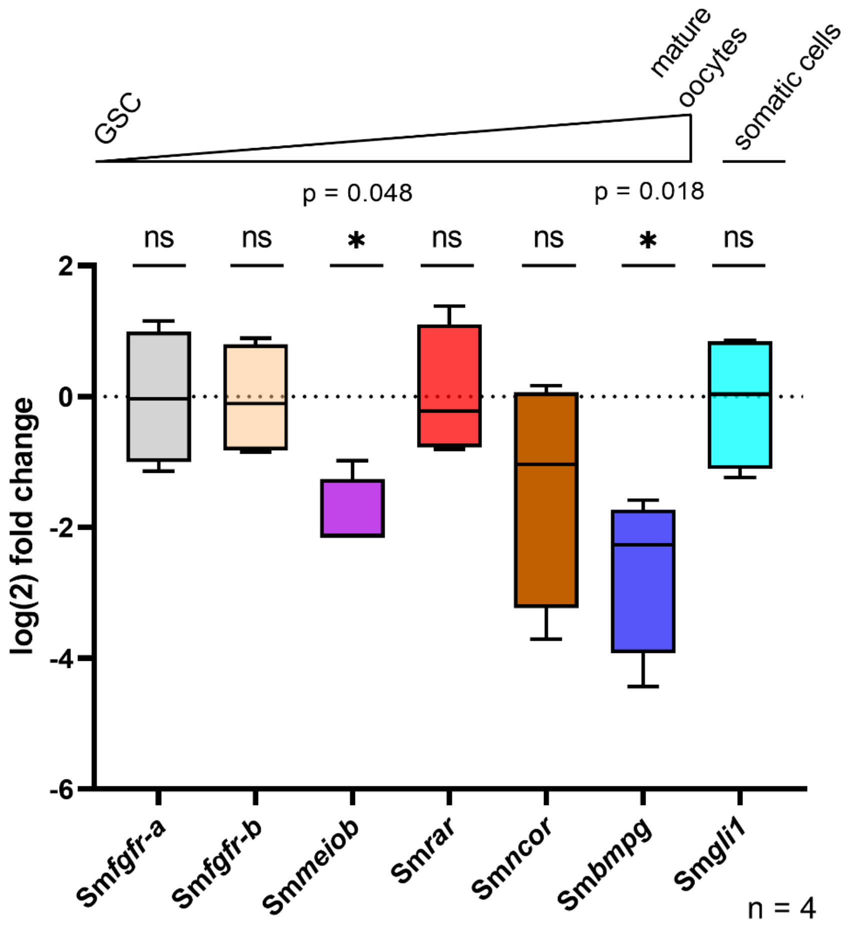

Suppl. Fig. S10: Schistosome couples were treated with 30 µg ml^{-1} Sm*nanos1* dsRNA for 15 days, before females were separated from males for RNA isolation. Subsequently, transcript levels of candidate genes were quantified by RT-qPCR (*n* = 4). Candidate genes, also used for the analysis of the Sm*rar* RNAi, were examined. Initially, these genes were identified as interacting partners of Sm*rar* predicted by STRING network analysis (82, 83), or they had been shown previously to be associated with different stages of ovary development (27, 53, 135). The results showed significant reductions of the transcript levels of Sm*meiob* (Smp_333540*,* purple) and Sm*bmpg* (Smp_ 078720, blue), and a tendency in the downregulation of Smp_163290 (Sm*ncor,* brown). The transcriptional changes of the analyzed genes were plotted following the oocyte developmental trajectory; from immature, undifferentiated GSCs to mature oocytes. They were arranged according to their transcription peaks of the respective cluster of the scRNA-seq atlas of mature ovaries. The boxplot indicates the range between minimum and maximum values, with the 10th and 0th percentiles and the median shown in the box. *P < 0.05, **P < 0.01, ***P < 0.001 by t-test.

Supplemental Figure S11. RNAi with a non-schistosomal dsRNA showed no effects on ovary structure or oocyte differentiation

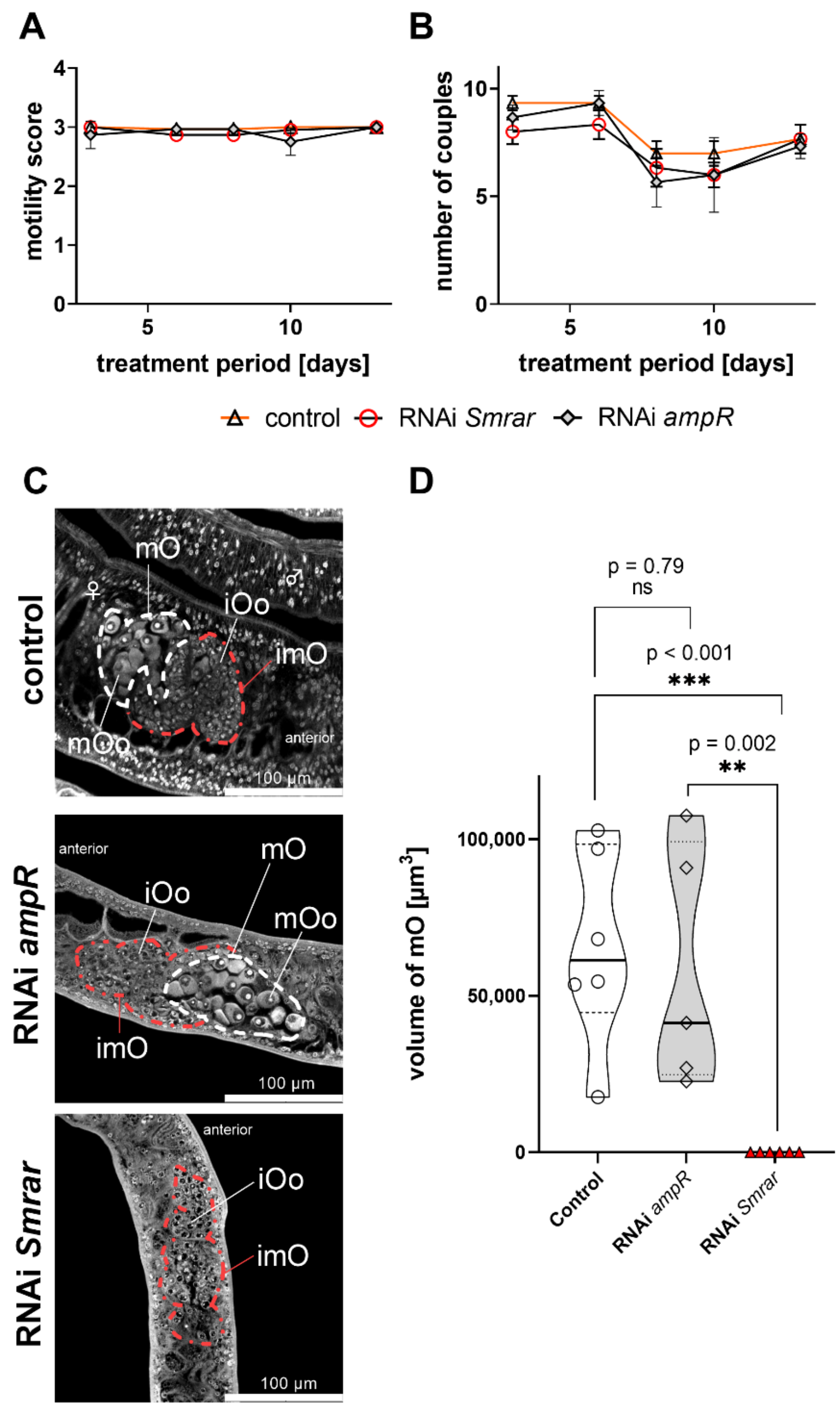

Suppl. Fig. S11: Results of RNAi studies using dsRNAs against Sm*rar* or *ampR* (ampicillin resistance gene of *E. coli*) as irrelevant dsRNA control (65), respectively. Physiological parameters such as motility and pairing stability were monitored (Sm*rar*-specific dsRNA, red circles; *ampR* control dsRNA, grey diamonds). Worms treated with DEPC water, but no dsRNA, served as untreated control (orange triangles). **A-B**, RNAi treatment showed no effect on motility (**A**, motility scores: 0 (no motility) to 4

(hyperactive motility)). Couples remained stable during the experimental period (**B**). **C**, CLSM showed no obvious differences in ovary morphology between DEPC-treated control worms and *ampR* dsRNA-treated worms, whereas Sm*rar* RNAi showed strong effects on the ovary structure and oocyte differentiation, as described in the main text. **D**, The volume of the mature ovary was determined by comparative Z-stack analyses. No effect on the volume of the posterior, mature part of the ovary was observed in the *ampR* dsRNA-treated control group. The violin plot indicates the range between the minimum and maximum values, with the dashed lines representing the quartiles and the solid line representing the median. The ovaries of worms from three biological replicates were analyzed. Each individual point represents the volume of a single ovary. Abbreviations; iOo, oogonia; imO, immature part of the ovary; mOo, mature oocyte; mO, mature part of the ovary. $*P < 0.05$, $**P < 0.01$, $***P < 0.001$ by t-test. $n = 3$.

Supplemental Figure S12. Sm*rar* RNAi in first-time paired females caused decreased egg production, deformed eggs, and eggs without zygotes

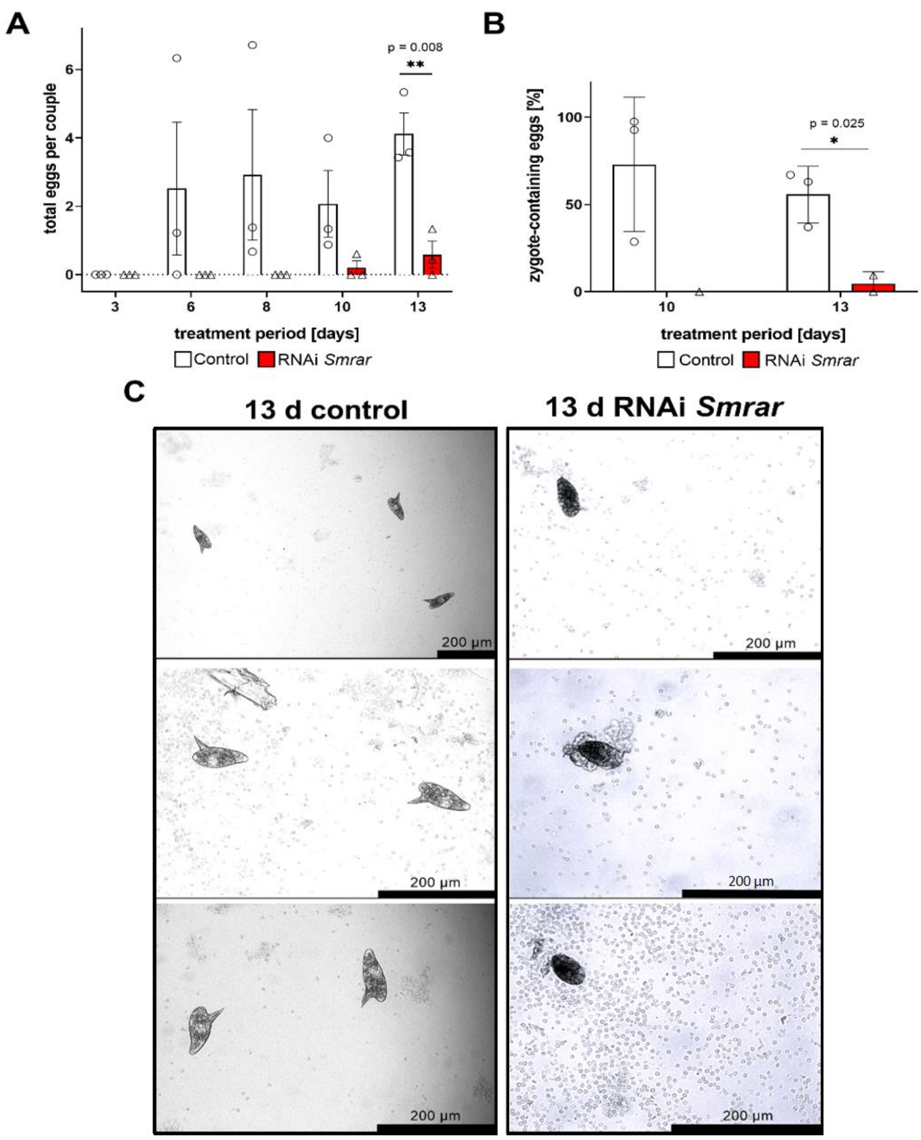

Suppl. Fig. 12: A-C, Summary of experiments with first-time paired females following Sm*rar* RNAi. **A**, After pairing of dsRNA-treated females without previous pairing-experience (sF) with pairing-experienced males (bM), the egg production was monitored. Control couples (without dsRNA; white columns) showed egg production 5 days earlier and produced significantly more eggs compared to Sm*rar* dsRNA-treated couples (red columns). **B**, Eggs produced by the Sm*rar* RNAi groups showed a significantly reduced zygote content. **C**, Bright-field microscopy showed changes in egg morphology in the Sm*rar* RNAi group, characterized by a reduction in size and altered eggshell morphology such as reduced or missing spines. The circular structures in the background are blood cells that are part of the used Basch ABC/LDL medium. *$P < 0.05$, **$P < 0.01$, ***$P < 0.001$ by Mann-Whitney test. $n = 3$.

Supplemental Figure S13. Sm*gli1* RNAi caused no alterations of the ovary

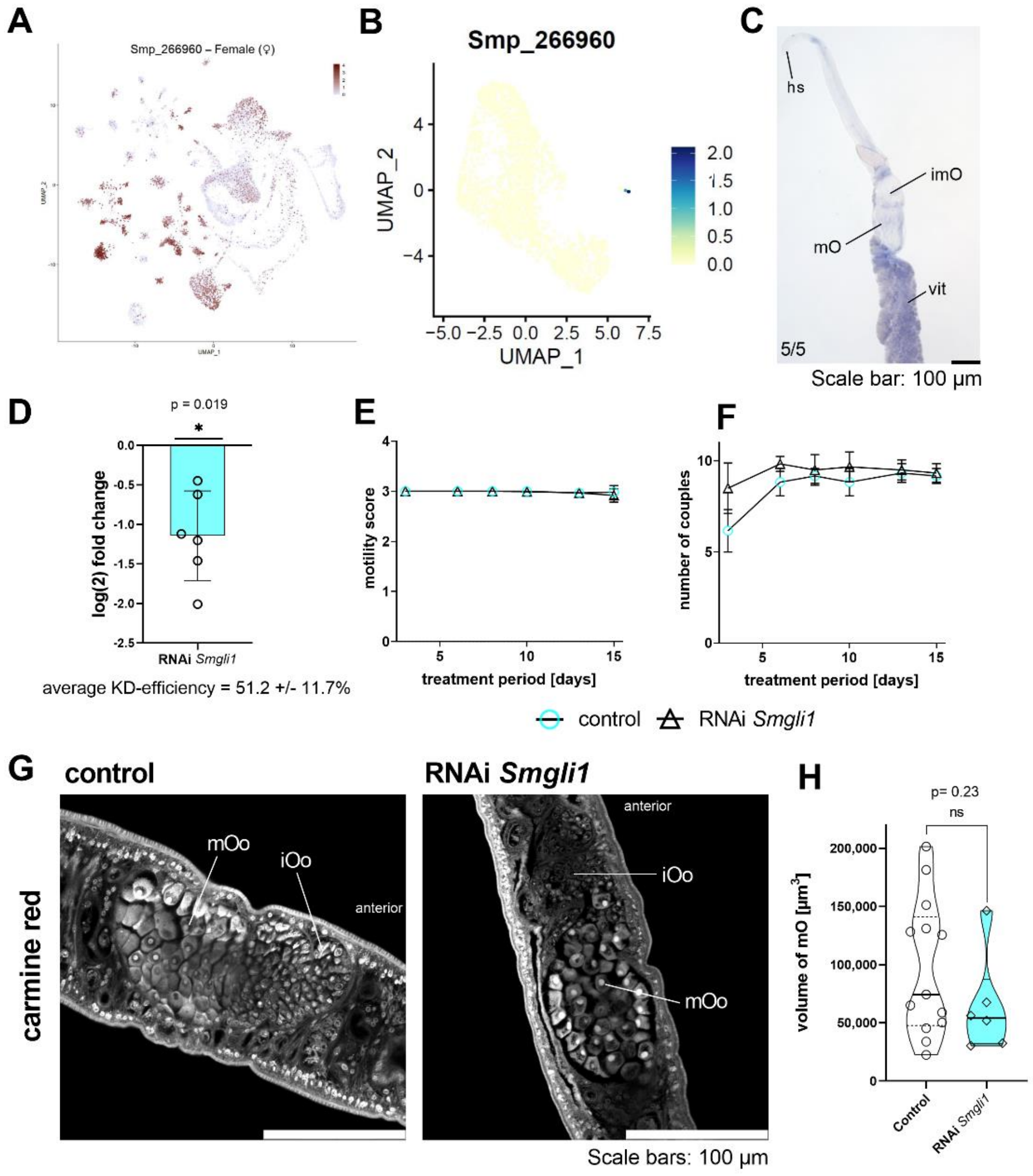

Suppl. Fig. S13: A, The cell atlas of whole *S. mansoni* worms from Wendt *et al.* (27, 32) showed Sm*gli1* transcripts in neuronal and parenchymal cells, muscles, neoblasts, and gonadal cells. **B**, The oocyte scRNA-seq atlas of our study showed enriched Sm*gli1g* transcripts in a somatic cluster characterized by transcripts of genes associated with neuronal and muscle cells. **C**, As expected, WISH of paired female schistosomes localized Sm*gli* transcripts in different tissues, consistent with previous findings (28). In the ovary, weak Sm*gli1* signals were found in the larger part of the ovary containing intermediate stage and mature oocytes. **D**, RT-qPCR determined a significant reduction of Sm*gli1* transcripts following RNAi. The average log(2) fold change in transcript levels compared to the control and the standard deviation are illustrated. Each biological replicate is represented by a single point. **E-**

F, Motility (**E;** motility scores: 0 (no motility) to 4 (hyperactive motility)) and pairing stability (**F**) were monitored during the RNAi experiment showing no significant differences between the experimental groups. **G**, CLSM analysis showed no RNAi-dependent changes in ovary morphology and/or the presence of immature and mature oocytes. **H**, Determination of the volume of the posterior part of the ovary by comparative Z-stack analyses showed no Sm*gli1* RNAi-associated volume reduction. The violin plot shows the range between the minimum and maximum values, with the dashed lines representing the interquartile range and the solid line representing the median. The ovaries of worms from six biological replicates were analyzed. Each point represents the volume of a single ovary. Abbreviations: hs, head sucker; iOo, oogonia; imO, immature part of the ovary; mOo, mature oocyte; mO, mature part of the ovary; vit, vitellarium. *$P < 0.05$, **$P < 0.01$, ***$P < 0.001$ by t-test. $n = 6$.

Supplemental Figure S14. Sm*gli1* RNAi caused changes in egg morphology

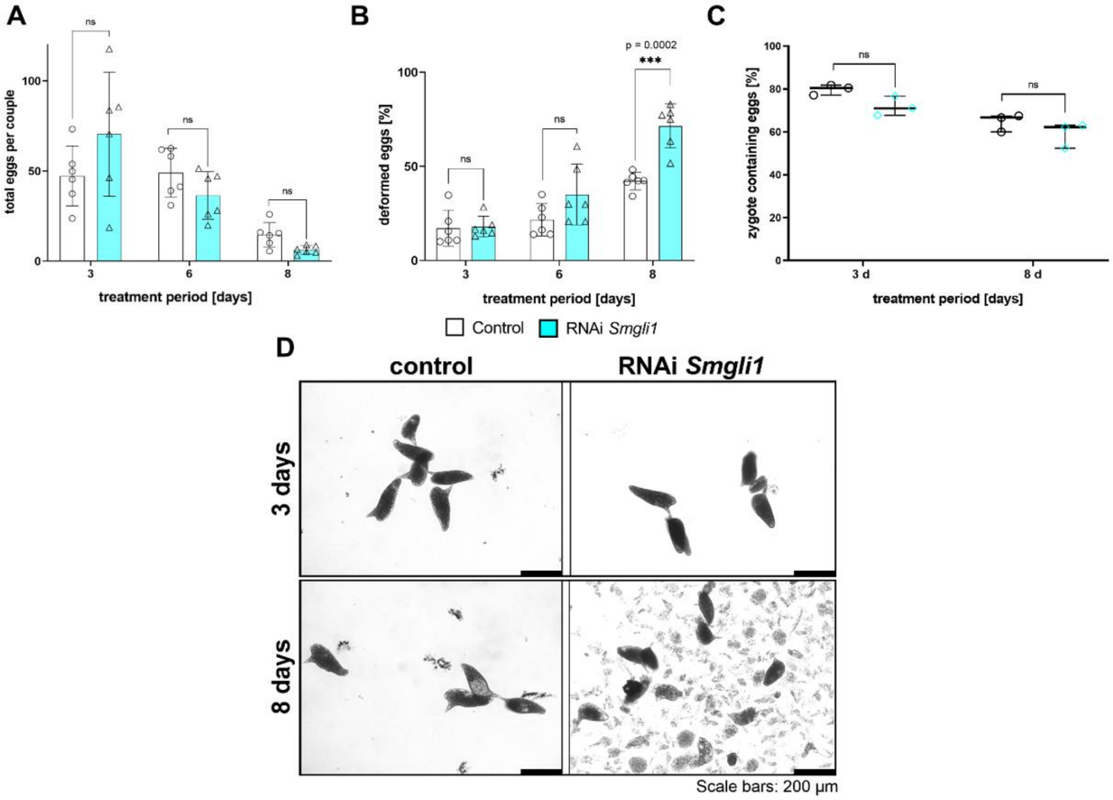

Suppl. Fig. S14: The number (**A**) and morphology (**B - D**) of eggs produced upon Sm*gli1* RNAi (cyan) were monitored in physiological assays and by bright-field microscopy. **A**, Compared to the control (white columns), no significant Sm*gli1* RNAi-dependent effects (blue columns) were observed on the number of eggs produced *in vitro*. **B**, After 8 days of treatment, a Sm*gli1* RNAi-dependent significant increase in the percentage of deformed eggs was observed. **C**, The number of zygote-containing eggs showed no significant differences between the experimental groups. **D**, Representative images of eggs produced *in vitro* after 3 and 8 days. Sm*gli1* RNAi caused severe morphological changes like reduced size, smaller or even missing spines. Furthermore, cell debris accumulated in the medium after 8 days of treatment. **P* < 0.05, ***P* < 0.01, ****P* < 0.001 by t-test (A, B; *n* = 6) and Mann-Whitney test (C; *n* = 3).

Supplemental Figure S15. Sm*ncor* RNAi showed no significant effects on egg production and morphology

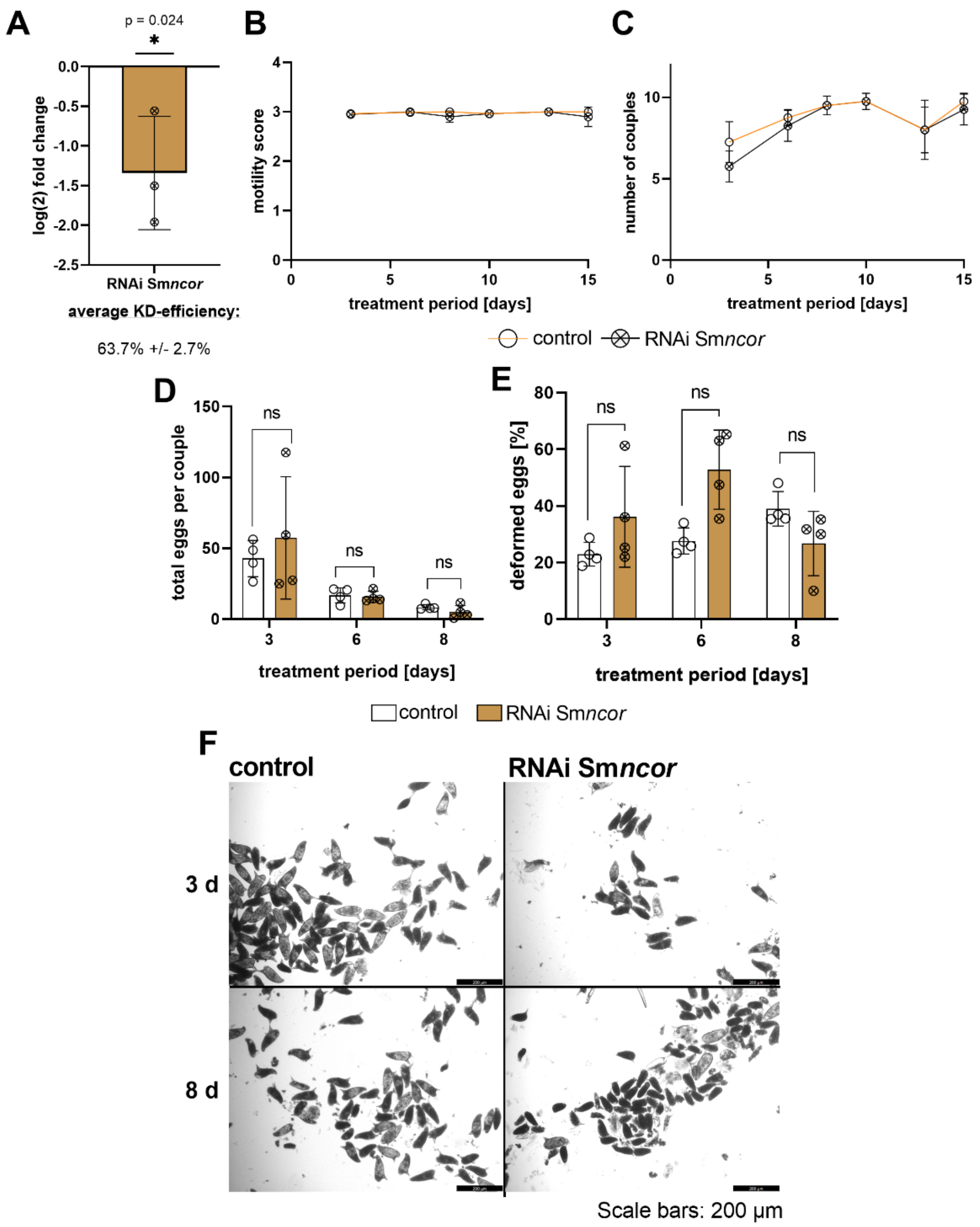

Suppl. Fig. S15: For RNAi, couples were treated with Sm*ncor* (brown, crossed circles) dsRNA at a concentration of 30 µg ml^{-1} each for 15 days (n = 4). Control couples (orange, circles) were treated with DEPC-water. RNAi efficiencies (**A**), motility (**B**), and pairing stability (**C**) were monitored as before. **A**, Transcript levels of genes following RNAi were determined by RT-qPCR showing significant reduction of Sm*ncor* transcripts (63.7% ± 2.7%; n=3). The graphs show average log(2) fold changes in transcript levels compared to the control and the standard deviation. Data from each biological replicate are indicated. **D-F**, The average number of eggs produced *in vitro*, and the average percentage of deformed eggs were determined during an 8-day RNAi period. We observed no reduction of the number of produced eggs (**E**) and no RNAi-dependent effects on the number of deformed eggs (**F**, **G**). *P < 0.05, **P < 0.01, ***P < 0.001 by t-test. A, n = 3; B-G, n = 4.

Supplemental Figure S16. Sm*ncor* RNAi affected zygote formation, oocyte proliferation, and oocyte maintenance

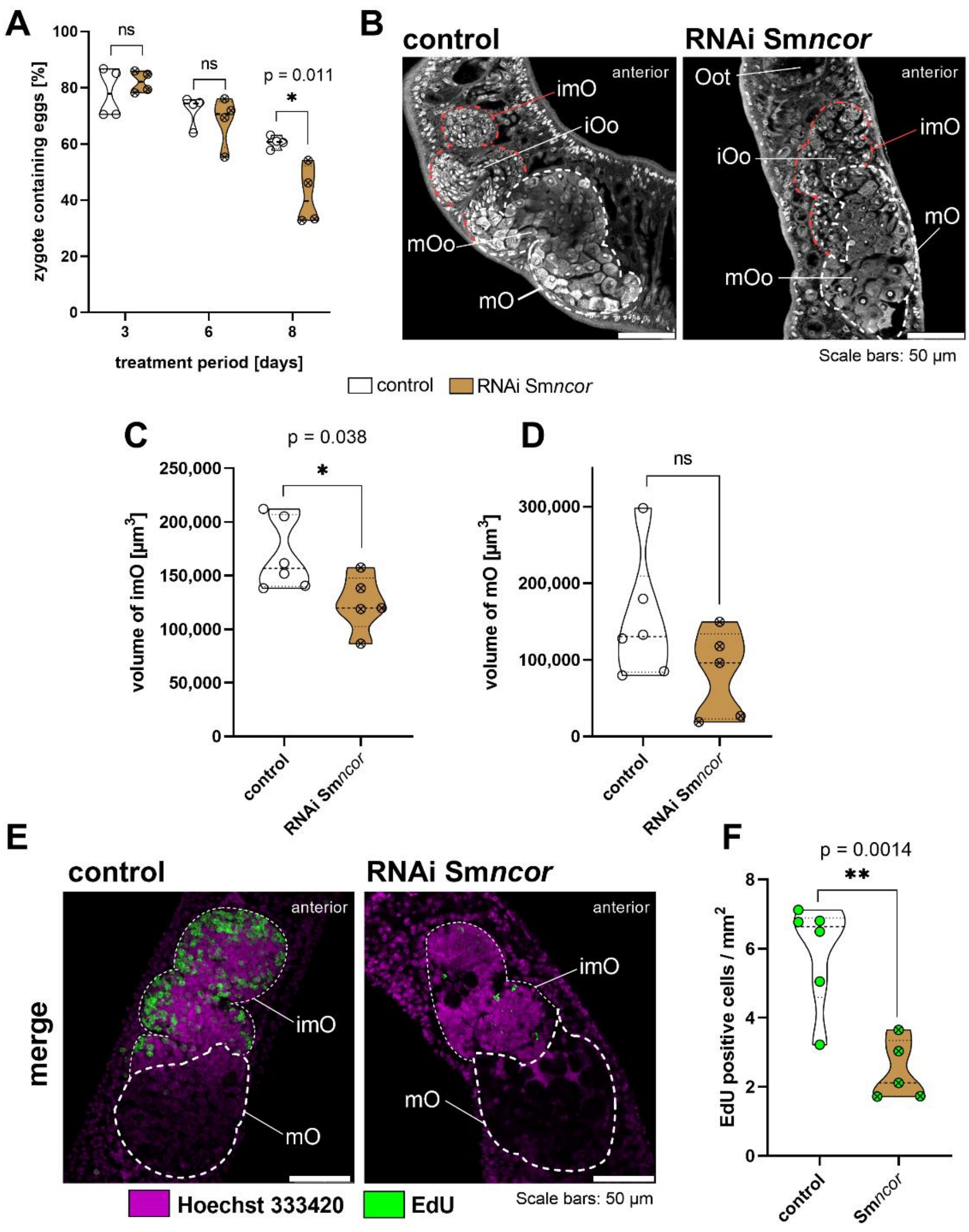

Suppl. Fig. S16: Schistosome couples were treated with 30 µg ml-1 Sm*ncor* dsRNA for 15 days. Subsequently, the number of zygote-containing eggs was determined (**A**) and the ovarian structure was analyzed (**B**). **A**, Weighted distribution (violin plot) of zygote-containing eggs after dsRNA treatment. After 8 days, a significant reduction in zygote-containing eggs was observed in Sm*ncor* dsRNA-treated couples (brown) compared to controls (white). **B**, CLSM analysis of paired worms showing a reduction in the size of the mature ovary of dsRNA-treated couples. Violin plot of the distribution in the volumes of the anterior (**C**) and posterior (**D**) parts of the ovaries based on comparative Z-stack analysis of carmine-red-stained females. The ovaries from the females of the four

biological replicates were examined; each point represents the volume of a single ovary. A significant reduction in the volume of the anterior part and tendencies in volume reduction of the posterior part of the ovary were observed in the RNAi group. **E**, CLSM analysis of EdU-treated female schistosomes showed a reduction in the abundance of stained cells in the RNAi group. In the control group, signals (green) were observed in the anterior part of the ovary, which contained immature oocytes (oogonia). The dimensions of these ovary sections were clearly reduced in females of the RNAi group. The cell nuclei were counterstained with Hoechst 33342 (purple). **F**, Violin plot of the distribution of proliferating cells in the ovary in the plane of maximum extension. A significant RNAi-dependent reduction in the number of proliferating cells was also observed. In the violin plots, the individual values of biological replicates are shown as well as their median values (solid lines). Abbreviations: iOo, oogonia; imO, immature part of the ovary; mOo, mature oocyte; mO, mature ovary; P, parenchyma; T, tegument; Vit, vitelline lobe; z, zygote. *P < 0.05, **P < 0.01, ***P < 0.001 by t-test. $n = 4$.

Supplemental Figure S17. Sm*meiob* and Sm*meiob*/Sm*rar* RNAi induced changes in egg morphology

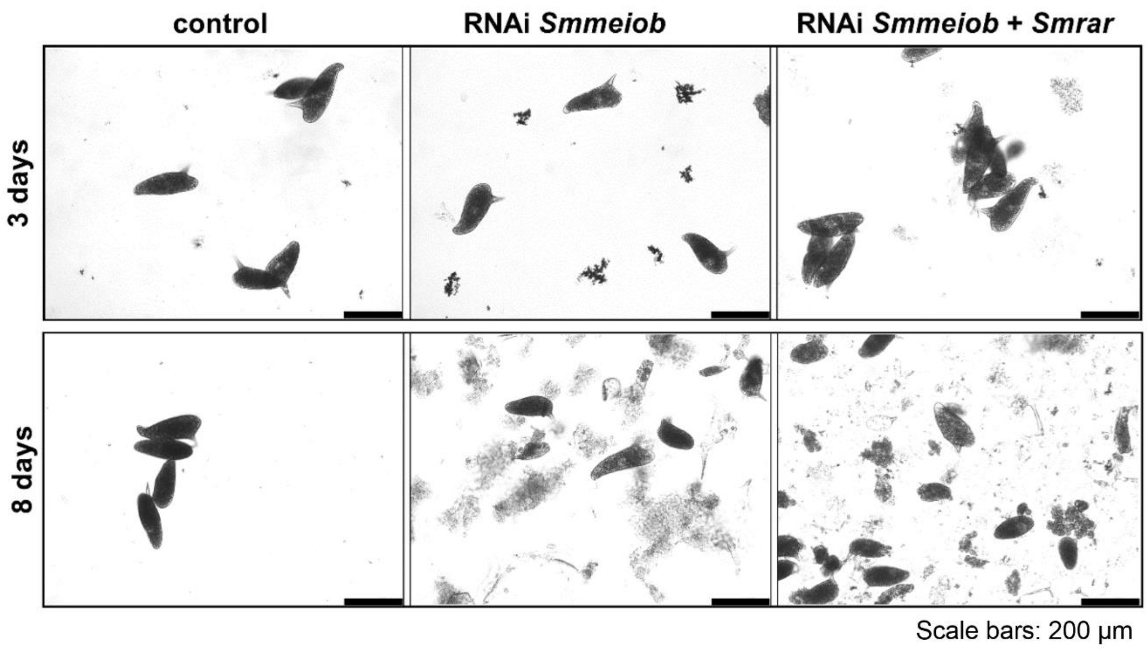

Suppl. Fig. S17: Representative images of eggs produced *in vitro* from couples treated with Sm*meiob* dsRNA or a combination of Sm*meio*b/Sm*rar* dsRNA after 3 and 8 days (*n* = 3). Eggs produced by the untreated control group showed no morphological changes. RNAi of Sm*meiob* and Sm*meiob*/Sm*rar* caused severe morphological changes, characterized by a high number of eggs of reduced size, which contained no zygote, and which lacked the typical spine. RNAi caused an accumulation of cellular debris in the medium.

Supplemental Figure S18. **Sm*rar* and Sm*meiob* RNAi showed no effect on the lipid content of the vitellarium of paired *S. mansoni* females**

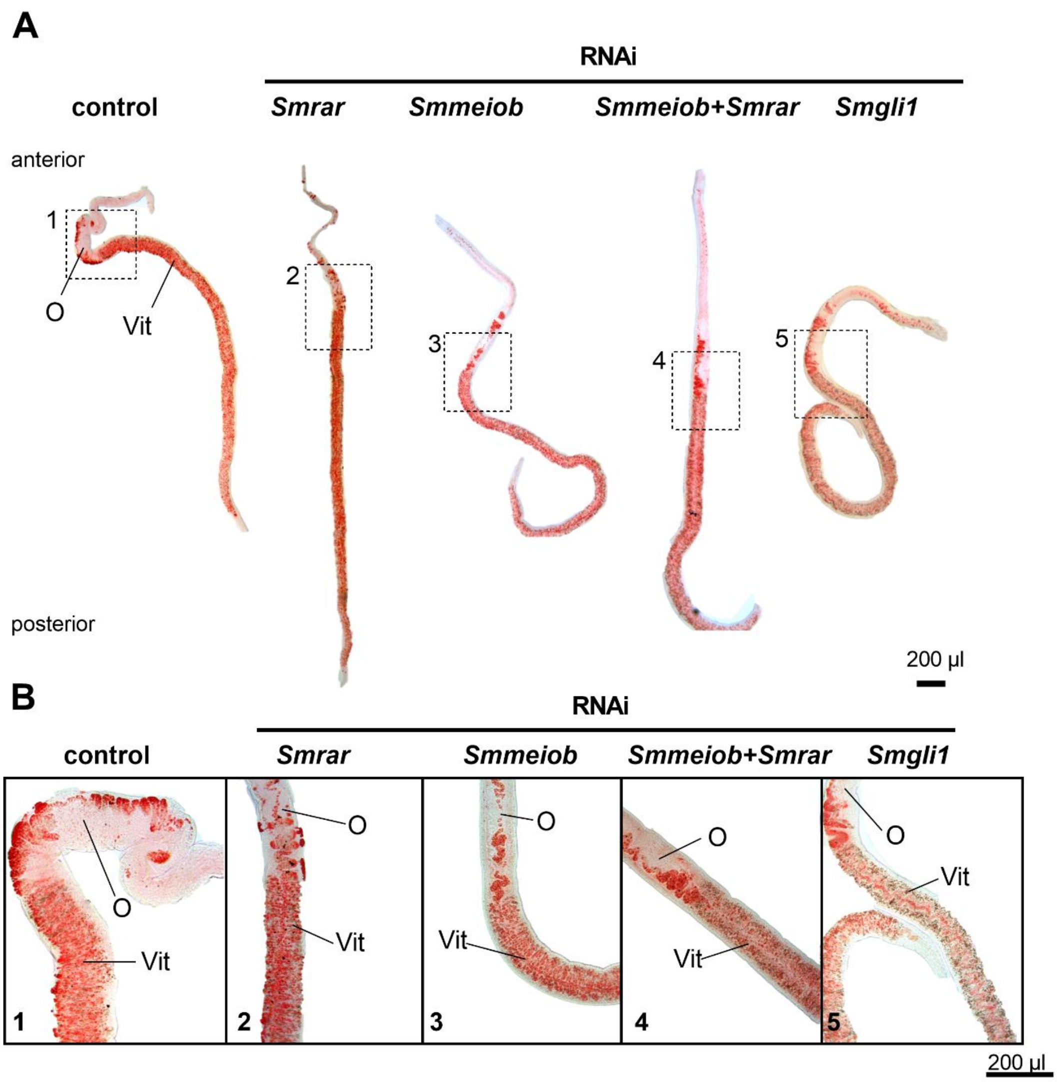

Suppl. Fig. S18: **A-B**, Summary of the results of lipid staining to investigate Sm*rar*, Sm*meiob* and Sm*gli1* RNAi-dependent effects on the differentiation of the vitellarium of sexually mature females. For this purpose, five couples of each biological replicate (*n* = 3) per experimental group were treated with dsRNA, as indicated. After 15 days of treatment, couples were separated, and the females stained with Oil-Red O. **A**, Representative image of a control female (no dsRNA treatment) stained with Oil-Red O. The boxes indicating the areas, which are shown in detail (**B**). **B**, Detailed visualization of the female reproductive organs. Lipid staining showed no Sm*rar* or Sm*meiob* RNAi-dependent effects on the amount of lipids in the vitellarium. In contrast, a weaker staining intensity was observed in worms treated by Sm*gli1* dsRNA, as expected due to previous studies (28). Abbreviations: O, ovary; Vit, vitellarium.

Supplemental Figure S19. RA promotes egg production in *S. mansoni* couples *in vitro*

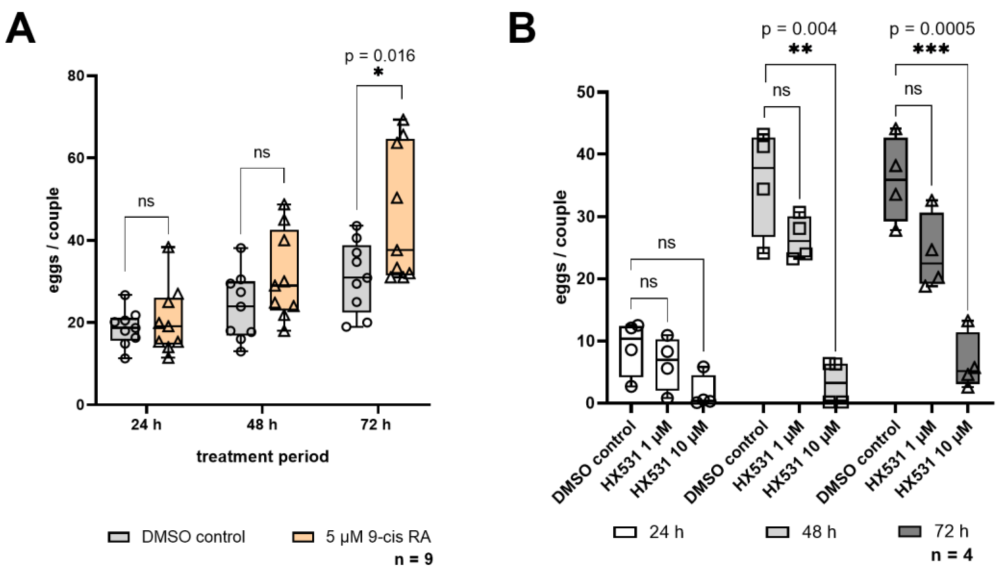

Suppl. Fig. S19: The potential influence of RA on the reproductive biology of *S. mansoni* was investigated by determining the dependence of egg production upon RA treatment of couples *in vitro* (**A**) or using the RXR antagonist HX531 (50) (**B**). **A,** *S. mansoni* couples were treated with 5 µM 9cis-RA (yellow, triangles). DMSO at the same concentration as in the treatment group served as control (grey, circles), with daily medium exchange and 9cis-RA renewal (*n* = 9). Upon 72 h of treatment, egg production was significantly increased. **B**, RXR dependent RA-signaling was inhibited by using HX531. *S. mansoni* couples were treated by 1 and 10 µM HX531. DMSO at the same concentration as in the treatment groups served as control, with daily medium exchange and HX531 renewal (*n* = 4). Upon 48 h (light grey, squares) and 72 h of treatment (dark grey, triangles), egg production was significantly decreased by HX531 at 10 µM. The boxplot indicates the range between the minimum and maximum values, with the 10th and 90th percentiles and median shown in the box. *P<0.05, **P<0.01, ***P<0.001, by t-test.

Supplemental Figure S20. Transcript levels of Sm*rar*, Sm*rxr*, Sm*vita-nr*, and HAT genes are highest in intermediate-stage oocytes

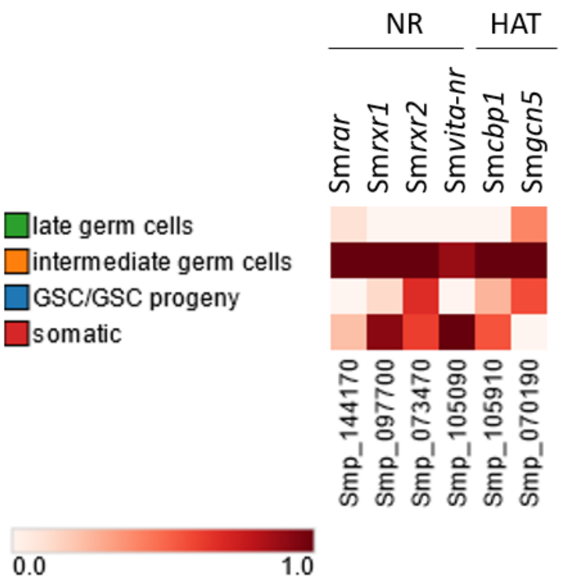

Suppl. Fig. S20: Heatmap of the oocyte scRNA-seq cluster-associated transcript amounts of the nuclear receptors (NRs) Sm*rar* (Smp_144170), potential retinoid-x nuclear receptors (RXR1/2, Smp_097700, Smp_073470), an annotated vitamin-A-activated nuclear receptor (Sm*vita-nr,* Smp_105090), and the SmRAR-associated histone acetyl transferases (HATs) Sm*cbp1* (Smp_105910) and Sm*gcn5* (Smp_070190) (21). All selected genes are co-transcribed in oocytes of the intermediate-stage cluster. The heatmap illustrates the feature-standardized transcript-levels for each cluster of selected genes.

Supplemental Figure S21. Protein structure modelling and alignment analysis of SmMEIOB

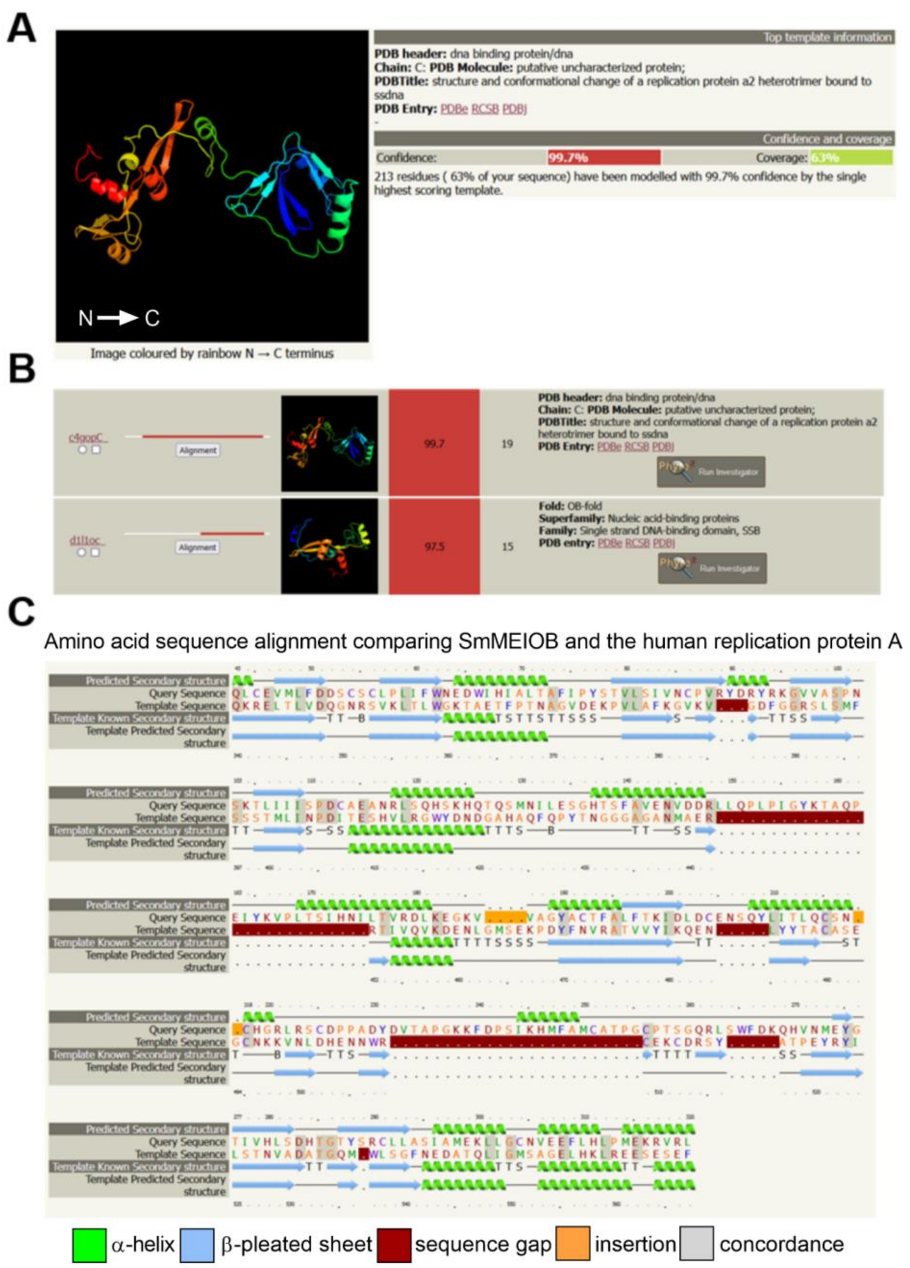

Suppl. Fig. S21: A, The protein structure of SmMEIOB according to Phyre2 analysis (https://doi.org/10.1038/nprot.2015.053) based on published structures of orthologous proteins in the protein data bank (PDBe). **B**, The amino acid sequence of SmMEIOB showed highest similarities to the human OB-domain containing replication protein A (RPA, DOI: 10.2210/pdb4gop/pdb, 10.2210/pdb1l1o/pdb). **C**, Phyre2-based results of the protein structure analysis of SmMEIOB in comparison to the human ortholog RPA. Using alignment coverage, SmMEIOB was predicted as an OB-domain-containing, single-strand DNA-binding protein, and highest similarity was assigned to proteins of the replication protein A family.

Supplemental Tables

Supplemental Table S1. Primer sequences of primers for cloning

Additional Document (Excel file)

Supplemental Table S2. Sequences of the *in vitro* transcription constructs for WISH-probes and dsRNA synthesis

Additional Document (Excel file)

Supplemental Table S3. Potential SmRAR interaction partners according to STRING

Protein interactions predicted by STRING interaction network for SmRAR

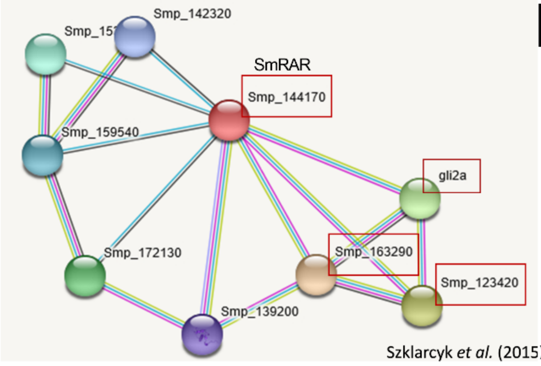

Szklarcyk *et al.* (2015)

Smp_number / name:	Annotation:
Smp_144170 / SmRAR	RA-related orphan receptor alpha; RAR-like nuclear receptor (Szklarcyk *et al.* 2015, Lu *et al.* 2016)
Smp_123420 / SmDarnt<br>Smp_341950 (genome V.10)	Arylhydrocarbon receptor nuclear translocator homolog (Darnt) (Szklarcyk *et al.* 2015)
Smp_163290 / SmNcor	Nuclear receptor co-repressor related protein (Ncor) (Szklarcyk *et al.* 2015)
Smp_266960 / SmGli1	Transcriptional activator *cubitus interrups*, Gli2a; SmGli1 (Lu *et al.* 2016; Chen *et al.* 2022)

Genes sharing a similar transcription pattern as *Smrar* (Lu *et al.* 2016; Lu *et al.* 2018)

Smp_number / name:	Annotation:
Smp_333540 / SmMeiob	Meiosis-specific with OB domain-containing protein (Lu *et al.* 2018, Wendt *et al.* 2020)
Smp_078720 / SmBmpg	Bone marrow proteoglycan (Lu *et al.* 2018, Wendt *et al.* 2020)

Supplemental Table S4. List of primers for RT-qPCR

Additional Document (Excel file)

Supplemental Table S5. Amino acid sequence data of SmRAR and its para- and orthologs

>Smp_144170 SmRAR [*Schistosoma mansoni*]

MSHCPVEYSNPNSQNTISYITSDSVISVSFADCQSSSPSYHSLSSSTALPGVGSFLDKSNHLPHYSPNKYYDTEPTAIPG
HSYVENTDVFDNNLPYPIKSEAIHETVSGTFITPDAHNESCFTSPLQPLKSLNPSLPVQSSESLLSGFTHPLSMDHTVLE
DGGLDLDRVFVAIINDRNSEHDIFNDNDVNCDSERCKKQLLNNSIQSSVNQSMLTDDSEDKSLLQYYSVIDSEKTTQ
HQTHPKTSPISEVEQTHKSSGSSEVSTRMCVVCGDKASGFHYGVSTCEGCKGFFRRAIQRDQSYTCAKNGTCEINKT
LRNKCQQCRLLKCIAVGMSRDAVRKRRQGKKREQSSCESVTLPSSDSSNKYEGSAYSSCSADVVLTPSKAHLNETTP
DPHEMPIKSINFQPSSSIPSSTNISLTLCNNNNNSNNSNNLTKEDQQTLDKFDRFLNYCKDQAIKYQANNWNISKSD
YYHLSGSLSRHLSPIKHTPHELGESLKLLTVDEIFCPAQLKFTHEFACHLEQFIRLSQHDQAILLRDCLPELAILMLCREN
RNKTHMNSSSTSINYQLNCLFSPWFPNAVVTDSSLDCLHMTCDSITAQSIFQFASRLQRLHLTNMEFGPLIGVILFTP
ERSDLLDIDFVNRTQNLWAELLRRYCESNGSQTRCAHLIMVLSTLRELASKITHNLNAWYKLSNTPMSNCLKEFLYSS
SFNSMDDCF

>Smp_097700 RXR (retinoid-x)-like NR [*Schistosoma mansoni*]

MIMSIFANHDAIHMNHGVSDESVLYTHSYLIPPINQLENDILKPDQDMLTCVQSNRSHSPSQQIYDMLDSTVLSDN
PIQTIANSSCKSQTSLKSSSCDTSNADLGVDIKPSFMYNDNPQVISQSFLTDLHNHTDIHTVSSDTNPNLCPIANSINS
THLQELVYNTPQKFPHMLPPDAHQHTVYSDNVVSEPFTRLLASGNSNETSTSYLPQVTKVETNSLTVSQSPILLFVDP
NKTKPESRECFPTQNPSELASQSSSATSVNTTNLNPICVICGDKASGKHYGVISCEGCKGFFKRTVRKQLVYVCRESG
QCPVDRRKRTRCQHCRFEQCLAKGMKKEAVQEERHRQPSSNPVPLISKPPKSEKKGPGRRSTFGNKSAESIVTDQP
PNINQDSTPNISITPTTTDCVQPNQVKSESSTTCIQSNNVLLSDETDLPNLTLRCLLSAELSMDPKLAVSERGEAIYEDI
PGDDDTGLHPLTIICQSIEQQLPRIVNWARQLPVFSSVYLSFDDQFCLIKAAWPELVLISSAYHSTVIRDGLLLSIGRHL
GREVAKSHGLGPLVDRILHELVARFRDLSLQRTELALLRAIILFNPDANGLSSRHRVEAVREQLYSALHSYCTTNQPQ
DTSRFTKLLLRLPPLRSIASKCLEHLVFVKLAAEDPTSCRLINLVEHGVWPIQEKSFELATLPSDSASTDSVPSQITMVPT
PQYIQHQDDNSLPTSSHTDLSYTHTLPNFHTVQNYPF

>Smp_073470 RXR (retinoid-x)-like NR [*Schistosoma mansoni*]

MIPVSIVTPQSVSSAEQIGEHSSSLSLSLQNVPVQQDSVSYLNSHTTVHMNDGQNEPSSIYLTDQELRLDSSFPNSPLGI
TRDPHCDDNGEEQNRISSFELISRLIDAEGLIELGYIPSSCNSAPAIDVASLSDLEEVTSKTLLWSSNCDDVDSFNFDDL
SNKINESNTNNADTKLRSDNKTLEHRSALTTEYISVDFNPLNSTNALVPIISLPSDTNLQTIPSLESNEYENFQAHYKTN
TVLENVQSLPSNTVPDDLGPLNITSYPMGLVEADIPSITRPSIHQSPVDHHALHPTIKPNHIPYSYSPLTPLSSVQIRSSI
HESSFVIDNQVCPIDNICNQQLQTGSFVKNKYSSDYQEYSGAPECFPTVHTLELFTSHSSDLPHSSSHPSKNVGESPK
HLSNDTKPIKYPELLQKIGENCNSNISGPNLPFISHSTYCSRTQSSSSCSIRQQLATAPSHPQLHVSIPQSPQSIYSVFSC
SPNQKVAVSSTVNLPSSDSTSATLIFNGDFPSRPIISSTDNSFWSHPSIPSGSAVNTQMITNWEHSVPYPAYVPNSQI
TGFLPENISVTDKAAIYVKDGNSTAIQPVLGSGLVGSFQYLTPTTVLSKPNYTILNDQLTFNHPQETLIQPFLPQMSDP
IKTTQNLRIPFCQGTQYTLTPGISIPINPEHSFGRLEMNTHRYSSESSKTNPALTSRSSSSRPFNSPVRRNSAPFQSTYQ
NCMNFSETAAHQSECSIVVSQLSTKKLTTPVLCCISSNSNNIPIISQQSPNNNNNNNNNNNVVFKTDNTKQHIIDQN
ILTKTATTTSIVQPSELCHIQPILISSPVKNNNNNTHNLGTGSYIDSNCLSNQSNNDKSHSITPSSPLLTNCNSPSTTLQS
STVCCLLRPSGRSTSTSSGSSCGSSCSGTGGGVSVSSGQYICSICSDRASGKHYGVFSCEGCKGFFKRTVRKELTYICRD
SQECQIDKRLRNRCQYCRYQKCLRAGMRREAVQEERQQQQLQSEVQRSPTPPEQNCDLSVNSMIMSDTKITNAM
NHSCLAEKQEIMLKTTNCTSSSSPHLLSNCSDSSINYFYSASNEKSQQPSINDNFNLTVNDAATYPPQELSLIGNKDSN
NVTLPLADIHALKLPTTTSAAIPPPPDALEFIRTAESTISSRRKQWLSAFNKQQCHAEIAKCFQDSMENLKWLENNFE
KCTTNHLPLFDLVIWSSKLPYICQLSCGVHLDLLKSACMQLIIVNLVYWLANDHKPRSLSTSNSTSKLPDTTPTINSTDI
SNITDDPPENSISDISKDCTIQMKKINKSVPLDEKMDYYYSNFPEFHLLNNLTKPMDNNNNDSISSKPTNINDNSVD
DDMIRKRNTNVYKLIYNLAIKLRMLNLDPVELGCLKLILLLNPDSMTCLNNIRSLIELLRDQVYAGLEYYCNQVWPNA
PHGRMGRLLLKLSNFQSVAARIEKLICSNELNNLLNNLESIFSYLSKKKVDHSNYISTTTTTTTTMSSTTTSTSNSIHLEF
S

>Smp_105090 SmTR4 (vitamin-A-activated NR) [*Schistosoma mansoni*]

MYLHSPITTITSTDSSSTSTITTNTNSTSSLTVTSSPIDPSKLDIMNTMSNPTCCIPSNNIIQPPPIIPNHGLTSLLSNVAS
NLSAHQNHQNNFLKPSLVPTGRTSNAQLLQLLRNVTSSLPSSPVSYPISPRNTRNTPVLCDNNHTLSDSTSQVPIIST
ASSTAIPATDYSSCNNDQTLTLNSQSNRTSSNHPTVNGPSLLPDFETLLANSNPNMSDFIYCLVQSILFNHANTRQKF
KSKKETPPLDSAALLSLLSGAGNLNPSSCSTGSLSNPLNPLSTLSSEIDSANCNIHLRQVLESLISLSVSPAFDQSSPHNL
LSNDMLDSGSLSQTFQQLTNLSNRKATTGIQKVSPSGQILSNESSNSTNPPGNVSFDLLAQLSAAAAAAAVTPPPSN
IVNQLGNLATDGFNPIFPTDENLSSALQNLLFKQMLSINTGSNAYTSHLGPGRDSVSSISQSGQYTGLKDCRSMSND
ESNCSSEKLIPTTYSLASSFLSLHSPNSTNNCSNNNNPRSSFVRPSSRISPEPITLASSPPSSSSTPSFICPNQSNPPTGEF
YPPNETVASSPNSLLLNGCTPVGTSPNTGLNMNQTAVNLSNDNQSWEPCKVCGDKASGRHYGVVSCEGCKGFFK
RSIRGHVSYVCRSEQNCLVNKAYRNRCQYCRLQKCLAVGMRSEAVQNERRPTNTFALNFLNDNSGNSNCTTTGGS
SNMNNGGSPSPSNNCCVSTPSITSTTTTTTTATVTTPTTTSANNSHTSTSGPSVTNVRNSSPQSQQLPYKIEPNSEEL
EDDRNNHSEFTTQKSEEGQSSSHSSPSGSSRSVPLTLSGATTVGLLSSSSGGVGSKHLDDNHFTGINKHSALSDISSSS
SSSSYSATIADVEINDPNNASYLQHDIKPPVIQTSLGLKFTPGLCSSPQATSHSLQQPPAICLSTSASSFTRQRSLTSVSS
SIVNNNSTAYASALPAYSNMLEGCHTTNHLDPDCGIANFSRCMNRNPLLSSQLFDIPDNKRGATQESYSRRMTSLS
NTPCSSSATVSNHQDPLSIFDDTGGLNLNELTKLALNNINSLQSSSSASNQQGRSLAALYTYWLAAAAAAGSLNPLH
HRHNDNHHHHQQQNHSVHLSTSNQNFNPLDMKTSLSSLKAGQPSTVTTTSIVGSNQNSTSQFSMKTNPNGLFG
QHCNLENQLLINGLNHSSKSSLLSSSSSHSDALNMLMSIMLNSSDNVGLNNFNTLTDAVNSTTTHLQKAPINHHSH
QHHPFMLQHHPHQHQLHMGSSNSHHSSTSPNTLEIPIPSTSSSCLSASLFGLNNTISTNNNTNNNNSNDNNSTMH
TTVANNNSPFIPAPPPIKSTNLIQLLCKRTSNSQTSTLTDTDDKSKSGSCGSMVASTTNVRGRRSSNSSTANNMGEN
LHESGELIDSYRDANGHLLPKKFELTSSPDVTALRPENELLNPSPKKEETNWSRSRKRKMHYSFPSDVNNNARSIRRR
TQSNNSLQPDTNNSIGLGDTRETCEDDDESRITECISECTKSPDDSRPNSPLVGPVLLNSMFDLNAHVFKAYQDNN
NNRSLSISSELASRVLFLTVDWLRRFDGLKRLPIGVQRDLVAISWSDLFVLGLCQAADQINRSQNHRSNQESNDPQS
TNSLCAQNDINSTRVKQISGEQQTNSHCSITPTSSFKSNSFDSSSSSHISPMKEPMDTSYLKTNCSSSSAVIELVEQLM
KQFSGAEVDTHEYTYLRCMVILSSGRLCINARDASLAKQITEMESRVLSEFSEFLSTRAASSTTTGSLSSKRTKPIIKRVLI
LTQLLSTLRYLDPKDLEEAFFSNLLGSVSIAQILPYLLESNDLFTQSQLVMNSCLPDNSLDYKPGLNHVKLPNKQASRS
GLEDSVTNFMGDSLPNNMTEKIDNLPSSLLLRPDQTSPQSRSASCEITATSIELNNQNTEPANSKSFVDDETNDSA

>Smp_036500 Smnhr-48 [*Schistosoma mansoni*]

MDPGANDYPSGNVNANLRSNSFRISSGTPLDNSMNINGPSSVGYGSNTGHDKESFLCLNRTQTDYVQNHQYIQSV
HNDNTNNSHYCSNFEFSQSGSRKHPLSGVVRTRRRKAGISEEERVCIVCGEPASGYNFDRLTCESCKAFFRRNALKP
RDKIKACNRGGGCAIEGNQRKHCPSCRLEKCLAVGMKRELILPPEKLEQRAKPRRRKHHSPQDSSSSPQNLLTGPSS
VGSSNTNAYPPYLPSPLTTSSHPQHGSNQTSYNSSFVSGASRPLHLINPVKFSSYVNSSHCSDLSLTTDTFNSSSTLETK
ECNFPNSSIKIPVIGKSDELFMPPVNQDQNLNEKFLSLESSQQSDSFCKTRHGLCLIQCRKSGGRTFCQHRSCRLRSA
RKRRLRRRCTSSGSKCFRSPLMHITSPDPHLLSCLQGCVHRIREPFTPDEQLETGIAVTLEQEYNRMDACIRRIIRVVK
HLPYFSEVGKPAQLSLLRTNIYGLIVLYSSFFFERDIRKLRYPVLQTDGQLTTVTVSMLDEVATPSTSVNDPDNVISGSY
PKLLQSNSFRSFSRCRSNQANYAMGLREEFELYKSNTVAAFDHLDELVGSDDILRMSVLAIKLFSDNSLSEDLRPPVF
AARQAYTLFLWNYIRWQAGSKRLRSATELYARILIAFIDLRTLEIRMTEFAKLLSLDGLSPLMREVCSQRSNLGSTSA

>RAR-like NR [*Schistosoma rodhaini*]

MSHCPVEYSNPNSQNTISYITSDSVISVSFADCQSSSPSYHSLSSSTALPGVGSFLDKSNHLPHYSPNKYYDTEPTAIPG
HSYVENTDVFDNNLPYPIKSEAIHEIVSGTFITPDAHNESCFTSPLQPLKSLNPSLPVQSSESLLSGFTHPLSMNHTVLE
DGGLDLDRVFVAIINDRNSEHDIFNDNDVNCDSERCKKQLLNNSIQSSVNQSMLTDDSEDKSLLQYYSVIDSEKTTQ
HQTHPKTSPVSEVEQTHKSSGSSEVSTRMCVVCGDKASGFHYGVSTCEGCKGFFRRAIQRDQSYTCAKNGTCEINK
TLRNKCQQCRLLKCIAVGMSRDAVRKRRQGKKREQSSCESVTLPSSDSSNKYEGSAYSSCSANVVLTPSKARLNQTT
PDPHEMPIKSINFQPSSSIPSSTNISLTLCSNNNSNNSNNLTKEDQQTLDKFDRFLNYCKDQAIKYQANNWNISKSDY
YHLSGSLSRHLSPIKHTPHELGESLKLLTVDEIFCPAQLKFTHEFACHLEQFIRLSQHDQAILLRDCLPELAILMLCRENR
NKTHLNSSSTSMNYQLNCLFSPWFPNAVVTDSSLDCLHMTCDSITAQSIFQFASRLQRLHLTNMEFGPLIGVILFTPE

RSDLLDIDFVNRTQNLWAELLRRYCESNGSQTRCAHLIMILSTLRELASKITHNLNAWYKLSNTPMSNCLKEFLYSSSF
NSMDDCF

>RAR-like NR [*Schistosoma japonicum*]

MSNCPVEHLAPNCQNPISYIASDSVISVSFADCQSSSPSYHSLSSTTTLPGVGSFLDKSNNLPHYSPNKYYDTETNPLS
KHPYVEETGFFDNSHPFPTKSECIQDLVSGTFITPATHSHSCFTSSLQPPKSLVPSFPVQSNETALNGSTHLLSVDHTIL
EDGGLDLDRIFVAIIGDQSSDHHVFNDADDVNDNGGNCLVDKSNSCNTQLLNNSMQSSVSQSMLTDDSGDKSLL
HYYSVVEPEKTTQYQIHSETPVTEVEQPHKLSGSSEAPVRMCVVCGDKASGFHYGVSTCEGCKGFFRRAIQRDQSYT
CAKNGTCEINKTLRNKCQQCRLLKCIAVGMSRDAVRKRRHGKKRQQSSCSDSAALPSSDSSSKYETATYSSNSTDVF
LTPSKTHSNDEITLNSYEMPIKSSNLQSSASSSLPPTTNPTLTLSNSSLTKEDQQTLDKFDHFLGYCKDQALKYQTNN
WNISKSDHYSLSGSLSRHLSPIKHNSHELGESLLLTVDEIFCPAQLKFTHEFACHLEQFTRLSQHDQAILLRDCLPELAIL
MLCLENRNKVPMNSSSTFSSINHQLNCLFSPWFPNAVVTDSSLDCLHMTCNSITAQSIFQFASRLQRLHLTNMELG
PLIGVILFTPERSDLLDIDFVNRTQNLWAELLRRYCESNGSQTRCAHLIMILSTLRELASKITHNLSGWYKLSQTPISNC
LKEFLYSSGFNSADDCF

>RAR-like NR [*Schistosoma haematobium*]

MNPPNIEAAHIDLPIDVNPSTTEEIRMAIRQIKNGKAAGPDNIPAEALKSDIKATTSMLYLLFKKIWGEEQVPMDWK
EGHLVKIPKKGYLSKCENYRGITLLSIPGKVFNRVLLNRMKDAVYAQLRDQQAGFRKDWSCTDQIATLRIIVEQSVE
WNSSLYIKFIDYEKAFDSVDGRTLWKLLRHYGVLEKIVNIIRNSYDGPQCKVVHGGQLTDAFQVRTGVRQGCLLSPF
LFLLVVDWITRTSTSEGKHGIQWTAQNQLDDLDFADDLALLSHTHEQIQMKKTSVAAVSASVGLNTNEKARSSNTT
QRTPSNTITLNGETLEDVESFTYLGIIFDEPGGSDADTKATIGKMSHCPVEYSNPNSQNTISYITSDSVISVSFADCQSS
SPSYHSLSSSTTLPGVGSFLDKSNHLSHYSPNKYYDTEPTVIPGHSYVENTGVFDNNLPYPIKSEAIHEIVSGTFITPDA
HNQSCFTSSQPLKSLNPSLPVQSSESLLTGFTHPLSMDHTVLEDGGLDLDRVFVAIINDRNSEHDIFNDTDVNCDSER
CSIDKSNSCNKQLLNNSIQSSVNQSMLTDDSEDKSLLQYYPVVDTEKTTQHQTHPKTSPISEVDQTRKSSGSSEVSTR
MCVVCGDKASGFHYGVSTCEGCKGFFRRAIQRDQSYTCAKNGTCEINKTLRNKCQQCRLLKCIAVGMSRDAVRKR
RHGKKREQSSCESVTLPSSDSSNKYETSAYSSGSADVVLTPSEAHLNETTLKPHEMPITSTNFQPSSSSIPSSTNISLTLC
NNNNSDNSNGLTKEDQQTLDKFDRFLNYCKDQAIKYQANSWNISKSDYYHLSGSLSRHLSPIKHTPYELGESLKLLTV
DEIFCPAQLKFTHEFACHLEQFIRLSQHDQSILLRDCLPELAILMLCRENRNKTHMNFSSTSVNYQLNCLFSPWFPNA
VVTDSSLDCLHMTCDSITSQSIFQFASRLQRLHLTNMEFGPLIGVILFTPERSDLLDIDFVNRTQNLWAELLRRYCESN
GSQTRCAHLIMILSTLRELASKITHNLNAWYKLSNTPMSNCLKEFLYSSSFNSMDDCF

>RXR (retinoid-x)-like NR [*Fasciola hepatica*]

MNINILKSEPDCVDPSLNEDSGLAAAYDLVLEHGDSFGDLTPESKTDLSRSDSFSFDEPRLVLPPVAQTFLRQDSAMP
SSTLHYGVASPTTTSGSAGGVLPMQVCITDTHDSGKQPFEEIIGEHDHLPVVQSQWKFGGSGATVNCETPITNSVQ
LPTLSSTIQLSSDNSLKIESNPQTHSQVTTPTSNRKLNSGTSDSRRLAVAQSPAGTPYPSLTQAPMPSFSNLPLALCAIC
GDTASGRHYGVISCEGCKGFFKRAVRKQIQFTCRGSGQCPVDRSKRTRCQHCRLEQCLAKGMRREAVQEERHRYF
QKTPKSRSKSRKADPLGFQPDLLLSMDSVSTELNGHTPKSIEHDSTVTTADPLQSSPTTVLCSGRKQNRANQPGRFA
TTSGTATGELMSASTAPPPLSLASLLTAELSTDAELPTTATGERVYVDIGDDGLDPLVVVCQSVEEQLTRLVCWARQL
PVFTIPYFSTEDQLWLLRAAWAELLLISASFNSIAVRDGLLLANGRHLSRNKARQHGLGPLMDRFLLELVSRFREMSL
ERIELALLRAIILFNPDANNLCARQHVESVRESLYAGLHSYCTTTHPNDTSRFTKLLLRLPPLRSIAQKCLEHLVFVKLAA
EDPSARRLINLVEHGVWPNDDRAFCPQQSQQQQIQHHHQQLQVKQTSPVGLHPQSAGPSTSIGPGSSGTVTRFS
WPSQGIHNHPVPGTLLPHPMTNTNNNNSNHSHYNSTSGVPTTNTESAPTNGKANIKVDLDSLSQPIADNCSVE

>SmRAR-ortholog [*Dicrocoelium dendriticum*]

MNSHSDLLCFENASPSGHEFWNITKGAIETSSSNALGSDCSYHYPSLTPATEFLNDSIGTSHHVTTPCSISTPDHSTTT
SEHVTYHQLQRLTKTSGMSIHAPSELSESLRLGLDCTENYLDINDHSQVPIYPQNHKDSLYIPTSNYQHSNSASPSSAP
KEILYDLSHTHQLREVFHQPPYHPQASDLPTEGESICLDLDQVFSAIVSPEDESDVDPVKFNENASNSVDFHLKTCIPP
AGDSIFPDDILFPLNSAQDAPRSSDLNPSTLPCIVAGDESLSVTHYPNCPQPLRILCDDREKDVYQRPLLTASHADSSS

FPLENRPGFPLLNSSPVSPFSRQILSRSPRFCSAGPEQLSFSLSPRSALYSSSPMRSKHNSSCSMNTDGSVMTPQPFIH
MSSVSSSPRFHQRSPSHLPWPHIQASITDARRMELAPIIPNLSPNRYLFGNTTRLTASIAEIPVDSTHMDSTVHHAAR
GSQPLGANVPKESLAHIRPRGAQSVSNPTIRFCEVCGDKSSGAHYGVYTCEGCKGFFRRAVQRNRTFSCARNGQCE
VNRVLRNKCQHCRLHKCLASGMSKDSVRKKFDSDEKVSPLRRAKVRPTNARTIRKGGPIGVGFQITNPSDLHISRDN
DPLPVPQHTTPERPQAPLTPSERPSFTSPTIMPPLSIDDRRMITSLFELFHASRKQAIIESGGEGHHVGAKGELTVIGS
VEQLFCSAQLQFAHYFASCLGEFNQLSQHDQAVLLRGALVEITFLLLCNNHRCADAPPSIPTIHSGSVHSTSRCSYLLS
PWNKSLILTEESFEHLHLSDNAWTPSRIFQFAERLTQMRLTSDEFGPLLGIVLFTPERANVLDIGAVNQIQGTWAELL
RRLCESQGSYTRCAQLIMLLATVRELSGRLAHNLARWYRSRGAPFTECLQEFLLPVLSELVYL

>SmRAR-ortholog [*Opisthorchis viverrine*]

MLFVAFRLMSSPSDTAVLQSAISSKADWNYNSSSTNEIQPRAITSFESYLSDVEPVFDHQLSCYTPAIPSCTGSHLSKD
TSDARQNQGCVTGEDLQQEYIPQSIVTNCSLLPTADSINYLPYYLSDRSIGSSVQSPTDTTSLNSRSPTHVTRVASTSD
MILPSEPLVLSEGSVLSQSTVAPDESRSLDLDRVFSAIINPVSDIHFNPASLAVDPRNPVNHCSSLSSFVSSSGNSNVSE
NVAGSMNLERRRHLSGFPDSSFSSALSDASESLNSNSVDYVCESSHCRDIRPSFYTKAGTSGAHAPSSLCTIPSEGLSS
SCPGTFDRSLHSAPDSLISTRSILSSCDSQINGVIQHQSNMLDTYSRSDRSVKNNHFTGVDPFTFSPSNESHSWNYQ
QASTTSCNKFPFATEFQLKRQEFSVDRPAPLSAQSESPFAADTRSSTENVGQTAQAGVLLRACVVCGDKSTGAHYG
VFTCEGCKSRAATVQKSEQPYIRTSTLVTDKSRSVQSCTSTCTHIHLAVSALDTVRSLPLTAIWMLSSFILRHRSEMAQ
WLEREFAGWKLDTERVLQLNDFFISLDVLVKAPFPAPCTGWRRHKRDRPTTSLTAVTSEADFEFTRSMKEVTKRLG
AVGATRLPGWGPRDHHCAWLETLQDMTANRCQWRSCCQFLSRLPELSNKSWLYGSEASMLNTDTYACARNGSC
EVNRALRNKCQHCRFLKCLASGMSKDAVRRKQPAGNKKSSKTTGRRRSNKSAHTSSEHPEPMLPCDVNRSDACSS
QTTPRSVYSGTEQSTVGWTAGSSISFPSALTNPLGKLSPTTNFLSPQDRQIIVSLHDLVRASKKHSLAETRMQMNSCL
PESDGKIVITSVEQILCPAQLCFAYQFSGCLAEFAQLSQHDQAILIRGCLMELTFLLMCNNYRLAPEGESALCTGDGTE
ISTSEGECSAYLVSPWNANFLITEASFSHLHLTDNTWTPSRILKFAWRLTQLRLTDEEIGPLLGLVLFTPERADLLEVQA
VSRIQGVWAELLRRLCE

>SMED-HNF4 [*Schmidtea mediterranea*]

MTSTQHPNLNPAFSYQLLTSNSAPISISSNASSMQYPLESEMSSQQHEVYASNASGPEGMENNQLCLICSDKATGK
HYGAFSCDGCKGFFRRSVRKKNNYTCQYNRNCKMDKDKRNQCRYCRLKKCILVGMKRAAVQNERDRISTRRSSFD
DIPPNVILSISQLMQAEQRVAIQKPPNPQEYMNRYADVPDVCESMKNQLFLLVNWAKSLPCFSQLNLSDQISLLKA
HAGEILILGVIRRSFQLDEDDVLLLGNNLIISRNSSDKHFAEIASHILDDLFIPLRELQLDDAEFACLKAIVFFDPRVSESG
KEFVRRCRYQIQMDLMNHMNDKQYHKPGRFGELLLTIPDLRLVTQLMVKKVEFMKMTGLAEIDSLLSETLLGDNP
PGVFSIDPETDGNNNNNQNDSMDIINSHRISSPKYTYQDCYGIPPFMPDSVLLNINNQNISPEAASKIWSAYLPSSTS
MPSGNIYRAFVNQGTEIMQQNSQDNSNFGMIINSDGMMLKNERVGVPSLFSCVNDSINLYQHSNLSQDTSHFLV
NLDQGFPMINQSKYAYNGDANHNCSNSSNSDDLIHPALSVNSTQNVVDPSLLVAYTKSLPNSPIENCQIQSIANRSS
EANQQHNSRSNYSAISSTAHFLTDHPIRIQESESSVFKKEEY

>SMED-TLX-1 [*Schmidtea mediterranea*]

MILSTGRILLDVPCKVCQDHSSGKHYGIYACDGCAGFFKRSIRHSRLYICKNKSIKGDSWIGICKIDKTHRNQCRACRL
QKCVDSGMNKEAVQHERGPRSSTVRKKVAMYFNELSHNILDPSSMLPFTKSFNNANNVKEELLEFSLISAAKLNNF
SNKNSFSISSLCDNSENSIETFDDCIGTPKSTIYFFQESYFSELSARSLFNTVHWIKSLQLPKEIVSVLLEGNWSQLFLLTA
FETKVPFNDRSLMYRICDQVSSLSTMKQLDLINQCAVLHQHLSKLSLGSSEINILKQIIFLDLKSLNKKLEVAVQDACM
DHVSRLCQLFDPTNKIAIEACLLMNIICIDPNFIHKLFFQKTIGQIPVLNLISDMIA

>RXRb [*Echinococcus granulosus*]

MQVWSNSNNASSVAPPANGDRDPMHSMPVTAESVPPTLNAFDSSIFASLISESDEKPLVDLKSDVISNADLQIGSA
MPKPDLGSLYGGLQSSTQTNNTCDVATTHRTVFPANPQLQTLPCQSSCVSGSLNYPYTPTLYSPVTPVSPQYFQSAP
PTVANHRALPHQQQGSYSLPPLRSYSYNQSFITGAYPKPYNYTIGSDGSISPYHRSVPSTSVKKVTYLCSARNCAICGA
TATGKHYGALSCDSCRAFFGTATHLGLRCECSGKSNIHDNNHQLRCHSCRLRKCLAVGMRKEAVRFEKSHAMLLYP

NEKDYSSVAPSPASSICSHERADNLVGSRSSPATEATVKQIRAAEALIFDVPPLDLPESKPIFMDGKDCDSLQTDIQESI
INLLLWSQKIPLFSDFSDSDRFILLRAGCIELLLVHFISRLANSLTESHSSTSLRSSNPPLQESSPSTSVTPIESTTTPPTPLN
IPVVVVDVLFPFHSAPKDILFSQQAECDLRNADRWHESRVLMNILPVDVDHHPISITDVPNELNAQCLSRRLILCRLF
DLARLFKRLCLSVEAVGCLRMVILFNPDVPELTEATRERVESRRDEAFICLEHTFVKADKKTALGRMAQMCLHLADLS
FVAERIYSKTSSHPYPSFQCLVDLLECFYKSDPMATVSESST

>RAR-like NHR-23 [*Caenorhabditis elegans*]

MACKVTGPNRHLTSVLEMSSFVYWPRSRQQAHNFPMQAVEQKLADATRTLHAKSSLQPSLSIETPKSKENDESGC
ESSNCMFHPHTIKSEPNFCFAREFKSVPDDFRIGGGDLQMGNNSKRLTCVIDTNRVDMAGILPDNMSFRGLPENK
SLLVSAQIEVIPCKVCGDKSSGVHYGVITCEGCKGFFRRSQSSIVNYQCPRQKNCVVDRVNRNRCQYCRLKKCIELG
MSRDAVKFGRMSKKQREKVEDEVRMHKELAANGLGYQAIYGDYSPPPSHPSYCFDQSMYGHYPSGTSTPVNGYSI
AVAATPTTPMPQNMYGATPSSTNGTQYVAHQATGGSFPSPQVPEEDVATRVIRAFNQQHSSYTTQHGVCNVDP
DCIPHLSRAGGWELFARELNPLIQAIIEFAKSIDGFMNLPQETQIQLLKGSVFELSLVFAAMYYNVDAQAVCGERYSV
PFACLIAEDDAEMQLIVEVNNTLQEIVHLQPHQSELALLAAGLILEQVSSSHGIGILDTATIATAETLKNALYQSVMPRI
GCMEDTIHRIQDVETRIRQTARLHQEALQNFRMSDPTSSEKLPALYKELFTADRP

>Eip75B RAR-like [*Drosophila melanogaster*]

MEAVQAAAAATSSGGSSGSVPGSGSGSASKLIKTEPIDFEMLHLEENERQQDIEREPSSSNSNSNSNSLTPQRYTHV
QVQTVPPRQPTGLTTPGGTQKVILTPRVEYVQQRATSSTGGGMKHVYSQQQGTAASRSAPPETTALLTTTSGTPQI
IITRTLPSNQHLSRRHSASPSALHHYQQQQPQRQQSPPPLHHQQQQQQQHVRVIRDGRLYDEATVVVAARRHSV
SPPPLHHHSRSAPVSPVIARRGGAAAYMDQQYQQRQTPPLAPPPPPPPPPPPPPPPQQQQQQYISTGVPPPTAAA
RKFVVSTSTRHVNVIASNHFQQQQQQHQAQQHQQQHQQHQQHQQHVIASVSSSSSSSAIGSGGSSSSHIFRTPV
VSSSSSSNMHHQQQQQQQQSSLGNSVMRPPPPPPPPKVKHASSSSSGNSSSSNTNNSSSSSNGEEPSSSIPDLEFD
GTTVLCRVCGDKASGFHYGVHSCEGCKGFFRRSIQQKIQYRPCTKNQQCSILRINRNRCQYCRLKKCIAVGMSRDAV
RFGRVPKREKARILAAMQQSTQNRGQQRALATELDDQPRLLAAVLRAHLETCEFTKEKVSAMRQRARDCPSYSMP
TLLACPLNPAPELQSEQEFSQRFAHVIRGVIDFAGMIPGFQLLTQDDKFTLLKAGLFDALFVRLICMFDSSINSIICLNG
QVMRRDAIQNGANARFLVDSTFNFAERMNSMNLTDAEIGLFCAIVLITPDRPGLRNLELIEKMYSRLKGCLQYIVAQ
NRPDQPEFLAKLLETMPDLRTLSTLHTEKLVVFRTEHKELLRQQMWSMEDGNNSDGQQNKSPSGSWADAMDVE
AAKSPLGSVSSTESADLDYGSPSSSQPQGVSLPSPPQQQPSALASSAPLLAATLSGGCPLRNRANSGSSGDSGAAEM
DIVGSHAHLTQNGLTITPIVRHQQQQQQQQQIGILNNAHSRNLNGGHAMCQQQQHPQLHHHLTAGAARYRK
LDSPTDSGIESGNEKNECKAVSSGGSSSCSSPRSSVDDALDCSDAAANHNQVVQHPQLSVVSVSPVRSPQPSTSSHL
KRQIVEDMPVLKRVLQAPPLYDTNSLMDEAYKPHKKFRALRHREFETAEADASSSTSGSNSLSAGSPRQSPVPNSVA
TPPPSAASAAAGNPAQSQLHMHLTRSSPKASMASSHSVLAKSLMAEPRMTPEQMKRSDIIQNYLKRENSTAASST
TNGVGNRSPSSSSTPPPSAVQNQQRWGSSSVITTTCQQRQQSVSPHSNGSSSSSSSSSSSSSSSSSTSSNCSSSSASS
CQYFQSPHSTSNGTSAPASSSSGSNSATPLLELQVDIADSAQPLNLSKKSPTPPPSKLHALVAAANAVQRYPTLSADV
TVTASNGGPPSAAASPAPSSSPPASVGSPNPGLSAAVHKVMLEA

>RXRa [*Homo sapiens*]

MDTKHFLPLDFSTQVNSSLTSPTGRGSMAAPSLHPSLGPGIGSPGQLHSPISTLSSPINGMGPPFSVISSPMGPHSM
SVPTTPTLGFSTGSPQLSSPMNPVSSSEDIKPPLGLNGVLKVPAHPSGNMASFTKHICAICGDRSSGKHYGVYSCEGC
KGFFKRTVRKDLTYTCRDNKDCLIDKRQRNRCQYCRYQKCLAMGMKREAVQEERQRGKDRNENEVESTSSANED
MPVERILEAELAVEPKTETYVEANMGLNPSSPNDPVTNICQAADKQLFTLVEWAKRIPHFSELPLDDQVILLRAGW
NELLIASFSHRSIAVKDGILLATGLHVHRNSAHSAGVGAIFDRVLTELVSKMRDMQMDKTELGCLRAIVLFNPDSKGL
SNPAEVEALREKVYASLEAYCKHKYPEQPGRFAKLLLRLPALRSIGLKCLEHLFFFKLIGDTPIDTFLMEMLEAPHQMT

>RARa [*Homo sapiens*]

MASNSSSCPTPGGGHLNGYPVPPYAFFFPPMLGGLSPPGALTTLQHQLPVSGYSTPSPATIETQSSSSEEIVPSPPSP
PPLPRIYKPCFVCQDKSSGYHYGVSACEGCKGFFRRSIQKNMVYTCHRDKNCIINKVTRNRCQYCRLQKCFEVGMSK
ESVRNDRNKKKKEVPKPECSESYTLTPEVGELIEKVRKAHQETFPALCQLGKYTTNNSSEQRVSLDIDLWDKFSELSTK
CIIKTVEFAKQLPGFTTLTIADQITLLKAACLDILILRICTRYTPEQDTMTFSDGLTLNRTQMHNAGFGPLTDLVFAFAN
QLLPLEMDDAETGLLSAICLICGDRQDLEQPDRVDMLQEPLLEALKVYVRKRRPSRPHMFPKMLMKITDLRSISAKG
AERVITLKMEIPGSMPPLIQEMLENSEGLDTLSGQPGGGGRDGGGLAPPPGSCSPSLSPSSNRSSPATHSP

>RARg [*Homo sapiens*]

MATNKERLFAAGALGPGSGYPGAGFPFAFPGALRGSPPFEMLSPSFRGLGQPDLPKEMASLSVETQSTSSEEMVPS
SPSPPPPPRVYKPCFVCNDKSSGYHYGVSSCEGCKGFFRRSIQKNMVYTCHRDKNCIINKVTRNRCQYCRLQKCFEV
GMSKEAVRNDRNKKKKEVKEEGSPDSYELSPQLEELITKVSKAHQETFPSLCQLGKYTTNSSADHRVQLDLGLWDKF
SELATKCIIKIVEFAKRLPGFTGLSIADQITLLKAACLDILMLRICTRYTPEQDTMTFSDGLTLNRTQMHNAGFGPLTDL
VFAFAGQLLPLEMDDTETGLLSAICLICGDRMDLEEPEKVDKLQEPLLEALRLYARRRRPSQPYMFPRMLMKITDLR
GISTKGAERAITLKMEIPGPMPPLIREMLENPEMFEDDSSQPGPHPNASSEDEVPGGQGKGGLKSPA

> RXR [*Xenopus laevis*]

MVGSAMTSSVNSPLGSIGSPFPVINCSVGSPGIPGTPSIGYGPVSSPQINSTVNLSGLHSVSSSEDVKPPLGMRSMPS
HPNGGAVSGKRLCAICGDRSSGKHYGVYSCEGCKGFFKRTIRKDLTYTCRDSKDCIVDKRQRNRCQYCRYQKCLATG
MKREAVQEERQRGKERDGEAELSGAINEEMPVEKILEAELAVEQKSDQSLEGGGSPSDPVTNICQAADKQLFTLVE
WAKRIPHFSELALDDQVILLRAGWNELLIASFSHRSISVKDGILLATGLHVHRNSAHSAGVGAIFDRVLTELVSKMRD
MRMDKTELGCLRAIILFNPDAKGLSNPGDVEVLREKVYASLESYCKQKYPDQQGRFAKLLLRLPALRSIGLKCLEHLFF
FKLIGDTPIDTFLMEMLEAPHQLS

>RARa [*Mus musculus*]

MASNSSSCPTPGGGHLNGYPVPPYAFFFPPMLGGLSPPGALTSLQHQLPVSGYSTPSPATIETQSSSSEEIVPSPPSP
PPLPRIYKPCFVCQDKSSGYHYGVSACEGCKGFFRRSIQKNMVYTCHRDKNCIINKVTRNRCQYCRLQKCFDVGMS
KESVRNDRNKKKKEAPKPECSESYTLTPEVGELIEKVRKAHQETFPALCQLGKYTTNNSSEQRVSLDIDLWDKFSELST
KCIIKTVEFAKQLPGFTTLTIADQITLLKAACLDILILRICTRYTPEQDTMTFSDGLTLNRTQMHNAGFGPLTDLVFAFA
NQLLPLEMDDAETGLLSAICLICGDRQDLEQPDKVDMLQEPLLEALKVYVRKRRPSRPHMFPKMLMKITDLRSISAK
GAERVITLKMEIPGSMPPLIQEMLENSEGLDTLSGQSGGGTRDGGGLAPPPGSCSPSLSPSSHRSSPATQSP

>RXRg [*Mus musculus*]

MYGNYSHFMKFPTGFGGSPGHTGSTSMSPSVALPTGKPMDSHPSYTDTPVSAPRTLSAVGTPLNALGSPYRVITSA
MGPPSGALAAPPGINLVAPPSSQLNVVNSVSSSEDIKPLPGLPGIGNMNYPSTSPGSLVKHICAICGDRSSGKHYGV
YSCEGCKGFFKRTIRKDLIYTCRDNKDCLIDKRQRNRCQYCRYQKCLVMGMKREAVQEERQRSRERAESEAECASSS
HEDMPVERILEAELAVEPKTESYGDMNVENSTNDPVTNICHAADKQLFTLVEWAKRIPHFSDLTLEDQVILLRAGW
NELLIASFSHRSVSVQDGILLATGLHVHRSSAHSAGVGSIFDRVLTELVSKMKDMQMDKSELGCLRAIVLFNPDAKG
LSNPSEVETLREKVYATLEAYTKQKYPEQPGRFAKLLLRLPALRSIGLKCLEHLFFFKLIGDTPIDSFLMEMLETPLQIT

>E 1-like NR [*Acropora millepora*]

MDSDYRIQAVDRKPIILCRVCGDRSSGKHYGVFTCDGCRGFFKRSIRRNLTYQCKERGNCTVDVTRRNQCQACRLK
KCFAVKMNKDAVQHERAPRSSQVVPVVPACSMLSGMYDQPPGQASEQQLVYHKSDSSAEAKRQCSPDSEADHT
TPENHKRCFSPPASLSQYHGQNNNQSPESSKKRSFLSIESLIETKNDVRQASVGNAHPSQNHVKDDNTDGPSAVYP
HSPETLYESAVHLLYMSVTWARNIPTFLDLPFRDQAILLEEGWSELFVLSAAQFSLPVEMGPLLSAAGLQVDKAPTD
KIVAGMADIRLLQNIIARFRRVQIDSTEYACLKAIVLFKPDLRGLRAPHMVERLQDQAQGMLGEYCRSKNPEQQVRF
GKLLLMLPSLRSVSPKTIEDLFFRGALDNVPIERMLCDMFKSS

>E 1-like NR [*Stylophora pistillata*]

MADADVLGARRGKSAKQTVLCKVCGDRASGKHYGVLTCDGCRGFFKRSIRRDLAYQCKENNSCPIDVARRNQCQ
ACRLKKCFEVRMNRDAVQHERAPRTNQFKQASNEEIRCKPLKRKHNSYEQENMDLPPGQIHVTPKKEKLLDSPVTP
EPIFYRNSPPRYSMEHKSLFLVGYSKAESPKDVPVAVSVPTATAMTPPHTPQHGQQVPYSIMYFSSPEMLHESAVRI
LFMTVKWVRNIPTFFDLPFRDQAILLEEGWSELFILSVAQWNLPVEIGTLLAAAGLNPERDNSDKSVCGTGEIKAMK
NIVERFKAANIDQTEYACLKAILLFKPDIRGLRAPGHVEQLQDQAQGMLGEYDRQTYPNQQVRFGRLLLILPGLRVL
SAKCIEQMFFRGTLDNIPMERLLSDMFKSA

Supplemental Table S6. Meiosis, RA uptake and DNA repair proteins

Additional Document (Excel file)

Supplemental Table S7. Overview of scRNA-seq features and metrics

Additional Document (Excel file)

Supplemental Table S8. Decomposition of Seurat´s clustering analysis of the scRNA-seq data

Additional Document (Excel file)

Supplemental Table S9. Marker genes and cluster identification

Additional Document (Excel file)

Supplemental Table S10. Gene Ontology (GO) terms of cluster marker genes

Additional Document (Excel file)

Supplemental Table S11. Transcription patterns of marker genes and genes predicted to interact with SmRAR by STRING

STRING

Your Input:

Smp_144170 *Rar-related orphan receptor alpha; RAR-like nuclear receptor (633 aa)*

Predicted Functional Partners:

		Neighborhood	Gene Fusion	Cooccurence	Coexpression	Experiments	Databases	Textmining	[Homology]	Score
Smp_163290	*Nuclear receptor co-repressor related (Ncor)*									0.552
Smp_123420	*Aryl hydrocarbon receptor nuclear translocator homolog (Darnt),, putative*									0.482
gli2a	*Transcriptional activator cubitus interruptus; Zinc finger transcription factor gli2*									0.478
Smp_172130	*E3 SUMO-protein ligase PIAS1; Putative sumo ligase*									0.438
Smp_153010	*Putative sumo ligase*									0.435
Smp_159540	*Putative inhibitor of apoptosis (Iap) domain family member ; Belongs to the ubiquitin-conjugating enzyme family*									0.431
Smp_142320	*Uncharacterized protein*									0.422
Smp_139200	*Nuclear hormone receptor nor-1/nor-2, putative*									0.402

Smp_number	annotation (Howe *et al.* 2017)	transcription pattern (Lu *et al.* 2016)	mO single cell atlas (this study)	single cell atlas (Wendt *et al.* 2021)
Smp_163290	Nuclear receptor co-repressor related (Ncor)	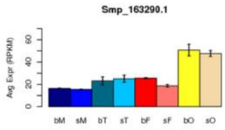	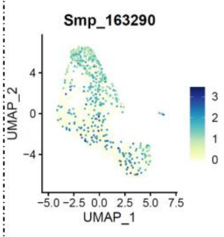	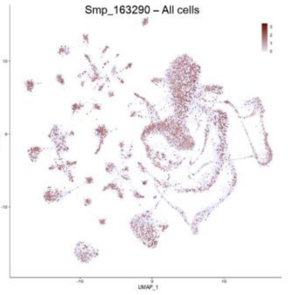
Smp_123420/ Smp_341950	Aryl hydrocarbon co-repressor homolog (Darnt, ARNT)	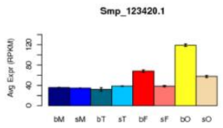	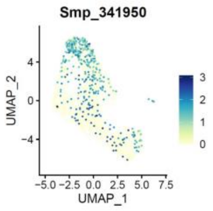	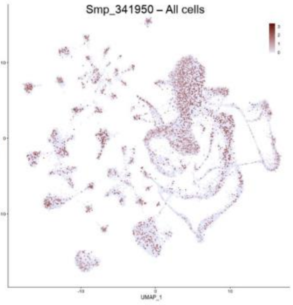
Smp_000530/ Smp_266960	Transcriptional activator cubitus interruptus;Zinc finger transcription factor gli2	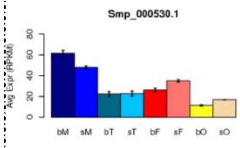	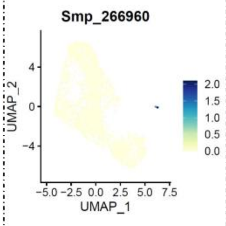	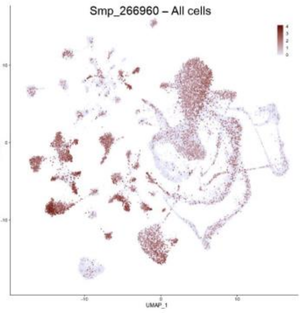

Smp_number	annotation (Howe *et al.* 2017)	transcription pattern (Lu *et al.* 2016)	mO single cell atlas (this study)	single cell atlas (Wendt *et al.* 2021)
Smp_172130	E3 SUMO-protein ligase PIAS1; Putative sumo ligase	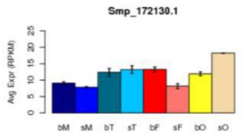	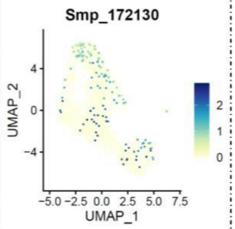	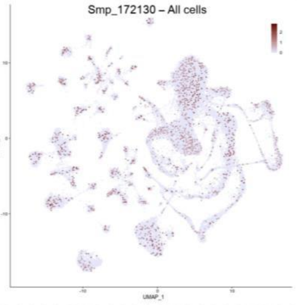
Smp_153010	E3 SUMO-protein ligase pli1; Putative sumo ligase	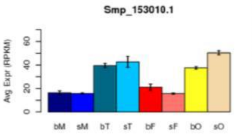	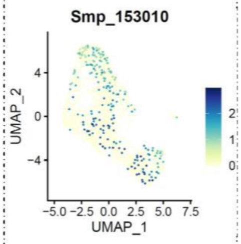	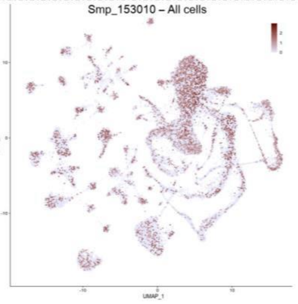
Smp_159540/ Smp_345080	UBC core domain-containing protein, Putative inhibitor of apoptosis (Iap) domain family member	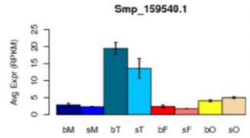	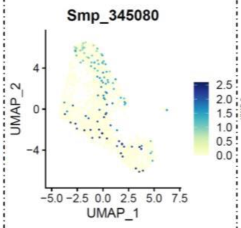	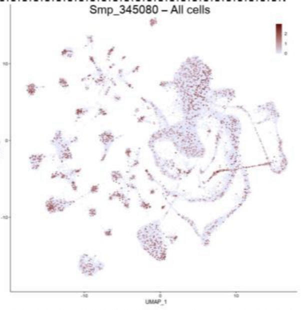
Smp_142320	SP-RING-type domain-containing protein	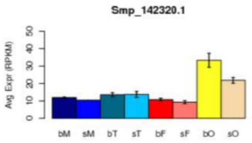	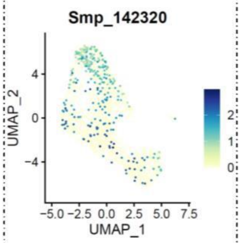	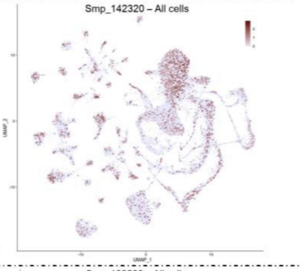
Smp_139200	Nuclear hormone receptor *nor*-1/*nor*-2	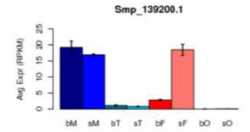	**not found**	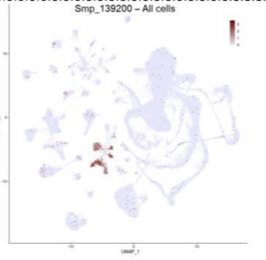

Smp_number	annotation (Howe *et al.* 2017)	transcription pattern (Lu *et al.* 2016)	mO single cell atlas (this study)	single cell atlas (Wendt *et al.* 2021)
**ovary marker (Khan and Newmark 2022)**				
Smp_078720<br>*S. mediterranea* ortholog: SMED30019646 (*lecg*)	Bone marrow proteoglycan, *bmpg*	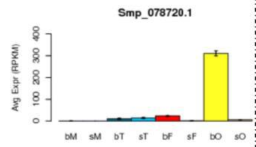	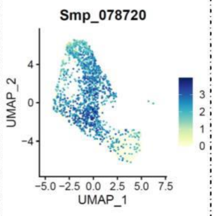	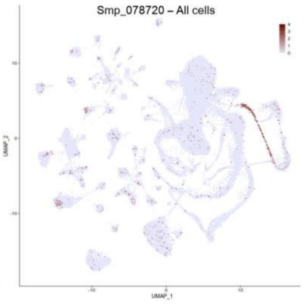
***S. mansoni* meiosis marker (Wendt *et al.* 2020; Nanes Sarfati *et al.* 2021)**				
Smp_333540/ Smp_162740	Meiosis-specific with OB domain-containing protein, *meiob*	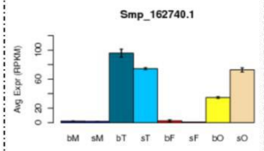	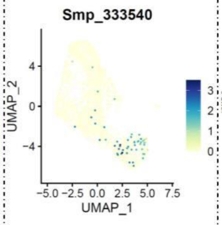	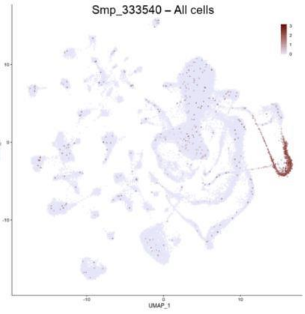
**genes involved in ovary maturation (Hahnel *et al.* 2014)**				
Smp_175590	SmFGFR-A, receptor protein-tyrosine kinase	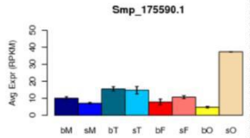	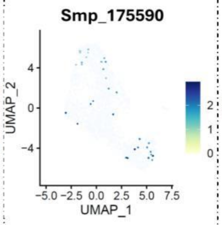	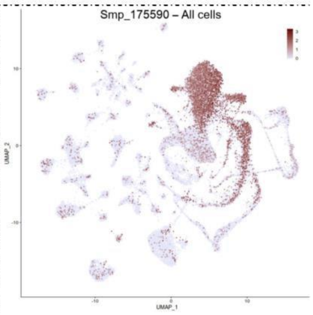
Smp_157300	SmFGFR-B, tyrosine kinase	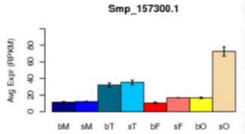	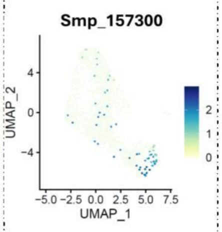	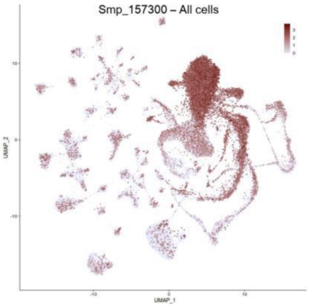

Supplemental Table S12. Overview of newly detected genes and their transcript profiles within the oocyte clusters

Additional Document (Excel file)
