# Supplementary material for: The retinoic acid family-like nuclear receptor SmRAR identified by single-cell transcriptomics of ovarian cells controls oocyte differentiation in Schistosoma mansoni
Source: Nucleic Acids Res. 2024 Dec 16;53(4):gkae1228. doi: 10.1093/nar/gkae1228 (PMC11879061; doi:10.1093/nar/gkae1228)
Supplement: gkae1228_Supplemental_Files [file gkae1228_supplemental_files.zip › Supplemental Table S11.pdf]

**Supplemental Table S11. Transcription patterns of marker genes and genes predicted to interact with SmRAR by STRING**

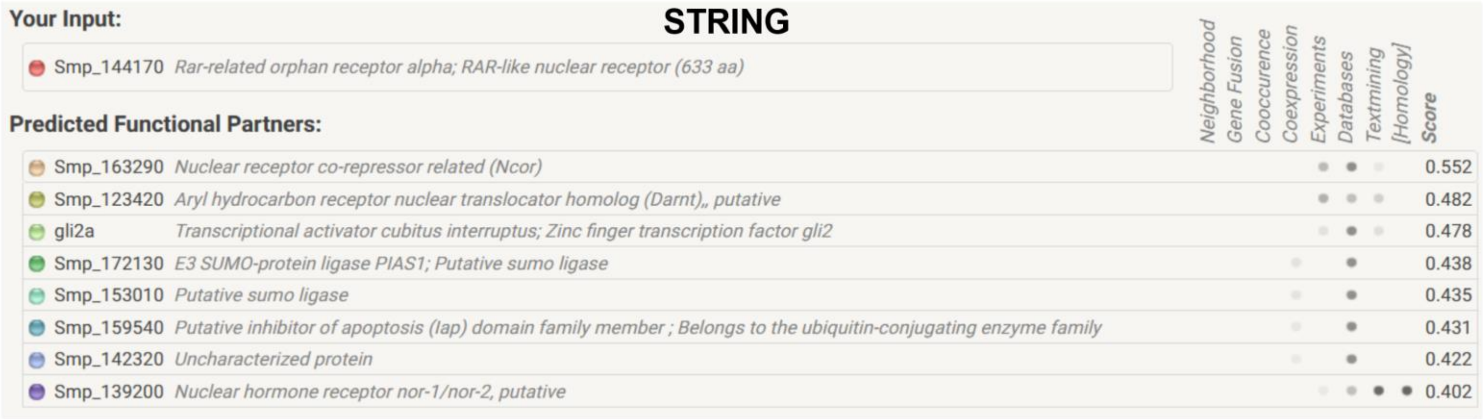

STRING

Your Input:

Smp_144170 Rar-related orphan receptor alpha; RAR-like nuclear receptor (633 aa)

Predicted Functional Partners:

| | | Score |
|---|---|---|
| Smp_163290 | Nuclear receptor co-repressor related (Ncor) | 0.552 |
| Smp_123420 | Aryl hydrocarbon receptor nuclear translocator homolog (Darnt),, putative | 0.482 |
| gli2a | Transcriptional activator cubitus interruptus; Zinc finger transcription factor gli2 | 0.478 |
| Smp_172130 | E3 SUMO-protein ligase PIAS1; Putative sumo ligase | 0.438 |
| Smp_153010 | Putative sumo ligase | 0.435 |
| Smp_159540 | Putative inhibitor of apoptosis (Iap) domain family member ; Belongs to the ubiquitin-conjugating enzyme family | 0.431 |
| Smp_142320 | Uncharacterized protein | 0.422 |
| Smp_139200 | Nuclear hormone receptor nor-1/nor-2, putative | 0.402 |



| Smp_number | annotation (Howe *et al.* 2017) | transcription pattern (Lu *et al.* 2016) | mO single cell atlas (this study) | single cell atlas (Wendt *et al.* 2021) |
|---|---|---|---|---|
| Smp_163290 | Nuclear receptor co-repressor related (Ncor) | 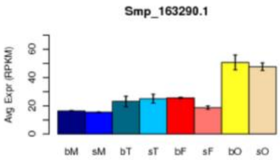  | 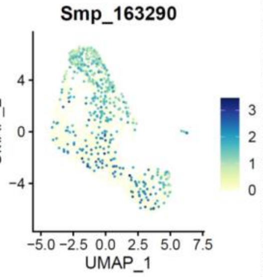  | 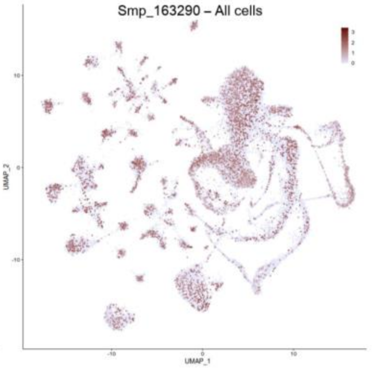  |
| Smp_123420/ Smp_341950 | Aryl hydrocarbon co-repressor homolog (Darnt, ARNT) | 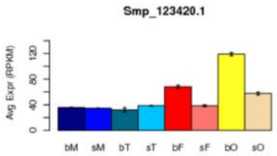  | 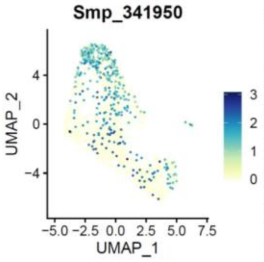  | 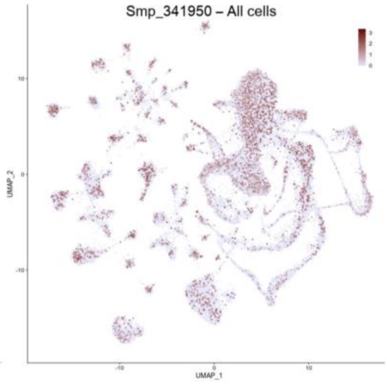  |
| Smp_000530/ Smp_266960 | Transcriptional activator cubitus interruptus;Zinc finger transcription factor gli2 | 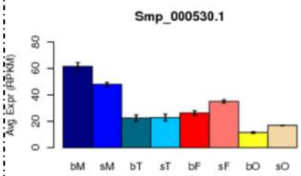  | 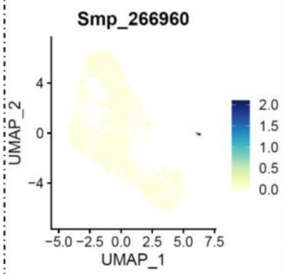  | 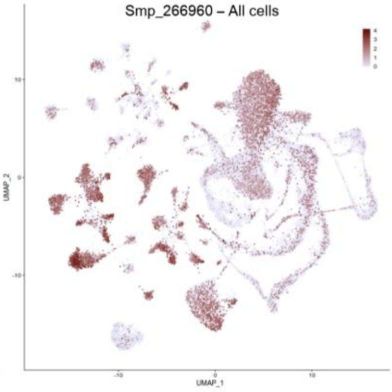  |

| Smp_number | annotation (Howe *et al.* 2017) | transcription pattern (Lu *et al.* 2016) | mO single cell atlas (this study) | single cell atlas (Wendt *et al.* 2021) |
|---|---|---|---|---|
| Smp_172130 | E3 SUMO-protein ligase PIAS1; Putative sumo ligase | 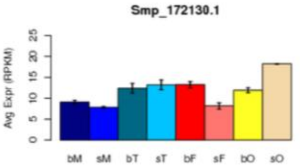  | 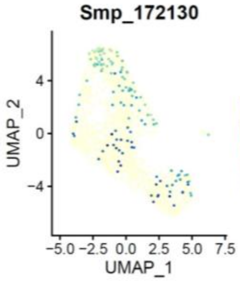  | 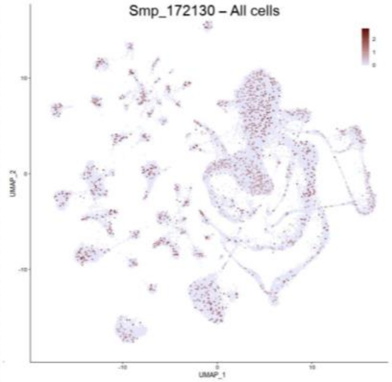  |
| Smp_153010 | E3 SUMO-protein ligase pli1; Putative sumo ligase | 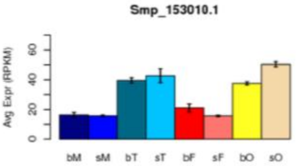  | 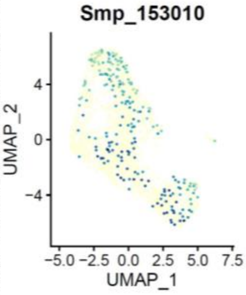  | 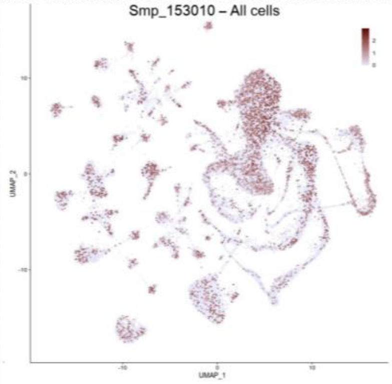  |
| Smp_159540/ Smp_345080 | UBC core domain-containing protein, Putative inhibitor of apoptosis (Iap) domain family member | 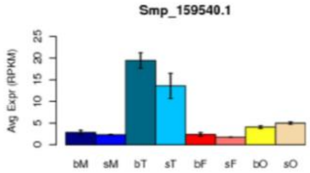  | 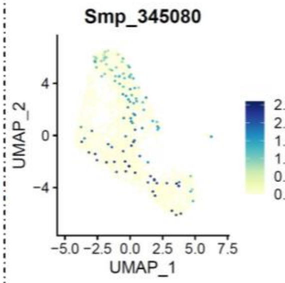  | 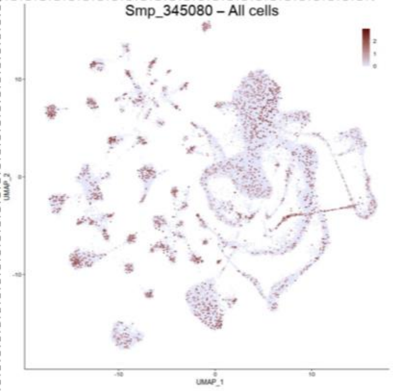  |
| Smp_142320 | SP-RING-type domain-containing protein | 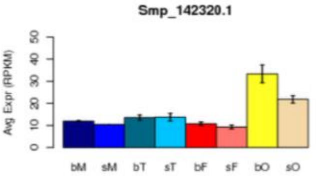  | 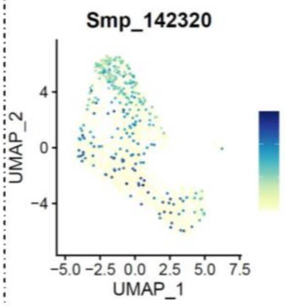  | 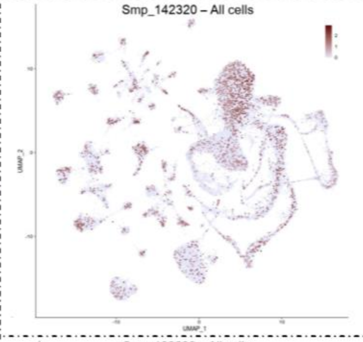  |
| Smp_139200 | Nuclear hormone receptor *nor*-1/*nor*-2 | 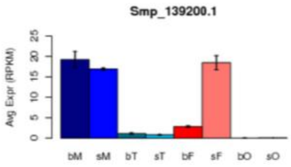  | **not found** | 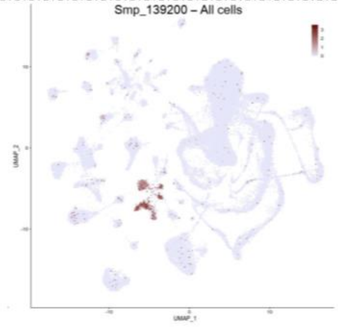  |

<table>
<tr><th>Smp_number</th><th>annotation<br>(Howe et al. 2017)</th><th>transcription pattern<br>(Lu et al. 2016)</th><th>mO single cell atlas<br>(this study)</th><th>single cell atlas<br>(Wendt et al. 2021)</th></tr>
<tr><td colspan="5">ovary marker (Khan and Newmark 2022)</td></tr>
<tr><td>Smp_078720<br><br>S. mediterranea ortholog: SMED30019646 (lecg)</td><td>Bone marrow proteoglycan, bmpg</td><td>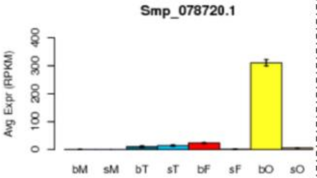
</td><td>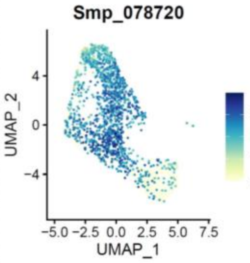
</td><td>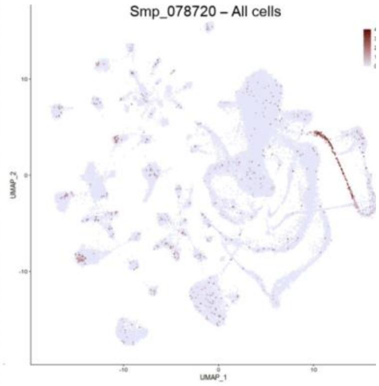
</td></tr>
<tr><td colspan="5">S. mansoni meiosis marker (Wendt et al. 2020; Nanes Sarfati et al. 2021)</td></tr>
<tr><td>Smp_333540/<br>Smp_162740</td><td>Meiosis-specific with OB domain-containing protein, meiob</td><td>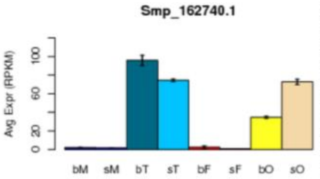
</td><td>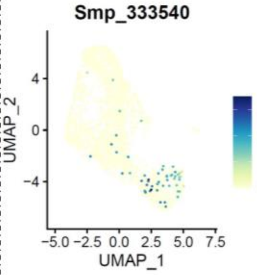
</td><td>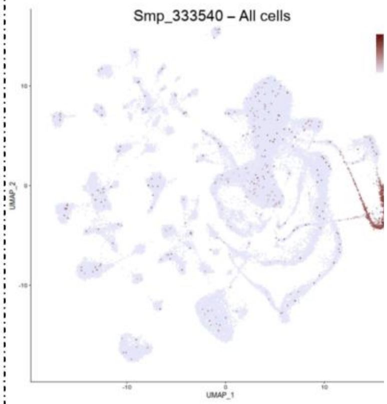
</td></tr>
<tr><td colspan="5">genes involved in ovary maturation (Hahnel et al. 2014)</td></tr>
<tr><td>Smp_175590</td><td>SmFGFR-A, receptor protein-tyrosine kinase</td><td>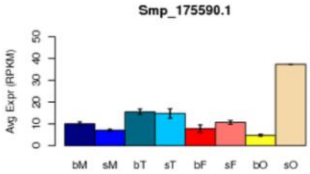
</td><td>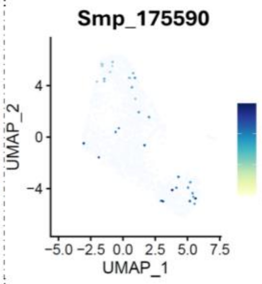
</td><td>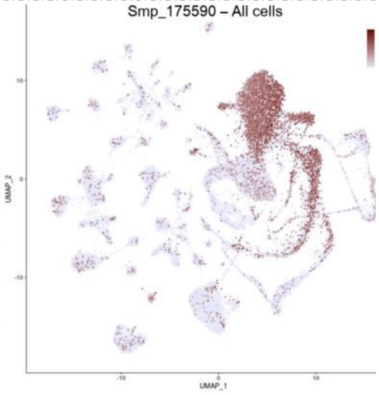
</td></tr>
<tr><td>Smp_157300</td><td>SmFGFR-B, tyrosine kinase</td><td>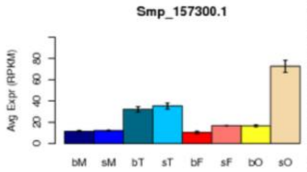
</td><td>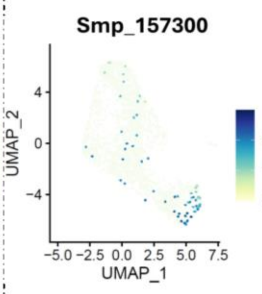
</td><td>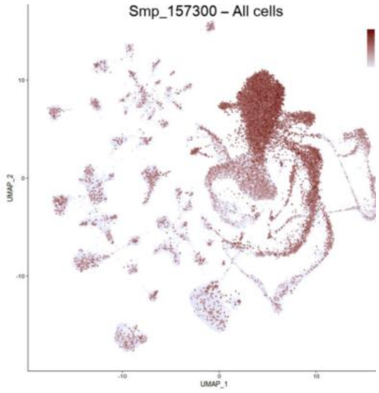
</td></tr>
</table>
