# Supplementary material for: The retinoic acid family-like nuclear receptor SmRAR identified by single-cell transcriptomics of ovarian cells controls oocyte differentiation in Schistosoma mansoni
Source: Nucleic Acids Res. 2024 Dec 16;53(4):gkae1228. doi: 10.1093/nar/gkae1228 (PMC11879061; doi:10.1093/nar/gkae1228)
Supplement: gkae1228_Supplemental_Files [file gkae1228_supplemental_files.zip › Supplemental Table S5.docx]

**Supplemental table S5. Amino acid sequence data of SmRAR and its para- and orthologs**

>Smp_144170 SmRAR [*Schistosoma mansoni*]

MSHCPVEYSNPNSQNTISYITSDSVISVSFADCQSSSPSYHSLSSSTALPGVGSFLDKSNHLPHYSPNKYYDTEPTAIPGHSYVENTDVFDNNLPYPIKSEAIHETVSGTFITPDAHNESCFTSPLQPLKSLNPSLPVQSSESLLSGFTHPLSMDHTVLEDGGLDLDRVFVAIINDRNSEHDIFNDNDVNCDSERCKKQLLNNSIQSSVNQSMLTDDSEDKSLLQYYSVIDSEKTTQHQTHPKTSPISEVEQTHKSSGSSEVSTRMCVVCGDKASGFHYGVSTCEGCKGFFRRAIQRDQSYTCAKNGTCEINKTLRNKCQQCRLLKCIAVGMSRDAVRKRRQGKKREQSSCESVTLPSSDSSNKYEGSAYSSCSADVVLTPSKAHLNETTPDPHEMPIKSINFQPSSSIPSSTNISLTLCNNNNNSNNSNNLTKEDQQTLDKFDRFLNYCKDQAIKYQANNWNISKSDYYHLSGSLSRHLSPIKHTPHELGESLKLLTVDEIFCPAQLKFTHEFACHLEQFIRLSQHDQAILLRDCLPELAILMLCRENRNKTHMNSSSTSINYQLNCLFSPWFPNAVVTDSSLDCLHMTCDSITAQSIFQFASRLQRLHLTNMEFGPLIGVILFTPERSDLLDIDFVNRTQNLWAELLRRYCESNGSQTRCAHLIMVLSTLRELASKITHNLNAWYKLSNTPMSNCLKEFLYSSSFNSMDDCF

>Smp_097700 RXR (retinoid-x)-like NR [*Schistosoma mansoni*]

MIMSIFANHDAIHMNHGVSDESVLYTHSYLIPPINQLENDILKPDQDMLTCVQSNRSHSPSQQIYDMLDSTVLSDNPIQTIANSSCKSQTSLKSSSCDTSNADLGVDIKPSFMYNDNPQVISQSFLTDLHNHTDIHTVSSDTNPNLCPIANSINSTHLQELVYNTPQKFPHMLPPDAHQHTVYSDNVVSEPFTRLLASGNSNETSTSYLPQVTKVETNSLTVSQSPILLFVDPNKTKPESRECFPTQNPSELASQSSSATSVNTTNLNPICVICGDKASGKHYGVISCEGCKGFFKRTVRKQLVYVCRESGQCPVDRRKRTRCQHCRFEQCLAKGMKKEAVQEERHRQPSSNPVPLISKPPKSEKKGPGRRSTFGNKSAESIVTDQPPNINQDSTPNISITPTTTDCVQPNQVKSESSTTCIQSNNVLLSDETDLPNLTLRCLLSAELSMDPKLAVSERGEAIYEDIPGDDDTGLHPLTIICQSIEQQLPRIVNWARQLPVFSSVYLSFDDQFCLIKAAWPELVLISSAYHSTVIRDGLLLSIGRHLGREVAKSHGLGPLVDRILHELVARFRDLSLQRTELALLRAIILFNPDANGLSSRHRVEAVREQLYSALHSYCTTNQPQDTSRFTKLLLRLPPLRSIASKCLEHLVFVKLAAEDPTSCRLINLVEHGVWPIQEKSFELATLPSDSASTDSVPSQITMVPTPQYIQHQDDNSLPTSSHTDLSYTHTLPNFHTVQNYPF

>Smp_073470 RXR (retinoid-x)-like NR [*Schistosoma mansoni*]

MIPVSIVTPQSVSSAEQIGEHSSSLSLQNVPVQQDSVSYLNSHTTVHMNDGQNEPSSIYLTDQELRLDSSFPNSPLGITRDPHCDDNGEEQNRISSFELISRLIDAEGLIELGYIPSSCNSAPAIDVASLSDLEEVTSKTLLWSSNCDDVDSFNFDDLSNKINESNTNNADTKLRSDNKTLEHRSALTTEYISVDFNPLNSTNALVPIISLPSDTNLQTIPSLESNEYENFQAHYKTNTVLENVQSLPSNTVPDDLGPLNITSYPMGLVEADIPSITRPSIHQSPVDHHALHPTIKPNHIPYSYSPLTPLSSVQIRSSIHESSFVIDNQVCPIDNICNQQLQTGSFVKNKYSSDYQEYSGAPECFPTVHTLELFTSHSSDLPHSSSHPSKNVGESPKHLSNDTKPIKYPELLQKIGENCNSNISGPNLPFISHSTYCSRTQSSSSCSIRQQLATAPSHPQLHVSIPQSPQSIYSVFSCSPNQKVAVSSTVNLPSSDSTSATLIFNGDFPSRPIISSTDNSFWSHPSIPSGSAVNTQMITNWEHSVPYPAYVPNSQITGFLPENISVTDKAAIYVKDGNSTAIQPVLGSGLVGSFQYLTPTTVLSKPNYTILNDQLTFNHPQETLIQPFLPQMSDPIKTTQNLRIPFCQGTQYTLTPGISIPINPEHSFGRLEMNTHRYSSESSKTNPALTSRSSSSRPFNSPVRRNSAPFQSTYQNCMNFSETAAHQSECSIVVSQLSTKKLTTPVLCCISSNSNNIPIISQQSPNNNNNNNNNNNVVFKTDNTKQHIIDQNILTKTATTTSIVQPSELCHIQPILISSPVKNNNNNTHNLGTGSYIDSNCLSNQSNNDKSHSITPSSPLLTNCNSPSTTLQSSTVCCLLRPSGRSTSTSSGSSCGSSCSGTGGGVSVSSGQYICSICSDRASGKHYGVFSCEGCKGFFKRTVRKELTYICRDSQECQIDKRLRNRCQYCRYQKCLRAGMRREAVQEERQQQQLQSEVQRSPTPPEQNCDLSVNSMIMSDTKITNAMNHSCLAEKQEIMLKTTNCTSSSSPHLLSNCSDSSINYFYSASNEKSQQPSINDNFNLTVNDAATYPPQELSLIGNKDSNNVTLPLADIHALKLPTTTSAAIPPPPDALEFIRTAESTISSRRKQWLSAFNKQQCHAEIAKCFQDSMENLKWLENNFEKCTTNHLPLFDLVIWSSKLPYICQLSCGVHLDLLKSACMQLIIVNLVYWLANDHKPRSLSTSNSTSKLPDTTPTINSTDISNITDDPPENSISDISKDCTIQMKKINKSVPLDEKMDYYYSNFPEFHLLNNLTKPMDNNNNDSISSKPTNINDNSVDDDMIRKRNTNVYKLIYNLAIKLRMLNLDPVELGCLKLILLLNPDSMTCLNNIRSLIELLRDQVYAGLEYYCNQVWPNAPHGRMGRLLLKLSNFQSVAARIEKLICSNELNNLLNNLESIFSYLSKKKVDHSNYISTTTTTTTTMSSTTTSTSNSIHLEFS

>Smp_105090 SmTR4 (vitamin-A-activated NR) [*Schistosoma mansoni*]

MYLHSPITTITSTDSSSTSTITTNTNSTSSLTVTSSPIDPSKLDIMNTMSNPTCCIPSNNIIQPPPIIPNHGLTSLLSNVASNLSAHQNHQNNFLKPSLVPTGRTSNAQLLQLLRNVTSSLPSSPVSYPISPRNTRNTPVLCDNNHTLSDSTSQVPIISTASSTAIPATDYSSCNNDQTLTLNSQSNRTSSNHPTVNGPSLLPDFETLLANSNPNMSDFIYCLVQSILFNHANTRQKFKSKKETPPLDSAALLSLLSGAGNLNPSSCSTGSLSNPLNPLSTLSSEIDSANCNIHLRQVLESLISLSVSPAFDQSSPHNLLSNDMLDSGSLSQTFQQLTNLSNRKATTGIQKVSPSGQILSNESSNSTNPPGNVSFDLLAQLSAAAAAAAVTPPPSNIVNQLGNLATDGFNPIFPTDENLSSALQNLLFKQMLSINTGSNAYTSHLGPGRDSVSSISQSGQYTGLKDCRSMSNDESNCSSEKLIPTTYSLASSFLSLHSPNSTNNCSNNNNPRSSFVRPSSRISPEPITLASSPPSSSSTPSFICPNQSNPPTGEFYPPNETVASSPNSLLLNGCTPVGTSPNTGLNMNQTAVNLSNDNQSWEPCKVCGDKASGRHYGVVSCEGCKGFFKRSIRGHVSYVCRSEQNCLVNKAYRNRCQYCRLQKCLAVGMRSEAVQNERRPTNTFALNFLNDNSGNSNCTTTGGSSNMNNGGSPSPSNNCCVSTPSITSTTTTTTTATVTTPTTTSANNSHTSTSGPSVTNVRNSSPQSQQLPYKIEPNSEELEDDRNNHSEFTTQKSEEGQSSSHSSPSGSSRSVPLTLSGATTVGLLSSSSGGVGSKHLDDNHFTGINKHSALSDISSSSSSSSYSATIADVEINDPNNASYLQHDIKPPVIQTSLGLKFTPGLCSSPQATSHSLQQPPAICLSTSASSFTRQRSLTSVSSSIVNNNSTAYASALPAYSNMLEGCHTTNHLDPDCGIANFSRCMNRNPLLSSQLFDIPDNKRGATQESYSRRMTSLSNTPCSSSATVSNHQDPLSIFDDTGGLNLNELTKLALNNINSLQSSSSASNQQGRSLAALYTYWLAAAAAAGSLNPLHHRHNDNHHHHQQQNHSVHLSTSNQNFNPLDMKTSLSSLKAGQPSTVTTTSIVGSNQNSTSQFSMKTNPNGLFGQHCNLENQLLINGLNHSSKSSLLSSSSSHSDALNMLMSIMLNSSDNVGLNNFNTLTDAVNSTTTHLQKAPINHHSHQHHPFMLQHHPHQHQLHMGSSNSHHSSTSPNTLEIPIPSTSSSCLSASLFGLNNTISTNNNTNNNNSNDNNSTMHTTVANNNSPFIPAPPPIKSTNLIQLLCKRTSNSQTSTLTDTDDKSKSGSCGSMVASTTNVRGRRSSNSSTANNMGENLHESGELIDSYRDANGHLLPKKFELTSSPDVTALRPENELLNPSPKKEETNWSRSRKRKMHYSFPSDVNNNARSIRRRTQSNNSLQPDTNNSIGLGDTRETCEDDDESRITECISECTKSPDDSRPNSPLVGPVLLNSMFDLNAHVFKAYQDNNNNRSLSISSELASRVLFLTVDWLRRFDGLKRLPIGVQRDLVAISWSDLFVLGLCQAADQINRSQNHRSNQESNDPQSTNSLCAQNDINSTRVKQISGEQQTNSHCSITPTSSFKSNSFDSSSSSHISPMKEPMDTSYLKTNCSSSSAVIELVEQLMKQFSGAEVDTHEYTYLRCMVILSSGRLCINARDASLAKQITEMESRVLSEFSEFLSTRAASSTTTGSLSSKRTKPIIKRVLILTQLLSTLRYLDPKDLEEAFFSNLLGSVSIAQILPYLLESNDLFTQSQLVMNSCLPDNSLDYKPGLNHVKLPNKQASRSGLEDSVTNFMGDSLPNNMTEKIDNLPSSLLLRPDQTSPQSRSASCEITATSIELNNQNTEPANSKSFVDDETNDSA

>Smp_036500 Smnhr-48 [*Schistosoma mansoni*]

MDPGANDYPSGNVNANLRSNSFRISSGTPLDNSMNINGPSSVGYGSNTGHDKESFLCLNRTQTDYVQNHQYIQSVHNDNTNNSHYCSNFEFSQSGSRKHPLSGVVRTRRRKAGISEEERVCIVCGEPASGYNFDRLTCESCKAFFRRNALKPRDKIKACNRGGGCAIEGNQRKHCPSCRLEKCLAVGMKRELILPPEKLEQRAKPRRRKHHSPQDSSSSPQNLLTGPSSVGSSNTNAYPPYLPSPLTTSSHPQHGSNQTSYNSSFVSGASRPLHLINPVKFSSYVNSSHCSDLSLTTDTFNSSSTLETKECNFPNSSIKIPVIGKSDELFMPPVNQDQNLNEKFLSLESSQQSDSFCKTRHGLCLIQCRKSGGRTFCQHRSCRLRSARKRRLRRRCTSSGSKCFRSPLMHITSPDPHLLSCLQGCVHRIREPFTPDEQLETGIAVTLEQEYNRMDACIRRIIRVVKHLPYFSEVGKPAQLSLLRTNIYGLIVLYSSFFFERDIRKLRYPVLQTDGQLTTVTVSMLDEVATPSTSVNDPDNVISGSYPKLLQSNSFRSFSRCRSNQANYAMGLREEFELYKSNTVAAFDHLDELVGSDDILRMSVLAIKLFSDNSLSEDLRPPVFAARQAYTLFLWNYIRWQAGSKRLRSATELYARILIAFIDLRTLEIRMTEFAKLLSLDGLSPLMREVCSQRSNLGSTSA

>RAR-like NR [*Schistosoma rodhaini*]

MSHCPVEYSNPNSQNTISYITSDSVISVSFADCQSSSPSYHSLSSSTALPGVGSFLDKSNHLPHYSPNKYYDTEPTAIPGHSYVENTDVFDNNLPYPIKSEAIHEIVSGTFITPDAHNESCFTSPLQPLKSLNPSLPVQSSESLLSGFTHPLSMNHTVLEDGGLDLDRVFVAIINDRNSEHDIFNDNDVNCDSERCKKQLLNNSIQSSVNQSMLTDDSEDKSLLQYYSVIDSEKTTQHQTHPKTSPVSEVEQTHKSSGSSEVSTRMCVVCGDKASGFHYGVSTCEGCKGFFRRAIQRDQSYTCAKNGTCEINKTLRNKCQQCRLLKCIAVGMSRDAVRKRRQGKKREQSSCESVTLPSSDSSNKYEGSAYSSCSANVVLTPSKARLNQTTPDPHEMPIKSINFQPSSSIPSSTNISLTLCSNNNSNNSNNLTKEDQQTLDKFDRFLNYCKDQAIKYQANNWNISKSDYYHLSGSLSRHLSPIKHTPHELGESLKLLTVDEIFCPAQLKFTHEFACHLEQFIRLSQHDQAILLRDCLPELAILMLCRENRNKTHLNSSSTSMNYQLNCLFSPWFPNAVVTDSSLDCLHMTCDSITAQSIFQFASRLQRLHLTNMEFGPLIGVILFTPERSDLLDIDFVNRTQNLWAELLRRYCESNGSQTRCAHLIMILSTLRELASKITHNLNAWYKLSNTPMSNCLKEFLYSSSFNSMDDCF

>RAR-like NR [*Schistosoma japonicum*]

MSNCPVEHLAPNCQNPISYIASDSVISVSFADCQSSSPSYHSLSSTTTLPGVGSFLDKSNNLPHYSPNKYYDTETNPLSKHPYVEETGFFDNSHPFPTKSECIQDLVSGTFITPATHSHSCFTSSLQPPKSLVPSFPVQSNETALNGSTHLLSVDHTILEDGGLDLDRIFVAIIGDQSSDHHVFNDADDVNDNGGNCLVDKSNSCNTQLLNNSMQSSVSQSMLTDDSGDKSLLHYYSVVEPEKTTQYQIHSETPVTEVEQPHKLSGSSEAPVRMCVVCGDKASGFHYGVSTCEGCKGFFRRAIQRDQSYTCAKNGTCEINKTLRNKCQQCRLLKCIAVGMSRDAVRKRRHGKKRQQSSCSDSAALPSSDSSSKYETATYSSNSTDVFLTPSKTHSNDEITLNSYEMPIKSSNLQSSASSSSLPPTTNPTLTLSNSSLTKEDQQTLDKFDHFLGYCKDQALKYQTNNWNISKSDHYSLSGSLSRHLSPIKHNSHELGESLLLTVDEIFCPAQLKFTHEFACHLEQFTRLSQHDQAILLRDCLPELAILMLCLENRNKVPMNSSSTFSSINHQLNCLFSPWFPNAVVTDSSLDCLHMTCNSITAQSIFQFASRLQRLHLTNMELGPLIGVILFTPERSDLLDIDFVNRTQNLWAELLRRYCESNGSQTRCAHLIMILSTLRELASKITHNLSGWYKLSQTPISNCLKEFLYSSGFNSADDCF

>RAR-like NR [*Schistosoma haematobium*]

MNPPNIEAAHIDLPIDVNPSTTEEIRMAIRQIKNGKAAGPDNIPAEALKSDIKATTSMLYLLFKKIWGEEQVPMDWKEGHLVKIPKKGYLSKCENYRGITLLSIPGKVFNRVLLNRMKDAVYAQLRDQQAGFRKDWSCTDQIATLRIIVEQSVEWNSSLYIKFIDYEKAFDSVDGRTLWKLLRHYGVLEKIVNIIRNSYDGPQCKVVHGGQLTDAFQVRTGVRQGCLLSPFLFLLVVDWITRTSTSEGKHGIQWTAQNQLDDLDFADDLALLSHTHEQIQMKKTSVAAVSASVGLNTNEKARSSNTTQRTPSNTITLNGETLEDVESFTYLGIIFDEPGGSDADTKATIGKMSHCPVEYSNPNSQNTISYITSDSVISVSFADCQSSSPSYHSLSSSTTLPGVGSFLDKSNHLSHYSPNKYYDTEPTVIPGHSYVENTGVFDNNLPYPIKSEAIHEIVSGTFITPDAHNQSCFTSSQPLKSLNPSLPVQSSESLLTGFTHPLSMDHTVLEDGGLDLDRVFVAIINDRNSEHDIFNDTDVNCDSERCSIDKSNSCNKQLLNNSIQSSVNQSMLTDDSEDKSLLQYYPVVDTEKTTQHQTHPKTSPISEVDQTRKSSGSSEVSTRMCVVCGDKASGFHYGVSTCEGCKGFFRRAIQRDQSYTCAKNGTCEINKTLRNKCQQCRLLKCIAVGMSRDAVRKRRHGKKREQSSCESVTLPSSDSSNKYETSAYSSGSADVVLTPSEAHLNETTLKPHEMPITSTNFQPSSSSIPSSTNISLTLCNNNNSDNSNGLTKEDQQTLDKFDRFLNYCKDQAIKYQANSWNISKSDYYHLSGSLSRHLSPIKHTPYELGESLKLLTVDEIFCPAQLKFTHEFACHLEQFIRLSQHDQSILLRDCLPELAILMLCRENRNKTHMNFSSTSVNYQLNCLFSPWFPNAVVTDSSLDCLHMTCDSITSQSIFQFASRLQRLHLTNMEFGPLIGVILFTPERSDLLDIDFVNRTQNLWAELLRRYCESNGSQTRCAHLIMILSTLRELASKITHNLNAWYKLSNTPMSNCLKEFLYSSSFNSMDDCF

>RXR (retinoid-x)-like NR [*Fasciola hepatica*]

MNINILKSEPDCVDPSLNEDSGLAAAYDLVLEHGDSFGDLTPESKTDLSRSDSFSFDEPRLVLPPVAQTFLRQDSAMPSSTLHYGVASPTTTSGSAGGVLPMQVCITDTHDSGKQPFEEIIGEHDHLPVVQSQWKFGGSGATVNCETPITNSVQLPTLSSTIQLSSDNSLKIESNPQTHSQVTTPTSNRKLNSGTSDSRRLAVAQSPAGTPYPSLTQAPMPSFSNLPLALCAICGDTASGRHYGVISCEGCKGFFKRAVRKQIQFTCRGSGQCPVDRSKRTRCQHCRLEQCLAKGMRREAVQEERHRYFQKTPKSRSKSRKADPLGFQPDLLLSMDSVSTELNGHTPKSIEHDSTVTTADPLQSSPTTVLCSGRKQNRANQPGRFATTSGTATGELMSASTAPPPLSLASLLTAELSTDAELPTTATGERVYVDIGDDGLDPLVVVCQSVEEQLTRLVCWARQLPVFTIPYFSTEDQLWLLRAAWAELLLISASFNSIAVRDGLLLANGRHLSRNKARQHGLGPLMDRFLLELVSRFREMSLERIELALLRAIILFNPDANNLCARQHVESVRESLYAGLHSYCTTTHPNDTSRFTKLLLRLPPLRSIAQKCLEHLVFVKLAAEDPSARRLINLVEHGVWPNDDRAFCPQQSQQQQIQHHHQQLQVKQTSPVGLHPQSAGPSTSIGPGSSGTVTRFSWPSQGIHNHPVPGTLLPHPMTNTNNNNSNHSHYNSTSGVPTTNTESAPTNGKANIKVDLDSLSQPIADNCSVE

>SmRAR-ortholog [*Dicrocoelium dendriticum*]

MNSHSDLLCFENASPSGHEFWNITKGAIETSSSNALGSDCSYHYPSLTPATEFLNDSIGTSHHVTTPCSISTPDHSTTTSEHVTYHQLQRLTKTSGMSIHAPSELSESLRLGLDCTENYLDINDHSQVPIYPQNHKDSLYIPTSNYQHSNSASPSSAPKEILYDLSHTHQLREVFHQPPYHPQASDLPTEGESICLDLDQVFSAIVSPEDESDVDPVKFNENASNSVDFHLKTCIPPAGDSIFPDDILFPLNSAQDAPRSSDLNPSTLPCIVAGDESLSVTHYPNCPQPLRILCDDREKDVYQRPLLTASHADSSSFPLENRPGFPLLNSSPVSPFSRQILSRSPRFCSAGPEQLSFSLSPRSALYSSSPMRSKHNSSCSMNTDGSVMTPQPFIHMSSVSSSPRFHQRSPSHLPWPHIQASITDARRMELAPIIPNLSPNRYLFGNTTRLTASIAEIPVDSTHMDSTVHHAARGSQPLGANVPKESLAHIRPRGAQSVSNPTIRFCEVCGDKSSGAHYGVYTCEGCKGFFRRAVQRNRTFSCARNGQCEVNRVLRNKCQHCRLHKCLASGMSKDSVRKKFDSDEKVSPLRRAKVRPTNARTIRKGGPIGVGFQITNPSDLHISRDNDPLPVPQHTTPERPQAPLTPSERPSFTSPTIMPPLSIDDRRMITSLFELFHASRKQAIIESGGEGHHVGAKGELTVIGSVEQLFCSAQLQFAHYFASCLGEFNQLSQHDQAVLLRGALVEITFLLLCNNHRCADAPPSIPTIHSGSVHSTSRCSYLLSPWNKSLILTEESFEHLHLSDNAWTPSRIFQFAERLTQMRLTSDEFGPLLGIVLFTPERANVLDIGAVNQIQGTWAELLRRLCESQGSYTRCAQLIMLLATVRELSGRLAHNLARWYRSRGAPFTECLQEFLLPVLSELVYL

>SmRAR-ortholog [*Opisthorchis viverrine*]

MLFVAFRLMSSPSDTAVLQSAISSKADWNYNSSSTNEIQPRAITSFESYLSDVEPVFDHQLSCYTPAIPSCTGSHLSKDTSDARQNQGCVTGEDLQQEYIPQSIVTNCSLLPTADSINYLPYYLSDRSIGSSVQSPTDTTSLNSRSPTHVTRVASTSDMILPSEPLVLSEGSVLSQSTVAPDESRSLDLDRVFSAIINPVSDIHFNPASLAVDPRNPVNHCSSLSSFVSSSGNSNVSENVAGSMNLERRRHLSGFPDSSFSSALSDASESLNSNSVDYVCESSHCRDIRPSFYTKAGTSGAHAPSSLCTIPSEGLSSSCPGTFDRSLHSAPDSLISTRSILSSCDSQINGVIQHQSNMLDTYSRSDRSVKNNHFTGVDPFTFSPSNESHSWNYQQASTTSCNKFPFATEFQLKRQEFSVDRPAPLSAQSESPFAADTRSSTENVGQTAQAGVLLRACVVCGDKSTGAHYGVFTCEGCKSRAATVQKSEQPYIRTSTLVTDKSRSVQSCTSTCTHIHLAVSALDTVRSLPLTAIWMLSSFILRHRSEMAQWLEREFAGWKLDTERVLQLNDFFISLDVLVKAPFPAPCTGWRRHKRDRPTTSLTAVTSEADFEFTRSMKEVTKRLGAVGATRLPGWGPRDHHCAWLETLQDMTANRCQWRSCCQFLSRLPELSNKSWLYGSEASMLNTDTYACARNGSCEVNRALRNKCQHCRFLKCLASGMSKDAVRRKQPAGNKKSSKTTGRRRSNKSAHTSSEHPEPMLPCDVNRSDACSSQTTPRSVYSGTEQSTVGWTAGSSISFPSALTNPLGKLSPTTNFLSPQDRQIIVSLHDLVRASKKHSLAETRMQMNSCLPESDGKIVITSVEQILCPAQLCFAYQFSGCLAEFAQLSQHDQAILIRGCLMELTFLLMCNNYRLAPEGESALCTGDGTEISTSEGECSAYLVSPWNANFLITEASFSHLHLTDNTWTPSRILKFAWRLTQLRLTDEEIGPLLGLVLFTPERADLLEVQAVSRIQGVWAELLRRLCE

>SMED-HNF4 [*Schmidtea mediterranea*]

MTSTQHPNLNPAFSYQLLTSNSAPISISSNASSMQYPLESEMSSQQHEVYASNASGPEGMENNQLCLICSDKATGKHYGAFSCDGCKGFFRRSVRKKNNYTCQYNRNCKMDKDKRNQCRYCRLKKCILVGMKRAAVQNERDRISTRRSSFDDIPPNVILSISQLMQAEQRVAIQKPPNPQEYMNRYADVPDVCESMKNQLFLLVNWAKSLPCFSQLNLSDQISLLKAHAGEILILGVIRRSFQLDEDDVLLLGNNLIISRNSSDKHFAEIASHILDDLFIPLRELQLDDAEFACLKAIVFFDPRVSESGKEFVRRCRYQIQMDLMNHMNDKQYHKPGRFGELLLTIPDLRLVTQLMVKKVEFMKMTGLAEIDSLLSETLLGDNPPGVFSIDPETDGNNNNNQNDSMDIINSHRISSPKYTYQDCYGIPPFMPDSVLLNINNQNISPEAASKIWSAYLPSSTSMPSGNIYRAFVNQGTEIMQQNSQDNSNFGMIINSDGMMLKNERVGVPSLFSCVNDSINLYQHSNLSQDTSHFLVNLDQGFPMINQSKYAYNGDANHNCSNSSNSDDLIHPALSVNSTQNVVDPSLLVAYTKSLPNSPIENCQIQSIANRSSEANQQHNSRSNYSAISSTAHFLTDHPIRIQESESSVFKKEEY

>SMED-TLX-1 [*Schmidtea mediterranea*]

MILSTGRILLDVPCKVCQDHSSGKHYGIYACDGCAGFFKRSIRHSRLYICKNKSIKGDSWIGICKIDKTHRNQCRACRLQKCVDSGMNKEAVQHERGPRSSTVRKKVAMYFNELSHNILDPSSMLPFTKSFNNANNVKEELLEFSLISAAKLNNFSNKNSFSISSLCDNSENSIETFDDCIGTPKSTIYFFQESYFSELSARSLFNTVHWIKSLQLPKEIVSVLLEGNWSQLFLLTAFETKVPFNDRSLMYRICDQVSSLSTMKQLDLINQCAVLHQHLSKLSLGSSEINILKQIIFLDLKSLNKKLEVAVQDACMDHVSRLCQLFDPTNKIAIEACLLMNIICIDPNFIHKLFFQKTIGQIPVLNLISDMIA

>RXRb [*Echinococcus granulosus*]

MQVWSNSNNASSVAPPANGDRDPMHSMPVTAESVPPTLNAFDSSIFASLISESDEKPLVDLKSDVISNADLQIGSAMPKPDLGSLYGGLQSSTQTNNTCDVATTHRTVFPANPQLQTLPCQSSCVSGSLNYPYTPTLYSPVTPVSPQYFQSAPPTVANHRALPHQQQGSYSLPPLRSYSYNQSFITGAYPKPYNYTIGSDGSISPYHRSVPSTSVKKVTYLCSARNCAICGATATGKHYGALSCDSCRAFFGTATHLGLRCECSGKSNIHDNNHQLRCHSCRLRKCLAVGMRKEAVRFEKSHAMLLYPNEKDYSSVAPSPASSICSHERADNLVGSRSSPATEATVKQIRAAEALIFDVPPLDLPESKPIFMDGKDCDSLQTDIQESIINLLLWSQKIPLFSDFSDSDRFILLRAGCIELLLVHFISRLANSLTESHSSTSLRSSNPPLQESSPSTSVTPIESTTTPPTPLNIPVVVVDVLFPFHSAPKDILFSQQAECDLRNADRWHESRVLMNILPVDVDHHPISITDVPNELNAQCLSRRLILCRLFDLARLFKRLCLSVEAVGCLRMVILFNPDVPELTEATRERVESRRDEAFICLEHTFVKADKKTALGRMAQMCLHLADLSFVAERIYSKTSSHPYPSFQCLVDLLECFYKSDPMATVSESST

>RAR-like NHR-23 [*Caenorhabditis elegans*]

MACKVTGPNRHLTSVLEMSSFVYWPRSRQQAHNFPMQAVEQKLADATRTLHAKSSLQPSLSIETPKSKENDESGCESSNCMFHPHTIKSEPNFCFAREFKSVPDDFRIGGGDLQMGNNSKRLTCVIDTNRVDMAGILPDNMSFRGLPENKSLLVSAQIEVIPCKVCGDKSSGVHYGVITCEGCKGFFRRSQSSIVNYQCPRQKNCVVDRVNRNRCQYCRLKKCIELGMSRDAVKFGRMSKKQREKVEDEVRMHKELAANGLGYQAIYGDYSPPPSHPSYCFDQSMYGHYPSGTSTPVNGYSIAVAATPTTPMPQNMYGATPSSTNGTQYVAHQATGGSFPSPQVPEEDVATRVIRAFNQQHSSYTTQHGVCNVDPDCIPHLSRAGGWELFARELNPLIQAIIEFAKSIDGFMNLPQETQIQLLKGSVFELSLVFAAMYYNVDAQAVCGERYSVPFACLIAEDDAEMQLIVEVNNTLQEIVHLQPHQSELALLAAGLILEQVSSSHGIGILDTATIATAETLKNALYQSVMPRIGCMEDTIHRIQDVETRIRQTARLHQEALQNFRMSDPTSSEKLPALYKELFTADRP

>Eip75B RAR-like [*Drosophila melanogaster*]

MEAVQAAAAATSSGGSSGSVPGSGSGSASKLIKTEPIDFEMLHLEENERQQDIEREPSSSNSNSNSNSLTPQRYTHVQVQTVPPRQPTGLTTPGGTQKVILTPRVEYVQQRATSSTGGGMKHVYSQQQGTAASRSAPPETTALLTTTSGTPQIIITRTLPSNQHLSRRHSASPSALHHYQQQQPQRQQSPPPLHHQQQQQQQHVRVIRDGRLYDEATVVVAARRHSVSPPPLHHHSRSAPVSPVIARRGGAAAYMDQQYQQRQTPPLAPPPPPPPPPPPPPPPQQQQQQYISTGVPPPTAAARKFVVSTSTRHVNVIASNHFQQQQQQHQAQQHQQQHQQHQQHQQHVIASVSSSSSSSAIGSGGSSSSHIFRTPVVSSSSSSNMHHQQQQQQQQSSLGNSVMRPPPPPPPPKVKHASSSSSGNSSSSNTNNSSSSSNGEEPSSSIPDLEFDGTTVLCRVCGDKASGFHYGVHSCEGCKGFFRRSIQQKIQYRPCTKNQQCSILRINRNRCQYCRLKKCIAVGMSRDAVRFGRVPKREKARILAAMQQSTQNRGQQRALATELDDQPRLLAAVLRAHLETCEFTKEKVSAMRQRARDCPSYSMPTLLACPLNPAPELQSEQEFSQRFAHVIRGVIDFAGMIPGFQLLTQDDKFTLLKAGLFDALFVRLICMFDSSINSIICLNGQVMRRDAIQNGANARFLVDSTFNFAERMNSMNLTDAEIGLFCAIVLITPDRPGLRNLELIEKMYSRLKGCLQYIVAQNRPDQPEFLAKLLETMPDLRTLSTLHTEKLVVFRTEHKELLRQQMWSMEDGNNSDGQQNKSPSGSWADAMDVEAAKSPLGSVSSTESADLDYGSPSSSQPQGVSLPSPPQQQPSALASSAPLLAATLSGGCPLRNRANSGSSGDSGAAEMDIVGSHAHLTQNGLTITPIVRHQQQQQQQQQIGILNNAHSRNLNGGHAMCQQQQQHPQLHHHLTAGAARYRKLDSPTDSGIESGNEKNECKAVSSGGSSSCSSPRSSVDDALDCSDAAANHNQVVQHPQLSVVSVSPVRSPQPSTSSHLKRQIVEDMPVLKRVLQAPPLYDTNSLMDEAYKPHKKFRALRHREFETAEADASSSTSGSNSLSAGSPRQSPVPNSVATPPPSAASAAAGNPAQSQLHMHLTRSSPKASMASSHSVLAKSLMAEPRMTPEQMKRSDIIQNYLKRENSTAASSTTNGVGNRSPSSSSTPPPSAVQNQQRWGSSSVITTTCQQRQQSVSPHSNGSSSSSSSSSSSSSSSSSTSSNCSSSSASSCQYFQSPHSTSNGTSAPASSSSGSNSATPLLELQVDIADSAQPLNLSKKSPTPPPSKLHALVAAANAVQRYPTLSADVTVTASNGGPPSAAASPAPSSSPPASVGSPNPGLSAAVHKVMLEA

>RXRa [*Homo sapiens*]

MDTKHFLPLDFSTQVNSSLTSPTGRGSMAAPSLHPSLGPGIGSPGQLHSPISTLSSPINGMGPPFSVISSPMGPHSMSVPTTPTLGFSTGSPQLSSPMNPVSSSEDIKPPLGLNGVLKVPAHPSGNMASFTKHICAICGDRSSGKHYGVYSCEGCKGFFKRTVRKDLTYTCRDNKDCLIDKRQRNRCQYCRYQKCLAMGMKREAVQEERQRGKDRNENEVESTSSANEDMPVERILEAELAVEPKTETYVEANMGLNPSSPNDPVTNICQAADKQLFTLVEWAKRIPHFSELPLDDQVILLRAGWNELLIASFSHRSIAVKDGILLATGLHVHRNSAHSAGVGAIFDRVLTELVSKMRDMQMDKTELGCLRAIVLFNPDSKGLSNPAEVEALREKVYASLEAYCKHKYPEQPGRFAKLLLRLPALRSIGLKCLEHLFFFKLIGDTPIDTFLMEMLEAPHQMT

>RARa [*Homo sapiens*]

MASNSSSCPTPGGGHLNGYPVPPYAFFFPPMLGGLSPPGALTTLQHQLPVSGYSTPSPATIETQSSSSEEIVPSPPSPPPLPRIYKPCFVCQDKSSGYHYGVSACEGCKGFFRRSIQKNMVYTCHRDKNCIINKVTRNRCQYCRLQKCFEVGMSKESVRNDRNKKKKEVPKPECSESYTLTPEVGELIEKVRKAHQETFPALCQLGKYTTNNSSEQRVSLDIDLWDKFSELSTKCIIKTVEFAKQLPGFTTLTIADQITLLKAACLDILILRICTRYTPEQDTMTFSDGLTLNRTQMHNAGFGPLTDLVFAFANQLLPLEMDDAETGLLSAICLICGDRQDLEQPDRVDMLQEPLLEALKVYVRKRRPSRPHMFPKMLMKITDLRSISAKGAERVITLKMEIPGSMPPLIQEMLENSEGLDTLSGQPGGGGRDGGGLAPPPGSCSPSLSPSSNRSSPATHSP

>RARg [*Homo sapiens*]

MATNKERLFAAGALGPGSGYPGAGFPFAFPGALRGSPPFEMLSPSFRGLGQPDLPKEMASLSVETQSTSSEEMVPSSPSPPPPPRVYKPCFVCNDKSSGYHYGVSSCEGCKGFFRRSIQKNMVYTCHRDKNCIINKVTRNRCQYCRLQKCFEVGMSKEAVRNDRNKKKKEVKEEGSPDSYELSPQLEELITKVSKAHQETFPSLCQLGKYTTNSSADHRVQLDLGLWDKFSELATKCIIKIVEFAKRLPGFTGLSIADQITLLKAACLDILMLRICTRYTPEQDTMTFSDGLTLNRTQMHNAGFGPLTDLVFAFAGQLLPLEMDDTETGLLSAICLICGDRMDLEEPEKVDKLQEPLLEALRLYARRRRPSQPYMFPRMLMKITDLRGISTKGAERAITLKMEIPGPMPPLIREMLENPEMFEDDSSQPGPHPNASSEDEVPGGQGKGGLKSPA

> RXR [*Xenopus laevis*]

MVGSAMTSSVNSPLGSIGSPFPVINCSVGSPGIPGTPSIGYGPVSSPQINSTVNLSGLHSVSSSEDVKPPLGMRSMPSHPNGGAVSGKRLCAICGDRSSGKHYGVYSCEGCKGFFKRTIRKDLTYTCRDSKDCIVDKRQRNRCQYCRYQKCLATGMKREAVQEERQRGKERDGEAELSGAINEEMPVEKILEAELAVEQKSDQSLEGGGSPSDPVTNICQAADKQLFTLVEWAKRIPHFSELALDDQVILLRAGWNELLIASFSHRSISVKDGILLATGLHVHRNSAHSAGVGAIFDRVLTELVSKMRDMRMDKTELGCLRAIILFNPDAKGLSNPGDVEVLREKVYASLESYCKQKYPDQQGRFAKLLLRLPALRSIGLKCLEHLFFFKLIGDTPIDTFLMEMLEAPHQLS

>RARa [*Mus musculus*]

MASNSSSCPTPGGGHLNGYPVPPYAFFFPPMLGGLSPPGALTSLQHQLPVSGYSTPSPATIETQSSSSEEIVPSPPSPPPLPRIYKPCFVCQDKSSGYHYGVSACEGCKGFFRRSIQKNMVYTCHRDKNCIINKVTRNRCQYCRLQKCFDVGMSKESVRNDRNKKKKEAPKPECSESYTLTPEVGELIEKVRKAHQETFPALCQLGKYTTNNSSEQRVSLDIDLWDKFSELSTKCIIKTVEFAKQLPGFTTLTIADQITLLKAACLDILILRICTRYTPEQDTMTFSDGLTLNRTQMHNAGFGPLTDLVFAFANQLLPLEMDDAETGLLSAICLICGDRQDLEQPDKVDMLQEPLLEALKVYVRKRRPSRPHMFPKMLMKITDLRSISAKGAERVITLKMEIPGSMPPLIQEMLENSEGLDTLSGQSGGGTRDGGGLAPPPGSCSPSLSPSSHRSSPATQSP

>RXRg [*Mus musculus*]

MYGNYSHFMKFPTGFGGSPGHTGSTSMSPSVALPTGKPMDSHPSYTDTPVSAPRTLSAVGTPLNALGSPYRVITSAMGPPSGALAAPPGINLVAPPSSQLNVVNSVSSSEDIKPLPGLPGIGNMNYPSTSPGSLVKHICAICGDRSSGKHYGVYSCEGCKGFFKRTIRKDLIYTCRDNKDCLIDKRQRNRCQYCRYQKCLVMGMKREAVQEERQRSRERAESEAECASSSHEDMPVERILEAELAVEPKTESYGDMNVENSTNDPVTNICHAADKQLFTLVEWAKRIPHFSDLTLEDQVILLRAGWNELLIASFSHRSVSVQDGILLATGLHVHRSSAHSAGVGSIFDRVLTELVSKMKDMQMDKSELGCLRAIVLFNPDAKGLSNPSEVETLREKVYATLEAYTKQKYPEQPGRFAKLLLRLPALRSIGLKCLEHLFFFKLIGDTPIDSFLMEMLETPLQIT

>E 1-like NR [*Acropora millepora*]

MDSDYRIQAVDRKPIILCRVCGDRSSGKHYGVFTCDGCRGFFKRSIRRNLTYQCKERGNCTVDVTRRNQCQACRLKKCFAVKMNKDAVQHERAPRSSQVVPVVPACSMLSGMYDQPPGQASEQQLVYHKSDSSAEAKRQCSPDSEADHTTPENHKRCFSPPASLSQYHGQNNNQSPESSKKRSFLSIESLIETKNDVRQASVGNAHPSQNHVKDDNTDGPSAVYPHSPETLYESAVHLLYMSVTWARNIPTFLDLPFRDQAILLEEGWSELFVLSAAQFSLPVEMGPLLSAAGLQVDKAPTDKIVAGMADIRLLQNIIARFRRVQIDSTEYACLKAIVLFKPDLRGLRAPHMVERLQDQAQGMLGEYCRSKNPEQQVRFGKLLLMLPSLRSVSPKTIEDLFFRGALDNVPIERMLCDMFKSS

>E 1-like NR [*Stylophora pistillata*]

MADADVLGARRGKSAKQTVLCKVCGDRASGKHYGVLTCDGCRGFFKRSIRRDLAYQCKENNSCPIDVARRNQCQACRLKKCFEVRMNRDAVQHERAPRTNQFKQASNEEIRCKPLKRKHNSYEQENMDLPPGQIHVTPKKEKLLDSPVTPEPIFYRNSPPRYSMEHKSLFLVGYSKAESPKDVPVAVSVPTATAMTPPHTPQHGQQVPYSIMYFSSPEMLHESAVRILFMTVKWVRNIPTFFDLPFRDQAILLEEGWSELFILSVAQWNLPVEIGTLLAAAGLNPERDNSDKSVCGTGEIKAMKNIVERFKAANIDQTEYACLKAILLFKPDIRGLRAPGHVEQLQDQAQGMLGEYDRQTYPNQQVRFGRLLLILPGLRVLSAKCIEQMFFRGTLDNIPMERLLSDMFKSA
