# Supplementary figures and images for: The retinoic acid family-like nuclear receptor SmRAR identified by single-cell transcriptomics of ovarian cells controls oocyte differentiation in Schistosoma mansoni
Source: Nucleic Acids Res. 2024 Dec 16;53(4):gkae1228. doi: 10.1093/nar/gkae1228 (PMC11879061; doi:10.1093/nar/gkae1228)

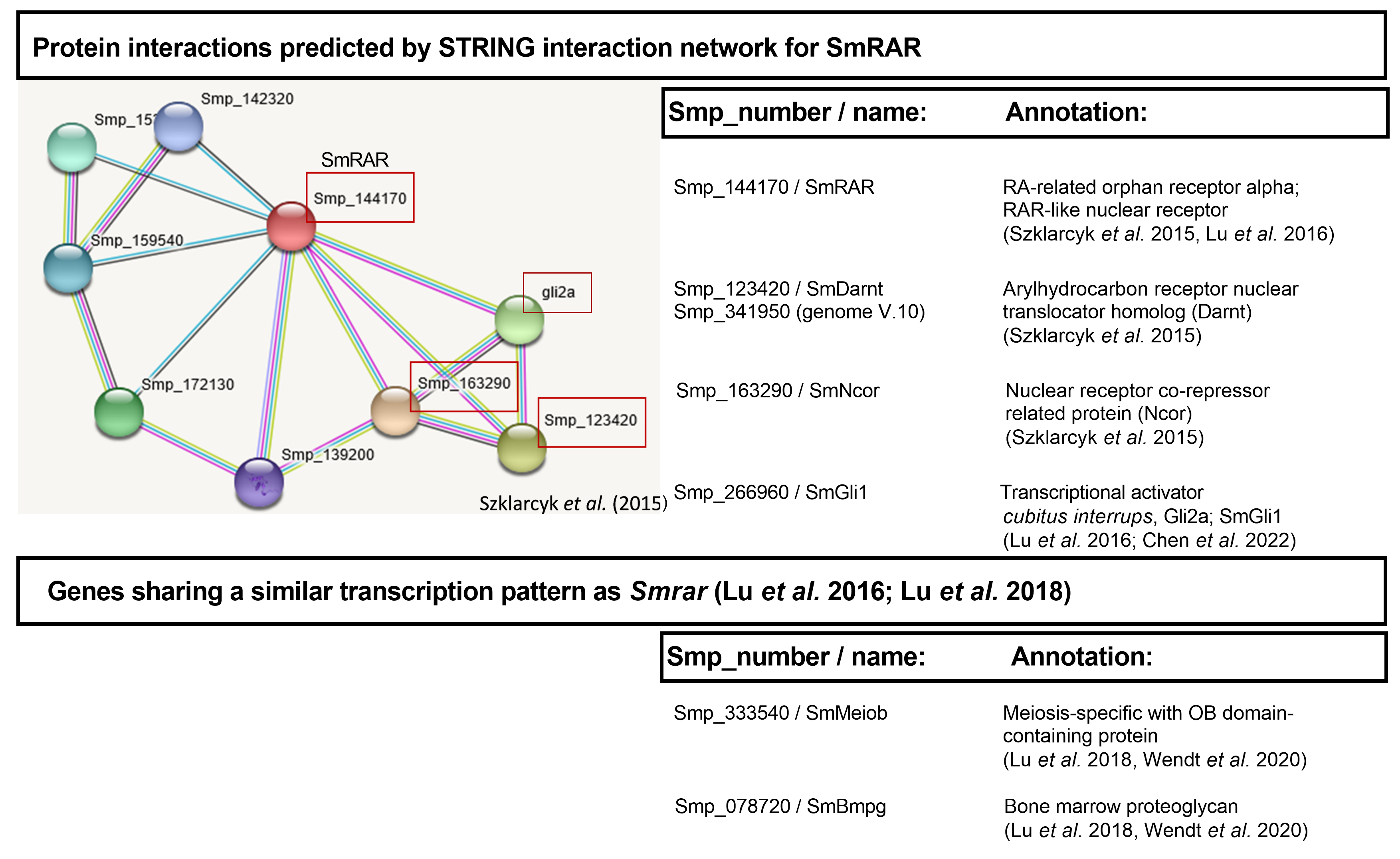

Supplement: gkae1228_Supplemental_Files [file gkae1228_supplemental_files.zip › Supplemental Table S3.bmp]
